# Plasma proteomic profiling suggests an association between antigen driven clonal B cell expansion and ME/CFS

**DOI:** 10.1371/journal.pone.0236148

**Published:** 2020-07-21

**Authors:** Milica Milivojevic, Xiaoyu Che, Lucinda Bateman, Aaron Cheng, Benjamin A. Garcia, Mady Hornig, Manuel Huber, Nancy G. Klimas, Bohyun Lee, Hyoungjoo Lee, Susan Levine, Jose G. Montoya, Daniel L. Peterson, Anthony L. Komaroff, W. Ian Lipkin

**Affiliations:** 1 Center for Infection and Immunity, Columbia University Mailman School of Public Health, New York, NY, United States of America; 2 Department of Biostatistics, Columbia University Mailman School of Public Health, New York, NY, United States of America; 3 Bateman Horne Center, Salt Lake City, UT, United States of America; 4 Perelman School of Medicine, University of Pennsylvania, Philadelphia, PA, United States of America; 5 Department of Epidemiology, Columbia University Mailman School of Public Health, New York, NY, United States of America; 6 German Research Center for Environmental Health, Institute for Health Economics and Health Care Management, Helmholtz Zentrum München, Neuherberg, Germany; 7 Institute for Neuro Immune Medicine, College of Osteopathic Medicine, Nova Southeastern University, Fort Lauderdale, FL, United States of America; 8 Miami VA Medical Center, Miami, FL, United States of America; 9 Levine Clinic, New York, NY, United States of America; 10 Palo Alto Medical Foundation, Jack S. Remington Laboratory for Specialty Diagnostics of Toxoplasmosis, Palo Alto, CA, United States of America; 11 Sierra Internal Medicine at Incline Village, Incline Village, NV, United States of America; 12 Harvard Medical School, Brigham and Women's Hospital, Boston, MA, United States of America; National Institutes of Health, UNITED STATES

## Abstract

Myalgic Encephalomyelitis/Chronic Fatigue Syndrome (ME/CFS) is an unexplained chronic, debilitating illness characterized by fatigue, sleep disturbances, cognitive dysfunction, orthostatic intolerance and gastrointestinal problems. Using ultra performance liquid chromatography-tandem mass spectrometry (UPLC-MS/MS), we analyzed the plasma proteomes of 39 ME/CFS patients and 41 healthy controls. Logistic regression models, with both linear and quadratic terms of the protein levels as independent variables, revealed a significant association between ME/CFS and the immunoglobulin heavy variable (IGHV) region 3-23/30. Stratifying the ME/CFS group based on self-reported irritable bowel syndrome (sr-IBS) status revealed a significant quadratic effect of immunoglobulin lambda constant region 7 on its association with ME/CFS with sr-IBS whilst IGHV3-23/30 and immunoglobulin kappa variable region 3–11 were significantly associated with ME/CFS without sr-IBS. In addition, we were able to predict ME/CFS status with a high degree of accuracy (AUC = 0.774–0.838) using a panel of proteins selected by 3 different machine learning algorithms: Lasso, Random Forests, and XGBoost. These algorithms also identified proteomic profiles that predicted the status of ME/CFS patients with sr-IBS (AUC = 0.806–0.846) and ME/CFS without sr-IBS (AUC = 0.754–0.780). Our findings are consistent with a significant association of ME/CFS with immune dysregulation and highlight the potential use of the plasma proteome as a source of biomarkers for disease.

## Introduction

Myalgic encephalomyelitis/chronic fatigue syndrome (ME/CFS) is a debilitating disease of unknown cause that affects up to 2.5 million people in the USA alone [1]. The disease is defined by persistent fatigue lasting longer than six months, post-exertional malaise, unrefreshing sleep, and either cognitive dysfunction or orthostatic intolerance [1]. These symptoms are often accompanied by others that may include chronic pain, influenza-like symptoms, and gastro-intestinal disturbances [2]. Whilst there is no known cause of ME/CFS, multiple studies have reported immune, metabolic, and neurological disturbances. There are no approved diagnostic tests [3, 4].

Mass spectrometry analysis of plasma has identified disturbances in energy, amino acid, and lipid metabolism [5–10]. Proteomic reports are limited to two studies of cerebrospinal fluid [11, 12], one study of saliva [13], and one study of platelet mitochondria [14]. Baraniuk et al. (2005) [11] found that innate immune and amyloidogenic proteins were detected more frequently in the cerebrospinal fluid of patients compared to controls, and that the presence of one or more of a subset of proteins (α-1-macroglobulin, amyloid precursor-like protein 1, keratin 16, orosomucoid 2, and pigment epithelium-derived factor) predicted ME/CFS status with 80% accuracy. Another study of cerebrospinal fluid by Schutzer et al (2011) [12] showed differing proteomic profiles between ME/CFS and post-treatment Lyme disease patients that included enrichment in the ME/CFS group of proteins involved in the complement cascade as well as pathways related to CDK5 signaling and dopamine signaling. Ciergia et al (2013) [13] studied the saliva of monozygotic twins discordant for ME/CFS and found 13 differentially expressed proteins related to inflammation and metabolism. Another study of the platelet mitochondrial proteome in the same pair of twins identified upregulation of aconitate hydratase, ATP synthase subunit beta, and malate dehydrogenase that was replicated in saliva of a larger cohort of 45 subjects with ME/CFS [14].

Here we report a ME/CFS-related plasma proteome analysis using untargeted ultra-performance liquid chromatography-tandem mass spectrometry (UPLC-MS/MS). We identified differing profiles between ME/CFS patient, as well as ME/CFS subgroups, based on their self-reported irritable bowel syndrome (sr-IBS) status, and controls. In addition, we identify a set of proteins that may predict ME/CFS status.

## Materials and methods

### Study population

50 ME/CFS cases and 50 controls were collected in ME/CFS clinics in New York, NY; Salt Lake City, UT; Sierra, NV; and Miami, FL [15]. All ME/CFS cases met the 1994 CDC Fukuda [16] and/or Canadian consensus criteria for ME/CFS [17]. Controls were frequency-matched to cases on age, sex, race/ethnicity, geographic/clinical site, and season of sampling [18]. All ME/CFS cases completed standardized screening and assessment instruments including medical history and symptom rating scales as well as a physical examination. ME/CFS cases were excluded if they met any exclusion criteria from the 1994 CDC Fukuda and/or Canadian consensus criteria for ME/CFS such as having chronic infections, rheumatic and chronic inflammatory diseases, neurological disorders, psychiatric conditions, or were taking any immunomodulatory medication. Normal controls underwent the same screening process as ME/CFS subjects and were excluded if they reported ME/CFS or other conditions deemed by the recruiting physician to be inconsistent with a healthy control population. Potential normal controls were also excluded if they had a history of substance abuse, psychiatric illness, antibiotics in the prior three months, immunomodulatory medications in the prior year, and clinically significant findings on physical exam or screening laboratory tests [18]. Self-reported IBS was not part of the exclusion criteria of controls. Full selection criteria for both ME/CFS cases and healthy controls can be found in S1 Appendix.

Every participant provided informed written consent in accordance with protocols of the study. The study was approved by the Institutional Review Board at Columbia University Irving Medical Center.

### Plasma collection

Blood samples were collected into BD VacutainerTM Cell Preparation Tubes (CPT) with sodium citrate anticoagulant between June and October 2014, and centrifuged to pellet red blood cells. The plasma samples were shipped to Columbia University at 4°C. After aliquoting, samples were stored at -80°C until they were thawed for proteomic analyses. All samples were analyzed within four years of collection.

### Clinical assessments

Clinical symptoms and baseline health status were assessed on the day of physical examination and biological sample collection from both cases and control subjects using the following surveys: the Short Form 36 Health Survey (SF-36), the Multidimensional Fatigue Inventory (MFI), DePaul Symptom Questionnaire (DSQ) [19] and Pittsburgh Sleep Quality Index (PSQI) [20]. The SF-36 includes the following subject-reported evaluations about current health status: physical and social functioning, physical and emotional limitations, vitality, pain, general health perceptions, and mental health change [21]. The MFI comprises a 20-item self-reported questionnaire focused on general, physical and mental fatigue, activity, and motivation [22]. Cognitive function was tested based on the DSQ questionnaire data and was scored using a standard cognitive disturbance definition as well as a modified definition based on a subset of questionnaire variables. Sleeping disturbances linked to ME/CFS were tested and scored based on DSQ and PSQI questionnaire items. Each instrument was transformed into a 0–100 scale to facilitate combination and comparison, wherein a score of 100 is equivalent to maximum disability or severity and a score of zero is equivalent to no disability or disturbance.

Self-reported IBS was determined based on answers from the medical history form. Subjects were asked if they had received a previous IBS diagnosis or had a history of IBS. Those that answered “YES” were considered positive. An IBS diagnosis was not made at the time of recruitment. 24 of the 50 ME/CFS patients (48%) and one of the 50 control subjects (2%) reported sr-IBS.

### Proteomics analysis

Denaturation/reduction of the sample was performed in 8 M urea/10 mM dithiothreitol/50 mM NH4HCO3 (pH 8.0) for over 60 minutes at 52°C. The solution was stored at room temperature in 20 mM iodoacetamide at the dark for 60 minutes. The urea was diluted to a concentration of 1 M with 50 mM NH_4_HCO_3_ and then digested with trypsin (1:50 ratio) at 37°C with shaking for 16 hrs. After tryptic digestion, the peptide mixture was desalted with C18 micro spin column (C18, Harvard Apparatus, Holliston, MA). The column was washed with 200 μL of 100% acetonitrile and equilibrated with 200 μL of loading buffer (0.1% formic acid). Peptides were loaded onto the column, washed with a loading buffer, and eluted with 200 μL of 70% acetonitrile/ 0.1% formic acid. All steps for loading, washing, and elution were carried out with benchtop centrifugation (300 x g for 2 minutes). The fraction was collected with a 1.5 mL tube and dried on a speed-vac.

2 μg peptides were taken from each sample and mixed with appropriate iRT standard peptides HRM Calibration kit, Biognosys, Schlieren, Switzerland). Desalted peptides were analyzed on a Q-Exactive HF (Thermo Scientific) attached to an Ultimate 300 nano UPLC system (Thermo Scientific). The column (30 cm × 75 μm) was packed in-house with Reprosil 3 μm, 100 Å pore size C18 beads (Maisch GmbH HPLC). Peptides were eluted with a 50 min gradient from 2% to 32% ACN (50min) and to 90% ACN over 5 min in 0.1% formic acid. The method consisted of a full MS1 scan at a resolution of 120 K from m/z 400 to m/z 1000, with AGC set to 1E6 (maximum injection time of 60 ms), followed by 15 m/z windows acquired at a resolution of 30K with AGC set to 1e6 (maximum injection time of  ms); HCD: MS2 activation (collision energy: 27).

All the DIA data were also analyzed by Spectronaut Pulsar X (Biognosys AG, Switzerland, version: 13.10.191212.43655) against the human plasma spectra library. Calibration was set to nonlinear iRT calibration with precision iRT enabled. Identification was performed using a 0.01 q-value cutoff at both the precursor and protein level. The 0.01 q-value corresponds to a false discovery rate of 1% or an estimated 1% of false identifications amongst the accepted identifications. For quantification, the interference correction function was enabled, and the top 3 peptide precursors were summed for protein quantification. We measured relative protein abundance comprehensively, and the effect of the variability potentially generated by the sample preparation and the liquid chromatography–mass spectrometry (LC-MS) performance was minimized using the Local Regression Normalization algorithm described by Callister et al. (2006) [23]. Filtered values may be due to protein levels below the detection threshold of the instrument, or to exclusion from the analysis because of a peptide or protein identification confidence q-value higher than 0.01.

Samples from 50 ME/CFS cases and 50 controls were run in two batches of 20 samples (9 ME/CFS cases, 11 controls) and 80 samples (39 ME/CFS cases, 41 controls). The 20 samples in the first batch were randomly selected. The cases and controls were frequency-matched on the same matching variables as the total study population. 3444 unique peptides matched 257 annotated proteins in the 20 subject sample set. 5308 unique peptides that matched to 279 annotated proteins in the 80 subject sample set. 207 annotated proteins were found in both sample sets. Differences in the number of proteins identified between the two groups may reflect patient heterogeneity or batch effects, particularly amongst low abundance proteins.

### Statistical analyses

For each protein analyte, non-detectable values were replaced with 50% of its smallest available value. Protein levels were then log-transformed with base 10 and rescaled by the control standard deviation.

To test the ME/CFS association and to assess the predictive capacity of each individual protein analyte, we used logistic regression models adjusted for body mass index (BMI), sr-IBS, antidepressant medication use, and all matching variables (age, sex, race/ethnicity, geographic/clinical site, and season of sampling). Prentice and Pyke (1979) [24] showed that the odds ratio estimators and their asymptotic variance matrices can be obtained by applying the logistic regression model to the case-control study as if the data had been obtained in a prospective study. Two separate models were fitted: one with only the linear term (the transformed values) of the protein levels, and one with both linear and quadratic terms (square of the transformed values) of the protein levels as independent variables. Likelihood-ratio tests were used to compare the goodness-of-fit between the two nested models. These were gate-keeping hypothesis tests that would serve to allow further exploration of specific protein compounds. We used the Hochberg step-up procedure [25] to correct for the multiple tests over the annotated proteins controlling the family-wise error rate (FWER) at the level of 0.05. If, for any protein analyte, the model with quadratic term fit the data significantly better than the model with only the linear term through the step-up procedure, adjusted odds ratios (aORs), together with their 95% confidence intervals (95% CI), were calculated comparing ME/CFS risk of various protein levels to that of the reference level at which the ME/CFS risk was at the lowest.

In earlier work with this cohort, sr-IBS was identified as the strongest driving factor in the separation of topological networks based on fecal microbiome and plasma metabolic pathways through an unsupervised and data-driven algorithm (Ayasdi, Menlo Park, California) in Nagy-Szakal et al [9, 26]. It was in this context that we tested, in a stratified analysis, the hypothesis that ME/CFS patients with sr-IBS have altered proteomic profiles.

To examine the utility of the proteomics assay as a biomarker tool for ME/CFS, we applied three machine learning algorithms: Lasso (least absolute shrinkage and selection operator) [27], Random Forests [28] and XGboost [29]. We fitted the linear terms of the levels of all protein analytes, excluding the ones with undetectable/filtered values in more than 50% of the subjects (29 proteins), as predictors in the three classifiers. We excluded these protein analytes in this analysis since they do not have enough power to serve as potential biomarkers. We then measured the importance for each predictor in the classifiers. Lasso regularizes the least squares by adding a penalty term in which the L1 norm of the parameter vector is no greater than a given value, and increasing the penalty drives more coefficients of unimportant predictors to absolute zero. Therefore, measure of importance can be represented as the number of iterations in which the predictor’s parameter estimate in the best fitting model is nonzero. Random Forests measures the mean decrease in accuracy when values of the predictor are randomly permuted. For unimportant predictors, the permutation should have little to no effect on model accuracy, while permuting values of important predictors should significantly decrease it. For XGBoost, a measure of ‘gain’ can be calculated to indicate the relative contribution of the corresponding predictor to the model calculated by taking each predictor’s contribution for each tree in the model. We then selected the protein analytes that were ranked in the top 20 in all three importance measurements and fitted them in the classifiers again, except that here we used the logistic regression instead of Lasso. Since only the linear terms of the protein levels were fitted as predictors, these classifiers did not identify biomarkers associated with ME/CFS with quadratic effects. The predictive performance was evaluated in random resampling cross-validation (CV) with 1000 iterations. In each iteration, the sample set was randomly divided into an 80% training set and a 20% test set. We generated Receiver Operating Characteristic (ROC) curves by evaluating model sensitivity and specificity at different probability thresholds, and then calculated the Area under the Receiver Operating Characteristic curve (AUROC), a performance measure that ranges from 0.5 (fair coin toss) to 1 (perfect prediction).

Data analyses were conducted using Matlab (R2013a, The Mathworks Inc., MA) and R (version 3.5.1). All p-values were 2-tailed.

## Results

### Study population

The study included plasma samples from 50 ME/CFS cases and 50 healthy controls recruited at four sites across the United States. The subset of 39 ME/CFS cases and 41 controls were used in the data analysis to eliminate potential batch effects. The robustness of findings was verified through sensitivity analysis in the total study population with the adjustment of the batches. Characteristics of these two nested sample sets are demonstrated in Table 1.

**Table 1:** Characteristics of the study cohort.

Demographics		ME/CFS (n = 50)	Control (n = 50)	ME/CFS (n = 39)	Control (n = 41)
**Sex**	Female	41	41	30	32
	Male	9	9	9	9
**Age**	Mean (SEM)	51.08 (11.19)	51.32 (11.46)	52.06 (10.87)	51.43 (11.89)
**Race**	White	49	48	39	39
	Asian	1	1	1	1
	Other	0	1	0	1
**Ethnicity**	Not Hispanic or Latino	46	45	37	37
	Hispanic or Latino	4	5	2	4
**Site**	Miami, FL	10	9	6	7
	New York, NY	14	14	12	12
	Salt Lake City, UT	14	15	11	12
	Sierra, NV	12	12	10	10
**Season**	Summer	27	26	22	22
	Fall	23	24	17	19
**sr-IBS**	Yes	24	1	18	1
	No	26	49	21	40
**BMI**	Overweight (>25)	28	22	24	18
	Normal (< 25)	22	28	15	23
**Disease Duration**	< 3 years	4	N/A	2	N/A
	>3 years	46	N/A	37	N/A
**SF-36 Mean (SD)**	Physical Functioning	40.5 (26.71)	96.1 (6.95)	40.13 (27.08)	95.85 (7.41)
	Physical Limitations	8 (22.27)	97 (14.85)	9.62 (24.75)	98.78 (5.45)
	Emotional Limitations	53.33 (47.62)	96 (15.99)	54.7 (48.66)	98.37 (7.27)
	Energy/Fatigue	15.6 (17.46)	74.77 (15.58)	16.03 (18.25)	73.25 (14.43)
	Emotional Well-being	63.44 (19.94)	81.6 (15.25)	65.44 (20.05)	81.27 (11.95)
	Social Functioning	32.75 (25.24)	93.25 (13.88)	32.37 (26.39)	93.6 (13.15)
	Pain	46 (27.17)	91.8 (10.06)	44.04 (28.75)	90.79 (10.3)
	General Health	26.38 (15.33)	83.35 (13.88)	26.63 (16.96)	82.26 (14.07)

The demographics and characteristics of the whole study cohort (n = 100) as well as the subset used for the analysis (n = 80) are shown. ME/CFS: myalgic encephalomyelitis/chronic fatigue syndrome, sr-IBS: self- reported irritable bowel syndrome, BMI: body mass index, SEM: standard error of mean, SF-36: Short form 36 health survey; score on 0–100 scale with 0 = poor and 100 = excellent.

### ME/CFS and ME/CFS subgroups are associated with an altered proteomic profile

S1 Table shows the sample mean and the standard error of the mean (SEM) of levels of each protein within ME/CFS cases, ME/CFS cases with sr-IBS, ME/CFS cases without sr-IBS and controls. S2 Table shows associations between levels of individual protein analytes and ME/CFS outcomes and includes aORs, 95% CIs and corresponding p-values from the logistic regression model in which only the linear term of the protein levels was fitted as an independent variable. The p-values of the likelihood ratio tests that compare the goodness-of-fit of the model with both linear and quadratic terms of the protein levels to that of the model with only the linear term are also presented in S2 Table.

**Fig 1:**
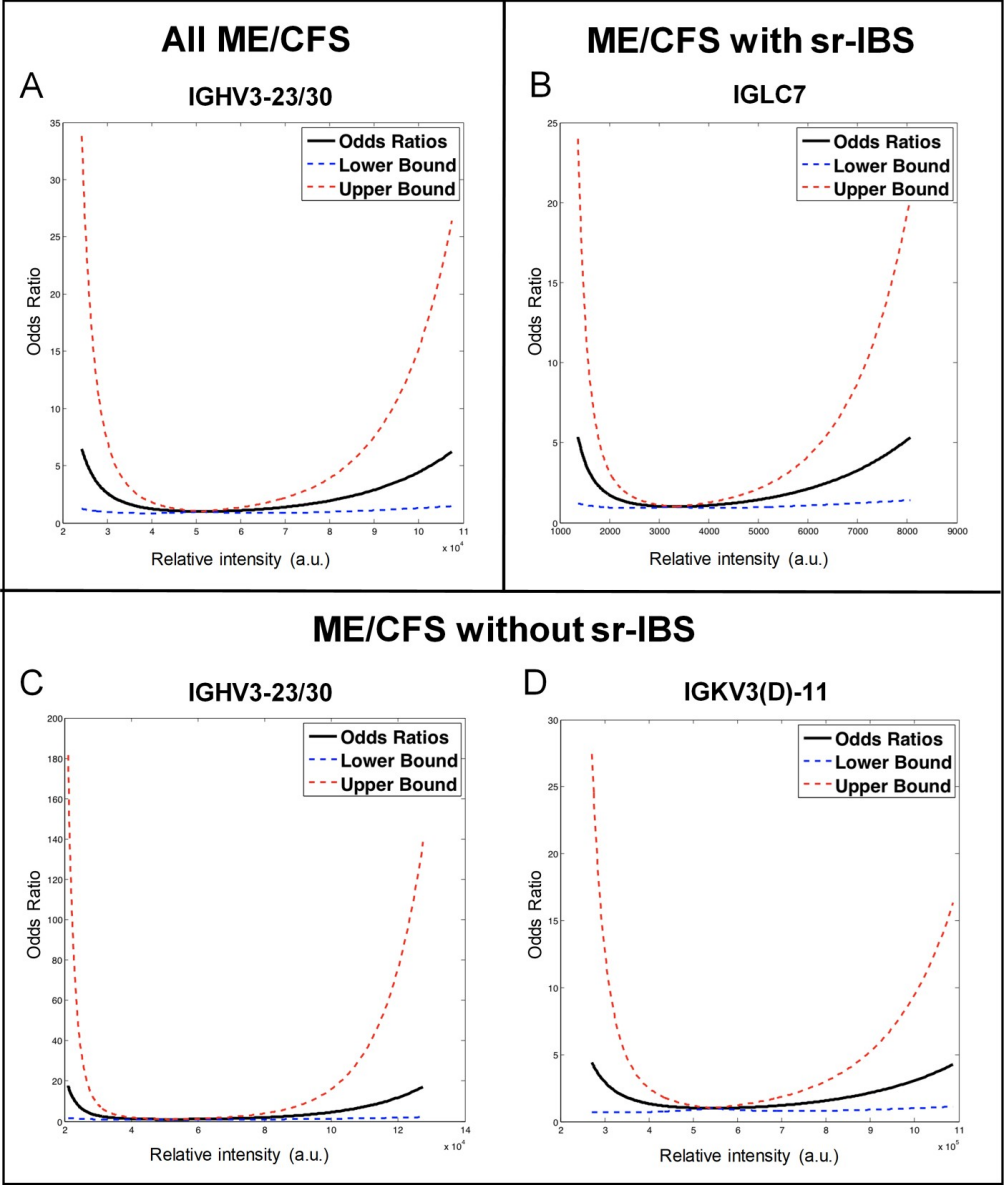
Quadratic effect of immunoglobulin proteins with ME/CFS and ME/CFS subgroups. Two separate models were fitted: one with only the linear term of the protein levels, and one with both linear and quadratic terms of the protein levels as independent variables. In both models we adjusted for BMI, sr-IBS, antidepressant medication use, age, sex, race/ethnicity, geographic/clinical site and season of sampling. Likelihood-ratio tests were used to compare the goodness-of-fit between the two nested models. The Hochberg step-up procedure was applied to correct for the multiple tests over the annotated proteins, controlling the family-wise error rate (FWER) at the level of 0.05. For the protein analytes associated with ME/CFS with significant quadratic effect, adjusted odds ratios (aORs), together with their 95% confidence intervals (95% CI), were calculated comparing ME/CFS risk of various protein levels to that of the reference level at which the ME/CFS risk was at the lowest. (A) All ME/CFS cases versus controls, (B) ME/CFS cases with sr-IBS versus controls, (C & D) ME/CFS cases without sr-IBS versus controls. ME/CFS: myalgic encephalomyelitis/chronic fatigue syndrome, a.u.: arbitrary units, sr-IBS: self-reported irritable bowel syndrome, IGHV: immunoglobulin heavy variable, IGLC: immunoglobulin lambda constant, IGKV: immunoglobulin kappa variable.

Models with only the linear term (transformed values of the protein levels) failed to identify any protein significantly associated with ME/CFS after the FWER correction with the step-up procedure. For certain protein analytes, the residual plots of the linear logistic regression models followed a U-shape pattern (S1 Fig). Therefore, we applied the logistic regression models that included both the linear and the quadratic terms of each protein analyte as independent variables. For immunoglobulin heavy variable (IGHV) 3-23/30, the model with both linear and quadratic terms (square of the transformed values of the protein levels) of the protein levels fit the data significantly better than the model with only its linear term (χ^2^ = 17.57, df = 1, p-value = 2.77E-5). We use the term IGHV3-23/30 because our assay did not distinguish between IGHV3-23 and IGHV3-30. The multiple comparisons of the likelihood-ratio tests over all annotated protein analytes were adjusted using the FWER correction. For IGHV3-23/30, we rejected the null hypothesis of a linear logistic relation between ME/CFS and the analyte in favor of a quadratic relation. Conducting this preliminary gate-keeping test of the quadratic term ensured that the overall type I (false positive) error was controlled at the level of 0.05; accordingly, further examination of this protein analyte using the model with the quadratic term of its levels was justified under the closed-testing principle. The quadratic term can be viewed as an interaction term, hence the aOR depends on the protein level used as a reference. Fig 1A displays, for all ME/CFS subjects versus controls, the plot of aORs with 95% CI for the reference level at 51,000 based on the fitted logistic model with quadratic term. Specifically (Table 2), increasing the protein levels from 51,000 to 100,000 was associated with an increased risk of ME/CFS with an aOR of 4.44 (95% CI: 1.29–15.29, p-value = 0.018), while decreasing the protein level from 51,000 to 25,000 yielded an aOR of 5.65 (95% CI: 1.18–27.04, p-value = 0.030). In a sensitivity analysis that also included the 20-sample test set, the model with quadratic term continued to out-perform the model with only the linear term (χ^2^ = 14.58, df = 1, p-value = 1.34E-4).

**Table 2:** Quadratic relationship of immunoglobulin proteins with ME/CFS and ME/CFS subgroups.

Group	Protein	Reference Level	Comparison	aOR	95% CI	Protein	p-value
**All ME/CFS**	IGHV3-23/30	51,000	decreased to 25,000	5.646	1.179	27.035	0.0303
			increased to 100,000	4.439	1.289	15.286	0.0182
**ME/CFS with sr-IBS**	IGLC7	3,326	decreased to 1,500	3.851	1.115	13.303	0.033
			increased to 7,000	3.257	1.216	8.722	0.019
**ME/CFS without sr-IBS**	IGKV3(D)-11	544,370	decreased to 171,000	59.492	1.062	3332.100	0.047
			increased to 1,100,000	4.527	1.138	18.001	0.032
	IGHV3-23/30	51,000	decreased to 25,000	6.582	1.244	34.816	0.027
			increased to 100,000	4.545	1.284	16.086	0.019

Reference levels based on relative intensity are shown for each protein as well as the aOR, 95% CI and p-value when increasing and decreasing from this point for all ME/CFS patients, ME/CFS patients with sr-IBS and ME/CFS patients without sr-IBS, when compared to the control group. ME/CFS: myalgic encephalomyelitis/chronic fatigue syndrome, sr-IBS: self-reported irritable bowel syndrome, BMI: body mass index, IGHV: immunoglobulin heavy variable; IGLC: immunoglobulin lambda constant; IGKV: immunoglobulin kappa variable, aOR: adjusted odds ratio, CI: confidence interval.

A quadratic effect was also found in female subjects, comprising 30 ME/CFS subjects and 32 controls in our 80-subject sample set (S3 Table). IGHV 3-23/30 was associated with ME/CFS with a significant quadratic effect (χ^2^ = 16.27, df = 1, p-value = 5.50E-5) after controlling the FWER at the level of 0.05. We did not have sufficient statistical power in male subjects (9 ME/CFS patients and 9 controls) for a similar analysis.

Previous work on the same cohort of patients showed that sr-IBS was one of the strongest drivers of separation among ME/CFS cases in terms of both the fecal microbiome and the plasma metabolome [9, 26]. For this reason, we split the ME/CFS group into two subgroups based on their sr-IBS status, and conducted the same analysis using the binary outcome of sr-IBS subgroups vs. controls (S4 Table). Subjects were considered to have sr-IBS if they had reported a previous IBS diagnosis on the medical history form at the time of recruitment.

#### ME/CFS cases with sr-IBS versus controls

When we compared ME/CFS cases with sr-IBS to controls, the immunoglobulin lambda constant (IGLC) 7 had a significant quadratic effect (Fig 1B) on its association with the outcome (χ^2^ = 19.44, df = 1, p-value = 1.04E-5). A protein level of 7,000 resulted in an aOR of 3.26 (95% CI: 1.22–8.72, p-value = 0.019) compared to a reference level of 3,326 and decreasing the protein levels from the reference level to 1,500 yielded an aOR of 3.85 (95% CI: 1.12–13.30, p-value = 0.033).

#### ME/CFS cases without sr-IBS versus controls

When we compared ME/CFS patients without sr-IBS to controls, we found disturbances with quadratic effects in immunoglobulin kappa variable (IGKV) 3(D)-11 (χ^2^ = 14.87, df = 1, p-value = 1.15E-4) and immunoglobulin heavy variable (IGHV) 3-23/30 (χ^2^ = 17.31, df = 1, p-value = 3.18E-5) (Fig 1C and 1D). We use the term IGKV3(D)-11 because our assay did not distinguish between IGKV3-11 and IGKV3(D)-11. Increasing the levels of IGKV3(D)-11 from the reference level of 544,370 to 1,100,000 yielded an aOR of 4.53 (95% CI: 1.14–18.00, p-value = 0.032), while decreasing from the reference level to 171,000 resulted in an aOR of 59.49 (95% CI: 1.06–3,332.10, p-value = 0.047). For IGHV3-23/30, altering its levels from 51,000 to 100,000 and from 51,000 to 25,000 resulted in aORs of 4.54 (95% CI: 1.28–16.09, p-value = 0.019) and 6.58 (95% CI: 1.24–34.82, p-value = 0.027), respectively.

### Assessment of the proteomics assay as a potential diagnostic tool for ME/CFS

**Fig 2:**
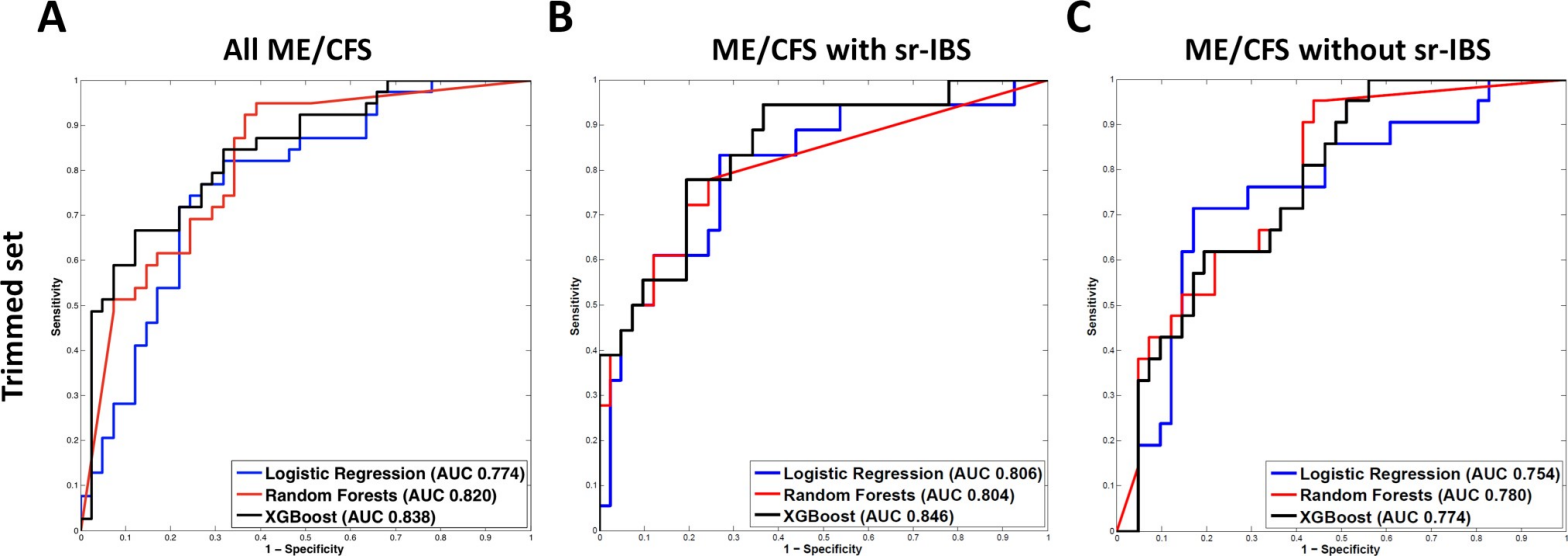
Diagnostic performance (AUROC) of ME/CFS and ME/CFS subgroup plasma proteomes. Three machine learning algorithms were used to examine the utility of the proteomics assay as a biomarker tool for ME/CFS: Lasso (least absolute shrinkage and selection operator), Random Forests, and XGboost. We fitted all protein analytes, excluding the ones with more than 50% undetectable/filtered values, as predictors in the three classifiers and measured the importance for each predictor in the classifiers. The protein analytes that were ranked in the top 20 in all three importance measurements were fitted in the classifiers again (Trimmed set), except that here we used the logistic regression model instead of Lasso. The predictive performance was evaluated in random resampling cross-validation (CV) with 1,000 iterations from which we calculated the Area under the Receiver Operating Characteristic curve (AUROC) values and generated Receiver Operating Characteristic (ROC) curves for (A) all ME/CFS cases, (B) ME/CFS cases with sr-IBS and (C) ME/CFS cases without sr-IBS. ME/CFS: myalgic encephalomyelitis/chronic fatigue syndrome, sr-IBS: self-reported irritable bowel syndrome.

After excluding the proteins with more than 50% undetectable/filtered values, we fitted the remaining 250 protein analytes as predictors in three different classifiers: Lasso, Random Forests, and XGBoost. Protein analytes that were ranked in top 20 in importance measurements in all three classifiers (Table 3) were fitted as predictors in the same classifiers again, except that here we used the logistic regression model instead of Lasso. Fig 2A, Fig 2B and Fig 2C show the ROC curves and the AUROC values from these three classifiers with the trimmed set of protein analytes as predictors differentiating all ME/CFS, ME/CFS with sr-IBS, and ME/CFS without sr-IBS, respectively, from the controls. All of them performed significantly better than a fair coin toss (S5 Table). Notably, XGBoost with six protein analytes (Table 3) distinguished ME/CFS with sr-IBS from the controls with a high degree of accuracy with a cross-validated AUROC value of 0.846 (95% CI: 0.703–0.927).

**Table 3:** Potential plasma protein biomarkers for ME/CFS.

Gene Name	Uniprot ID	Direction	Lasso		Random Forest		XGBoost	
			Percentage^1^	Rank^4^	Mean Decrease in accuracy^2^	Rank^4^	Gain^3^	Rank^4^
**All ME/CFS**								
**CAMP**	P49913	Increased	22.80%	1	0.1284	4	0.0652	2
**LRG1**	P02750	Decreased	9.90%	9	0.1302	3	0.0327	4
**IGF1**	P05019	Decreased	3.90%	19	0.1320	2	0.0318	6
**GSN**	P06396	Decreased	3.70%	20	0.0743	9	0.0281	8
**IGFALS**	P35858	Decreased	11.60%	7	0.0988	7	0.0292	7
**IGLV1-47**	P01700	Decreased	14.10%	2	0.0639	14	0.0319	5
**FCRL3**	Q96P31	Decreased	4.70%	17	0.0545	20	0.0127	17
**CRTAC1**	Q9NQ79	Decreased	13.30%	3	0.2653	1	0.1225	1
**ME/CFS with sr-IBS**								
**CAMP**	P49913	Increased	30.10%	1	0.1772	2	0.0852	2
**SERPINA3**	P01011	Decreased	4.20%	16	0.0731	7	0.0249	6
**IGF1**	P05019	Decreased	11.00%	6	0.1768	3	0.1132	1
**ITIH2**	P19823	Decreased	13.60%	4	0.1870	1	0.0529	4
**IGHV1-18**	A0A0C4DH31	Decreased	19.30%	3	0.0535	17	0.0157	14
**CRTAC1**	Q9NQ79	Decreased	4.80%	13	0.0922	4	0.0577	3
**ME/CFS without sr-IBS**								
**PON3**	Q15166	Increased	7.20%	3	0.0601	19	0.0571	2
**KNG1**	P01042	Increased	3.70%	13	0.0674	17	0.0122	20
**LRG1**	P02750	Decreased	5.50%	6	0.0960	8	0.0400	4
**IGLC7**	A0M8Q6	Decreased	8.40%	2	0.0664	18	0.0196	14
**CRTAC1**	Q9NQ79	Decreased	3.90%	12	0.1031	6	0.0740	1

Proteins with more than 50% undetectable/filtere values were excluded. All 250 protein analytes were fitted as predictors in 3 different classifiers: Lasso, Random Forests, and XGBoost. Table shows the proteins that were ranked in the top 20 of importance measurements for all ME/CFS patients, ME/CFS patients with sr-IBS and ME/CFS patients without sr-IBS. Direction is measured relative to controls. ME/CFS: myalgic encephalomyelitis/chronic fatigue syndrome, sr-IBS: self-reported irritable bowel syndrome, CAMP: cathelicidin antimicrobial protein, LRG1: Leucin-rich glycoprotein 1, IGF1: insulin-like growth factor 1, IGFALS: Insulin-like growth factor-binding protein complex acid labile subunit, IGLV1-47: immunoglobulin lambda variable region 1–47, FCRL3: Fc receptor-like protein 3, SERPINA3: Alpha-1-antichymotrypsin, ITIH2: Inter-alpha-trypsin inhibitor heavy chain H2, IGHV1-18: immunoglobulin heavy variable region 1–18, PON3: Serum paraoxonase/lactonase 3, KNG1: Kininogen 1, IGLC7: immunoglobulin lambda constant region 7.

^1^Percentage: Lasso regularizes the least squares by adding a penalty term in which the L1 norm of the parameter vector is no greater than a given value, and increasing the penalty drives more coefficients of unimportant predictors to absolute zero. Therefore, measure of importance can be represented as the percentage of iterations (out of 1,000 random resampling cross-validation iterations) in which the predictor’s parameter estimate in the best fitting model is nonzero.

^2^Mean Decrease in Accuracy: Random Forests measures the mean decrease in accuracy when values of the predictor are randomly permuted. For unimportant predictors, the permutation should have little to no effect on model accuracy, while permuting values of important predictors should significantly decrease it.

^3^Gain: XGBoost measures the importance of predictors in ‘Gain’ to indicate the relative contribution of the corresponding predictor to the model calculated by taking each predictor’s contribution for each tree in the model.

^4^Rank: We selected the protein analytes that were ranked in the top 20 in all three importance measurements.

## Discussion

We pursued proteomic analyses of plasma from subjects with ME/CFS with the objectives of obtaining insight into the pathogenesis of ME/CFS and finding biomarkers for the disease. Using models with only linear terms, we did not identify significant differences between cases and controls following FWER correction. However, using a model that included both linear and quadratic terms we found a significant association between ME/CFS and IGHV3-23/30. The association with quadratic effect revealed both a positive and negative correlation relationship between levels of IGHV3-23/30 and ME/CFS. Whilst 12 patients had extremely high levels (>100,000) of IGHV3-23/30, only 3 patients had extremely low levels (<25,000) of this protein. IGHV3-23 is one of the most commonly used heavy variable regions in the human immunoglobulin (Ig) repertoire [30]. Its usage has been linked with non-Hodgkin lymphomas (NHL) such as chronic lymphoid leukemia (CLL) [31–34], mantle cell lymphoma (MCL) [35], splenic marginal zone lymphoma (MZL) [36, 37], Waldenström’s macroglobulinemia [38], and follicular lymphoma (FL) [39, 40]. Disease progression in these B-cell malignancies is driven by chronic stimulation from either microbial or auto-antigens. Interruption of B-cell signaling through use of kinase inhibitors has been shown to have therapeutic benefit in some patients with MZL, CLL, FL, and MCL [41, 42]. Increased IGHV3-23 usage is also reported in anti-myelin associated glycoprotein neuropathy and in monoclonal gammopathy of undetermined significance [43], a disorder that may progress to malignant lymphoproliferative disease [44]. We speculate that, at least in a subset of ME/CFS subjects, an increased level of IGHV3-23 may be due to antigen driven clonal expansion, and that these patients might benefit from identification of the antigen driving B-cell receptor signaling or kinase inhibitors that interrupt signaling. In this context, we also note that ME/CFS has been linked with an increased risk of developing MZL and other NHL [45].

Due to the high prevalence of sr-IBS in ME/CFS patients and to the fact that previous studies showed it was one of the strongest drivers in the separation of fecal metagenomics and plasma metabolomics within the patient group [9, 26], we further stratified the ME/CFS group based on sr-IBS status. Similar to all ME/CFS cases, IGHV3-23 was associated with ME/CFS without sr-IBS with a quadratic effect. In addition, IGKV3(D)-11 was also significantly associated with ME/CFS without sr-IBS. This protein showed the same pattern in ME/CFS subjects as a whole, but did not reach significance. IGKV3-11, like IGHV3-23, is one of the most commonly used light chain variable regions. Anti-hemagglutinin antibodies following influenza infection predominantly represent this light chain variable region [46]. IGKV3-11 is repeatedly paired with IGHV3-30 in protective antibodies to CMV [47, 48] as well as the 23F polysaccharide of *Streptococcus pneumoniae* [49–51]. However, since mass spectrometry does not provide us with information in terms of heavy/light chain pairings, it is unclear whether this is relevant in our cohort. ME/CFS patients with sr-IBS, on the other hand, presented with a different proteomics profile. Only IGLC7 was significantly associated with disease in ME/CFS patients with sr-IBS. Unlike the variable region which is involved in antigen recognition, the constant region is thought to have more of a regulatory role, although it may have an impact on the variable region’s structure and function [52]. The link between sr-IBS and this specific lambda constant region is currently unclear. However, our results show that, as was found with metabolites and the microbiome [9, 26], sr-IBS also influences the proteomic profile. B cell dysregulation has previously been reported in ME/CFS [53]. Bradley et al. (2013) [54] found greater numbers of naïve and transitional B cells in patients whilst Brenu et al. (2014) [55] found increased numbers of memory B cells. Guenther et al (2015) [56] found a decrease in IgG3 and 4, but an increase in IgG2 and IgM. We do not see any overall changes to Ig isotypes. Although ME/CFS patients with sr-IBS had decreased IgG1 (aOR = 0.361, p-value = 0.037) and increased IGHA2 (aOR = 2.852, p-value = 0.046) levels, these findings were not significant after adjustment for multiple testing.

We also employed a data-driven approach to identify plasma protein signatures to assess their utility as biomarkers of ME/CFS. Using the top 20 ranked proteins identified by Lasso, Random Forests, and XGBoost, we found a panel of eight plasma proteins that yielded high AUC values of up to 0.838. These proteins were cathelicidin antimicrobial peptide (CAMP), Ig lambda variable region 1–47 (IGLV1-47), Fc receptor-like protein 3 (FCRL3), leucin-rich glycoprotein 1 (LRG1), gelsolin (GSN), cartilage acidic protein 1 (CRTAC1), insulin-like growth factor 1 (IGF1), and IGF-binding protein acid labile subunit (IGFALS). IGHV3-23/30 was not identified as a top-ranked biomarker because it is associated with ME/CFS only with a quadratic effect. While Random Forests and XGBoost are both tree models and can potentially reveal non-linear relationships between predictors and the outcome, Lasso is parametric and cannot identify quadratic relationships if only the linear terms of protein analytes were used as predictors. Since we selected the panel of protein analytes that were highly ranked in all three classifiers, the proteins that were significantly associated with ME/CFS quadratically would be filtered out by this strategy. We are not aware of other serum or plasma proteomic studies that led to proposed biomarker sets. However, Baraniuk et al (2005) [11] used a logistic model to predict ME/CFS status based on the cerebrospinal fluid proteome that predicted ME/CFS status with 80% accuracy based on alpha-1-macroglobulin, amyloid precursor-like protein 1, keratin 16, orosomucoid 2, and pigment epithelium-derived factor.

CAMP is an antimicrobial peptide that has both pro- and anti-inflammatory effects and direct bactericidal activity, as well as anti-viral and anti-fungal properties [57]. Serum levels are increased during both bacterial and viral infections [58, 59], and in autoimmune diseases such as psoriasis, systemic lupus erythematosus, and rheumatoid arthritis [60]. The increase observed in our cohort is consistent with inflammation caused either by infection or autoimmunity. FCRL3 is a transmembrane protein preferentially expressed on B lymphocytes. It has been shown to inhibit B cell receptor mediated signaling [61] and abrogate plasma cell differentiation and antibody production [62]. IGLV1-47 and FCRL3 are both associated with B cells and are decreased in the ME/CFS patients supporting the idea that there may be dysregulation of the B cell response. LRG1 is a pleiotropic secreted pro-inflammatory glycoprotein whose overexpression has been linked to a number of autoimmune diseases such as rheumatoid arthritis and Crohn disease [63], as well as neurodegenerative diseases such as Parkinson disease [64]. It is expressed during differentiation of granulocytes [65] and is present in neutrophil granules [66]. Abnormalities reported in neutrophil function in ME/CFS patients include increased apoptosis, characterized by increased annexin V binding and increased expression of the death receptor tumor necrosis factor 1 [67], and decreased capacity to produce reactive oxygen species following *Escherichia coli* phagocytosis [68]. Lower LRG1 levels observed in this cohort may reflect neutrophil dysfunction. GSN is a multifunctional protein that has actin binding activities. It is thought to have anti-inflammatory effects and to protect against oxidative stress by scavenging actin released from damaged cells and tissues [69]. Lower serum levels are observed in inflammatory states including acute injury [70], sepsis [71], and rheumatoid arthritis [72]. Decreased plasma levels in ME/CFS patients may reflect a pro-inflammatory environment. IGF1 is an important growth factor that has many roles in growth development and homeostasis. IGF1 decreases in response to pro-inflammatory cytokines, such as tumor necrosis factor-α, interleukin (IL) 1β, and IL-6; low circulating levels of IGF1 are associated with infection, trauma, and aging [73, 74]. IGFALS binds IGF1 in serum thereby increasing its half-life. Levels of both IGFALS and IGF1 are decreased in the plasma of our ME/CFS patients. IGF1 deficiency has previously been linked to ME/CFS [75] although data has been conflicting [76, 77]. Levels of CRTAC1 were also decreased. It is an extracellular matrix protein secreted by chondrocytes [78]. Our data-driven approach identified the protein analytes that were most powerful in predicting ME/CFS status in our cohort. Whilst pathway enrichment analysis on so few proteins failed to identify a significant pathway that encompasses the selected analytes, many of the proteins have a role in immune and inflammatory responses, which have shown to be dysregulated in ME/CFS.

Using Lasso, Random Forests, and XGBoost, we also identified a panel of proteins that predicted both ME/CFS with and without sr-IBS with high accuracy. The predictors for the ME/CFS without sr-IBS included LRG1 and CRTAC1 as well as IGLC7, KNG1, and PON3. Levels of KNG1 were increased in ME/CFS without sr-IBS patients with an aOR of 4.116 (p-value = 0.031). KING1 is an acute phase protein and part of the kallikrein-kinin system, also known as the contact system [79]. Its cleavage product, bradykinin, is an inflammatory mediator leading to vasodilation, increase of vascular permeabilization, smooth muscle contraction, fever, and release of nitric oxide [80]. Increased KNG1 levels may be a sign of increased inflammation. Elevated levels of proteins involved with acute phase signaling have been found in fibromyalgia [81] and increased KNG1 was found associated with pain in women with chronic widespread pain [82]. However, in our study, we did not find a significant association between KNG1 levels and fibromyalgia when we compared ME/CFS patients with fibromyalgia (n = 20) and ME/CFS without fibromyalgia (n = 19) using the Wilcoxon rank-sum test (z-score = 0.126, p-value = 0.899). PON3, like PON1, is thought to be associated with high-density lipoproteins (HDL) and to protect low-density lipoproteins (LDL) from oxidation [83, 84]. Infection and inflammation leads to increased LDL oxidation [85] and higher LDL oxidation has previously been shown to be associated with ME/CFS [76, 86, 87] Increased levels of PON3 may be a mechanism to compensate for increased oxidative stress caused by increased inflammatory mediators in the blood.

Predictors in ME/CFS patients with sr-IBS that were also identified in the whole ME/CFS cohort included IGF1, CRTAC1, and CAMP along with SERPINA3, ITIH2, and IGHV1-18. The presence of immunoglobulin proteins among the predictors is consistent with the involvement of B cells in ME/CFS pathogenesis. SERPINA3 and ITIH2 are protease inhibitors involved in inflammation. Their levels were slightly decreased in our ME/CFS with sr-IBS cohort. Increased serum and cerebrospinal fluid levels of the acute phase protein SERPINA3 are associated with Alzheimer disease [88]; however, it is also thought to limit inflammation by controlling superoxide generation and inhibiting proteases, such as elastase and chymase, with cathepsin G thought to be its primary target [89]. Levels of CAMP were particularly elevated in sr-IBS patients with an aOR = 4.335 (p-value = 0.011). Levels of CAMP are increased in the mucosa of inflammatory bowel disease (IBD) patients [90, 91]. We are not aware of similar studies in sr-IBS. Polymeric immunoglobulin receptor (PIGR) was detected more often in ME/CFS subjects compared to controls (56.4% versus 26.8% respectively, chi squared p-value = 0.007). However, we do not consider PIGR as a biomarker for ME/CFS because it was not detected >50% of subjects. PIGR transports IgA to the lumen at mucosal surfaces. Its expression in the gut is regulated by exposure to bacterial and viral products from both the microbiota and pathogens [92]. We speculate that microbial dysbiosis [26, 93] leads to increased inflammation and increased gut permeability, resulting in higher plasma levels of CAMP and PIGR.

Whilst our exploratory study has identified a plasma protein biosignature using machine-learning algorithms that can predict ME/CFS status adequately, the clinical utility of these results remains to be shown. Validation of the panel of proteins in larger cohorts is needed to determine whether it would make a reliable biomarker. In addition, the specificity of the biosignature would need to be assessed to see if it could successfully distinguish ME/CFS cases, as well as ME/CFS cases with or without sr-IBS, from those with other fatiguing illnesses such as fibromyalgia and Gulf War Illness. Disease-specific specific biomarkers could provide an objective measure to aid in diagnosis of this heterogeneous disease. Previous proteomic studies in cerebrospinal fluid and saliva have identified protein signatures with predictive accuracies comparable to ours [11, 14], however, none have led to the development clinical biomarkers. In fact, no molecular biomarkers have been validated for ME/CFS diagnosis or prognosis [4], highlighting the challenges associated with this complex disease. Our work, whilst exploratory in nature, shows that the plasma proteome is a viable and untapped source of potential biomarkers in ME/CFS, and can provide insight into disease pathophysiology. In addition, we support previous results that ME/CFS patients with sr-IBS may constitute a subgroup with a distinct molecular profile [9, 26] and that considering subtypes of ME/CFS can lead to greater predictive accuracy in biomarker studies.

Our study is limited by small sample size, and the robustness of our findings needs to be verified in larger cohorts. Additionally, IBS status determination and stratification could be improved by an independent diagnosis at the time of participant recruitment. Nonetheless, our results comport with other work in ME/CFS that has found evidence in ME/CFS of immune dysregulation, B cell dysfunction, chronic inflammation, oxidative stress, and autoimmunity.

## Supporting information

S1 Appendix Study inclusion and exclusion criteria for ME/CFS patients and healthy controls. (PDF)

S1 Fig Residual plots of IGKV3D-20 and IGHV3-23/30. Residual plots of the logistic regression model with only the linear term of protein levels as the independent variable for immunoglobulin kappa variable (IGKV) 3D-20 and immunoglobulin heavy variable (IGHV) 3-23/30.
(TIF)

S1 Table Mean levels of proteins detected in all ME/CFS cases, ME/CFS with sr-IBS and ME/CFS without sr-IBS. ME/CFS: myalgic encephalomyelitis/chronic fatigue syndrome, IBS: irritable bowel syndrome, SEM: standard error of mean.
(PDF)

S2 Table Statistical analysis of protein association with ME/CFS. The aOR, 95% confidence levels and p-values from the logistic regression model in which only the linear term of the protein levels was fitted as an independent variable are shown. Quadratic effect p-value corresponds to the likelihood ratio tests comparing the goodness-of-fit of the model with both linear and quadratic terms of the protein levels to the goodness-of-fit of the model with only the linear term. aOR: adjusted odds ratio, CI: confidence interval. ^1^Quadratic effect p-value: crude p-value of the likelihood ratio test comparing the goodness-of-fit between the logistic regression model with both linear and quadratic terms of the protein level and the model with only the linear term. Hochberg step-up procedure was applied to correct for the multiple tests over the annotated proteins controlling the family-wise error rate (FWER) at the level of 0.05.
(PDF)

S3 Table Mean levels of proteins detected in all female ME/CFS cases. aOR, 95% confidence levels and p-values from the logistic regression model in which only the linear term of the protein levels was fitted as an independent variable are shown in females only. Quadratic effect p-value corresponds to the likelihood ratio tests that compare the goodness-of-fit of the model with both linear and quadratic terms of the protein levels to that of the model with only the linear term in females only. ME/CFS: myalgic encephalomyelitis/chronic fatigue syndrome, SEM: standard error of mean, aOR: adjusted odds ratio, CI: confidence interval. ^1^Quadratic effect p-value: crude p-value of the likelihood ratio test comparing the goodness-of-fit between the logistic regression model with both linear and quadratic terms of the protein level and the model with only the linear term. Hochberg step-up procedure was applied to correct for the multiple tests over the annotated proteins controlling the family-wise error rate (FWER) at the level of 0.05.
(PDF)

S4 Table Statistical analysis of individual protein associations with ME/CFS with sr-IBS and ME/CFS without sr-IBS. aOR, 95% confidence levels and p-values from the logistic regression model in which only the linear term of the protein levels was fitted as an independent variable are shown. Quadratic effect p-value corresponds to the likelihood ratio tests that compare the goodness-of-fit of the model with both linear and quadratic terms of the protein levels to that of the model with only the linear term. ME/CFS: myalgic encephalomyelitis/chronic fatigue syndrome, sr-IBS: self-reported irritable bowel syndrome, aOR: adjusted odds ratio, CI: confidence interval. ^1^Quadratic effect p-value: crude p-value of the likelihood ratio test comparing the goodness-of-fit between the logistic regression model with both linear and quadratic terms of the protein level and the model with only the linear term. Hochberg step-up procedure was applied to correct for the multiple tests over the annotated proteins controlling the family-wise error rate (FWER) at the level of 0.05.
(PDF)

S5 Table Assessment of predictive power of the classifiers Lasso/Logistic regression, Random Forests, and XGBoost for all ME/CFS patients, ME/CFS patients with sr-IBS, and ME/CFS patients without sr-IBS, when compared to the control group. ME/CFS: myalgic encephalomyelitis/chronic fatigue syndrome, sr-IBS: self-reported irritable bowel syndrome, AUC: area under the curve, CI: confidence interval.
(PDF)
