# Supplementary material for: Plasma proteomic profiling suggests an association between antigen driven clonal B cell expansion and ME/CFS
Source: PLoS One. 2020 Jul 21;15(7):e0236148. doi: 10.1371/journal.pone.0236148 (PMC7373296; doi:10.1371/journal.pone.0236148)
Supplement: S1 Appendix — (PDF) [file pone.0236148.s001.pdf]

Inclusion criteria for CFS cases

- Patient is between ≥ 18 and ≤ 65 years of age at time of signing of consent.
- Patients with **previously confirmed diagnosis of CFS** as established by the International Chronic Fatigue Syndrome Study Group (Fukuda 1994), **AND/<u>OR</u>** the recently updated Canadian criteria (Jason 2010).

Exclusion Criteria for CFS Cases

3.2.1 Patients do not meet the Fukada Criteria or the Canadian Criteria of CFS for inclusion.

3.2.2 Patients meet any of the exclusion criteria associated with the set of inclusion criteria used to establish their diagnosis of CFS (see Appendix B: Exclusion ME/CFS Clinical Diagnostic Worksheet, Carruthers et al.).

3.2.2.1 Exclusion Criteria for the Fukuda Criteria

- Organ failure including emphysema, cirrhosis, cardiac failure, or chronic renal failure.
- Chronic infections including AIDS, hepatitis B, or hepatitis C.
- Rheumatic and chronic inflammatory diseases including systemic lupus erythematosus, Sjögren's syndrome, rheumatoid arthritis, inflammatory bowel disease, or chronic pancreatitis.
- Major neurological diseases including multiple sclerosis, neuromuscular diseases, stroke, head injury with residual neurologic deficits, or epilepsy,
- Diseases requiring systemic treatment including organ or bone marrow transplantation, chemotherapy, or radiation of brain, thorax, abdomen, or pelvis.
- Major endocrine diseases including hypopituitarism or adrenal insufficiency.
- Primary sleep disorders including untreated sleep apnea or narcolepsy.
- Sleep disorders such as restless leg syndrome and periodic limb movement, if they are severe, but not if the degree of the sleep problem is insufficient to explain the severity of fatigue.
- Fatigue caused by medications, sleep deprivation, untreated hypothyroidism, untreated or unstable diabetes mellitus, or active infection.
- Females who are pregnant, < 3 months postpartum, or currently lactating.

- Major surgery < 6 months after operation or minor surgery < 3 months after operation.
- Major infections such as sepsis or pneumonia <3 months postresolution.
- Myocardial infarction or heart failure < 5 years after event.
- Morbid obesity BMI>40.
- Psychiatric conditions including lifetime diagnosis of bipolar affective disorders, schizophrenia of any subtype, delusional disorder of any subtype, organic brain disorders, or major depressive disorder with psychotic or melancholic features, anorexia nervosa, or bulimia < 5 years before the onset of chronically fatiguing illness

3.2.2.2 Exclusion Criteria for the Canadian Criteria

- Active diseases processes that explain most of the major symptoms of fatigue, sleep disturbance, pain, and cognitive dysfunction including

Addison's disease, Cushing's Syndrome, hypothyroidism, hyperthyroidism, iron deficiency, other treatable forms of anemia, iron overload syndrome, diabetes mellitus, and cancer.

- Untreated sleep disorders such as upper airway resistance syndrome or obstructive or central sleep apnea.
- Rheumatological disorders such as rheumatoid arthritis, lupus, polymyositis and polymyalgia rheumatica.
- Immune disorders such as AIDS.
- Neurological disorders such as multiple sclerosis (MS), Parkinsonism, myasthenia gravis and untreated B12 deficiency
- Infectious diseases such as tuberculosis, chronic hepatitis, acute Lyme disease.
- Primary psychiatric disorders and substance abuse.
- Exclusion of other diagnosis cannot be reasonably excluded by the patient's history and physical examination is achieved by laboratory testing and imaging.

3.2.3 Patients taking immunomodulatory medications and/or medications that cause immunodeficiency or immunosuppression will be excluded. Examples include but are not limited to medications such as: prednisone, cortisone, plaquenil, methotrexate, TNF inhibitors. Limited number of participants on immune enhancing drugs such as Ampligen and Isoprinosone may be included if subject is on stable dosing for more than three months. A list of immunosuppressive drugs by category with examples can be found in the tables "Causes of Secondary Immunodeficiency" and "Some Drugs that Cause Immunosuppression" at this website:http://www.merck.com/mmpe/sec13/ch164/ch164a.html

3.2.4 Patients treated with long-term (longer than 2 weeks) antiviral medication within the past 6 months.

3.2.5 Patients treated with long-term (longer than 2 weeks) antibiotics within the past three months.

3.2.6 Patients treated with short-term (less than 2 weeks) antiviral or antibiotic medication within the past 30 days.

3.2.7 Patients using antiretroviral medication within the past year.

3.2.8 Patients unable to read, understand, or speak English.

3.2.9 Subject with history of substance abuse in the past year (excluding nicotine and caffeine) as determined by patient self-report.

3.2.10 Subject fails clinical laboratory or physical exam screen.

3.2.11 Patients who in the professional opinion of the PI or attending physician, should not be enrolled.

3.3 Inclusion Criteria for Controls

3.3.1 Person is generally healthy and is ≥18 years ≤ 65 years of age.

3.3.2 Regional controls residing for ≥ 1 year within a 100 mile radius of this clinical location, not residing in the same household, related to and not a sexual partner of any participants or CFS person who is not a participant.

3.3.3 Frequency-match CFS cases by age (within 5 years) and sex.

3.3.4 Control has the following laboratory values measured within 6 weeks of On-Study Visit/blood draw:

Values must be within normal limits for institution:

- CBC with Differential
- Comprehensive chemistry panel (SMA 18)
- ESR
- TSH

3.3.5 Negative serology testing for HIV (within past year).

3.3.6 Person is able to read, understand and speak English.

3.3.7 Record of EBV ea and IgM, HHV6, Coxsackie B panel and XMRV status, including laboratory where assessed, if known (testing is not required for participation)

Exclusion Criteria for Controls

3.4.1 Subject meets the clinical criteria for the diagnosis of chronic fatigue syndrome as established by the International Chronic Fatigue Syndrome Study Group (Fukuda 1994) or the revised Canadian criteria (Jason 2010).

3.4.2 Subject has a diagnosis or history of CFS.

3.4.3 Subject with any active or uncontrolled co-morbidities which, according to the investigator or the case definitions referenced in Section 3.4.1, may interfere with the ability of the subject to participate in the study.

3.4.4 Subjects taking immunomodulatory medications and/or medications that cause immunodeficiency or immunosuppression will be excluded. Examples include but are not limited to medications such as: prednisone, cortisone, plaquenil, methotrexate, TNF inhibitors. Immune enhancing drugs such as Ampligen and Isoprinosone may be included if subject is on stable dosing for more than three months. A list of immunosuppressive drugs by category with examples can be found in the tables "Causes of Secondary Immunodeficiency" and "Some Drugs that Cause Immunosuppression" at this website:http://www.merck.com/mmpe/sec13/ch164/ch164a.html
