# Supplementary figures and images for: Plasma proteomic profiling suggests an association between antigen driven clonal B cell expansion and ME/CFS
Source: PLoS One. 2020 Jul 21;15(7):e0236148. doi: 10.1371/journal.pone.0236148 (PMC7373296; doi:10.1371/journal.pone.0236148)

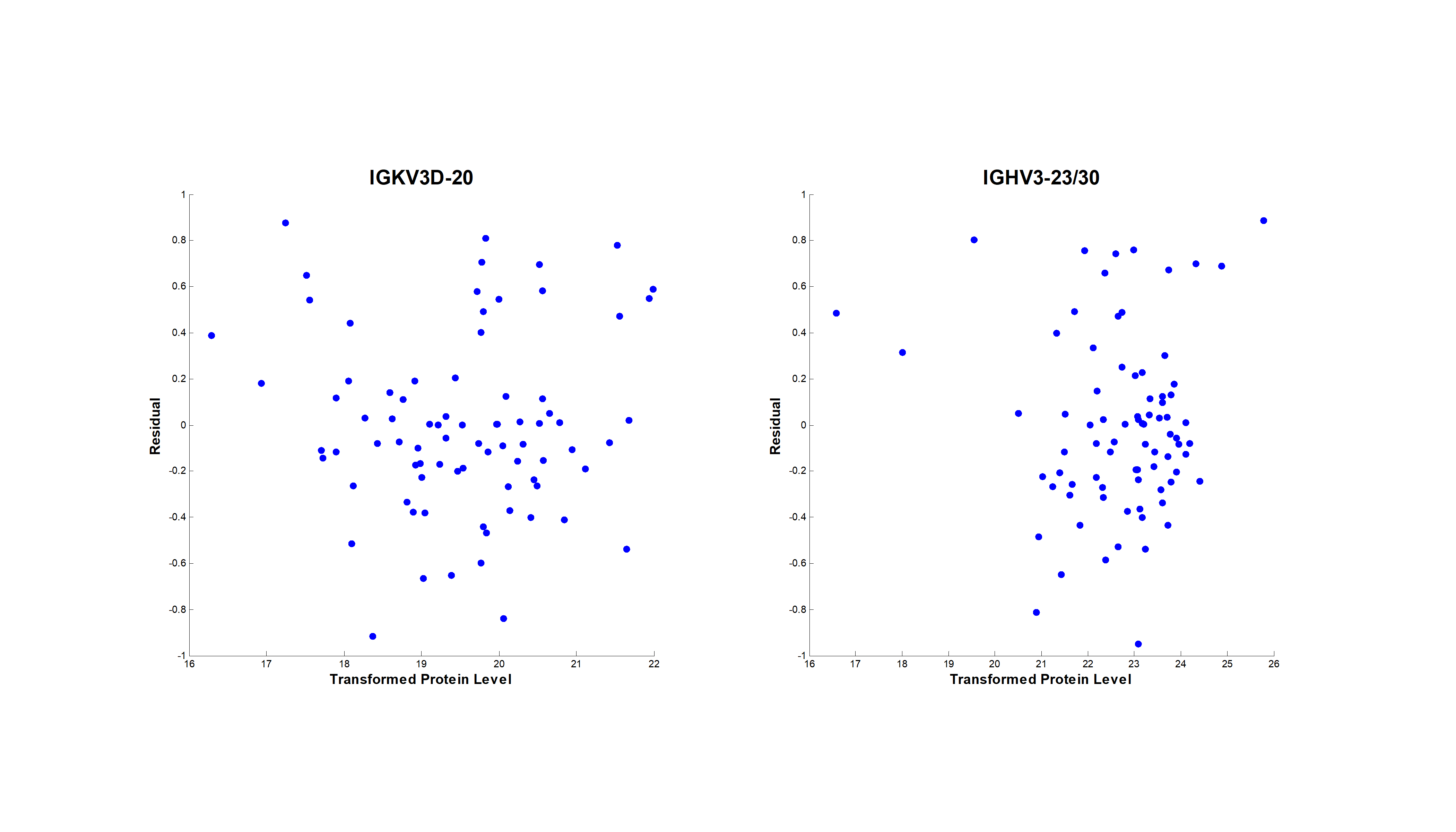

Supplement: S1 Fig — Residual plots of the logistic regression model with only the linear term of protein levels as the independent variable for immunoglobulin kappa variable (IGKV) 3D-20 and immunoglobulin heavy variable (IGHV) 3-23/30. (TIF) [file pone.0236148.s002.tif]
