# Supplementary material for: Plasma proteomic profiling suggests an association between antigen driven clonal B cell expansion and ME/CFS
Source: PLoS One. 2020 Jul 21;15(7):e0236148. doi: 10.1371/journal.pone.0236148 (PMC7373296; doi:10.1371/journal.pone.0236148)
Supplement: S1 Table — ME/CFS: myalgic encephalomyelitis/chronic fatigue syndrome, IBS: irritable bowel syndrome, SEM: standard error of mean. (PDF) [file pone.0236148.s003.pdf]

Supplemental Table 1. Mean levels of proteins detected in all ME/CFS cases, ME/CFS with sr-IBS and ME/CFS without sr-IBS.

Protein Name	Gene name	UniProt ID		All ME/CFS		ME/CFS with sr-IBS		ME/CFS without sr-IBS		Control	
			Undetectable values (%)	Mean	SEM	Mean	SEM	Mean	SEM	Mean	SEM
immunoglobulin lambda variable 4-69	IGLV4-69	A0A075B6H9	30	11982.15	5912.64	15666.98	11992.27	8481.57	3436.06	13541.35	4207.87
immunoglobulin lambda variable 8-61	IGLV8-61	A0A075B6I0	10	29131.30	4224.20	28318.79	6158.07	29903.17	5951.66	29883.42	4367.91
immunoglobulin lambda variable 4-60	IGLV4-60	A0A075B6I1	68.75	363.08	53.17	390.79	85.53	336.76	67.01	577.87	121.75
immunoglobulin lambda variable 2-18	IGLV2-18	A0A075B6J9	13.75	29647.12	4706.91	25600.95	4145.54	33490.98	8174.03	51382.80	11187.08
immunoglobulin lambda variable 3-10	IGLV3-10	A0A075B6K4	1.25	29715.49	3458.05	35168.11	5999.88	24535.50	3524.66	28166.72	2210.28
Immunoglobulin kappa variable 2D-30	IGKV2D-30	A0A075B6S6	1.25	28532.04	2519.58	33138.77	4152.30	24155.65	2758.26	30875.49	2740.28
Immunoglobulin kappa variable 3D-15	IGKV3D-15	A0A087WSY6	13.75	56153.91	8524.83	61848.24	11501.29	50744.30	12629.39	67586.59	13484.43
immunoglobulin kappa variable 3D-11; Immunoglobulin kappa variable 3-11	IGKV3D-11; IGKV3-11	A0A0A0MRZ8; P04433	0	1011988.98	142388.26	1149607.99	257430.74	881250.92	140472.93	821854.20	59055.61
immunoglobulin heavy variable 3-49	IGHV3-49	A0A0A0MS15	1.25	43432.83	5372.70	42717.10	6088.41	44112.76	8778.83	42388.39	3852.56
Immunoglobulin kappa variable 6D-21	IGKV6D-21	A0A0A0MT36	3.75	22030.65	3046.56	25488.14	5066.09	18746.02	3538.81	21843.79	3063.08
immunoglobulin heavy variable 6-1	IGHV6-1	A0A0B4J1U7	0	101407.25	12511.90	114292.53	24019.79	89166.22	9876.46	117869.41	16768.57
Immunoglobulin heavy variable 3-15	IGHV3-15	A0A0B4J1V0	26.25	17026.80	3703.73	21353.97	5545.07	12915.98	4923.86	29626.49	6061.94
Immunoglobulin heavy variable 2-26	IGHV2-26	A0A0B4J1V2	5	6464.07	805.60	5829.54	678.35	7066.88	1414.02	5923.69	633.36
immunoglobulin heavy variable 3-74	IGHV3-74	A0A0B4J1X5	93.75	705.94	194.07	633.62	214.00	774.65	319.73	557.79	95.90
immunoglobulin heavy variable 3-72	IGHV3-72	A0A0B4J1Y9	0	125389.28	7817.81	131998.38	14050.88	119110.64	7914.37	124992.06	7297.61
immunoglobulin kappa variable 1D-13	IGHV1D-13	A0A0B4J2D9	67.5	19975.76	7648.60	5210.62	3055.60	34002.64	13764.60	23664.40	7084.88
immunoglobulin heavy variable 1-69D; Immunoglobulin heavy variable 1-69	IGHV1-69D; IGHV1-69	A0A0B4J2H0; P01742	3.75	31615.72	3388.14	31275.47	5870.70	31938.96	3826.32	33086.67	3485.88
Immunoglobulin kappa variable 6-21	IGKV6-21	A0A0C4DH24	1.25	35802.10	5443.02	31110.35	4775.40	40259.25	9459.35	36600.89	6699.97
immunoglobulin kappa variable 3D-20	IGKV3D-20	A0A0C4DH25	0	308290.33	41820.57	311344.15	58729.41	305389.19	60782.09	269811.86	27515.44
Immunoglobulin heavy variable 1-18	IGHV1-18	A0A0C4DH31	0	134331.57	14654.17	116590.67	22351.79	151185.42	19044.81	176346.25	19984.35
immunoglobulin heavy variable 5-51	IGHV5-51	A0A0C4DH38	15	114403.09	16310.46	83423.01	16353.55	143834.17	26111.81	99259.33	12646.36
immunoglobulin heavy variable 4-61; 4-39; 4-59; 4-34; 4-30-4; 4-38-2	IGHV4-61; IGHV4-39; IGHV 4-59; IGHV 4-34; IGHV 4-30-4; IGHV 4-38-2	A0A0C4DH41; P01824; P01825; P06331; P0DP06; P0DP08	0	52375.74	5279.34	52223.45	7570.40	52520.42	7548.65	42604.40	2410.64
immunoglobulin kappa variable 1-8	IGKV1-8	A0A0C4DH67	0	79653.12	7375.96	84729.71	11879.58	74830.36	9224.14	89789.46	9942.09
immunoglobulin kappa variable 2-24	IGKV2-24	A0A0C4DH68	12.5	14689.64	2134.59	17522.55	3795.43	11998.38	2116.92	16309.16	2273.11
Immunoglobulin kappa variable 1-12; immunoglobulin kappa variable 1D-12; Immunoglobulin kappa variable 1D-39	IGKV1-12; IGKV1D-12; IGKV1D-39	A0A0C4DH73; P01611; P04432	0	152674.76	11871.00	152076.30	14404.23	153243.30	18838.92	151253.39	15727.54
immunoglobulin heavy variable 5-10-1	IGHV50-10-1	A0A0J9YXX1	56.25	3421.84	810.06	4353.48	1427.94	2536.79	837.25	4020.90	1068.57
immunoglobulin lambda constant 7	IGLC7	A0M8Q6	18.75	7406.35	1472.87	10066.97	2764.83	4878.75	1092.19	8402.26	1324.11
Mannan-binding lectin serine protease 2; Isoform 2 of Mannan-binding lectin serine protease 2	MASP2	O00187-1; O00187-2	2.5	15337.07	1353.04	16305.93	1758.48	14416.66	2049.78	16417.14	1332.54
Sulfhydryl oxidase 1; Isoform 2 of Sulfhydryl oxidase 1	QSOX1	O00391; O00391-2	27.5	4115.97	464.04	4854.53	812.91	3414.34	461.05	4289.03	550.72
Neuropilin-1; Isoform 2 of Neuropilin-1; Isoform 3 of Neuropilin-1	NRP1	O14786-1; O14786-2; O14786-3	61.25	1669.31	362.78	1089.74	347.61	2219.90	599.61	2024.41	335.29
Apolipoprotein L1; Isoform 2 of Apolipoprotein L1; Isoform 3 of Apolipoprotein L1	APOL1	O14791; O14791-2; O14791-3	0	18008.47	3262.08	16271.45	4198.35	19658.64	4993.13	13105.54	1230.85
Centrosomal protein of 290 kDa; Isoform 2 of Centrosomal protein of 290 kDa	CEP290	O15078-1; O15078-2	2.5	690076.52	67464.80	652587.07	104068.88	725691.49	89333.68	781959.68	79094.19
CD5 antigen-like	CD5L	O43866	0	79767.10	7427.03	85553.76	12280.23	74269.78	8905.97	74081.47	8771.38
Ficolin-3	FCN3	O75636-1	15	24899.22	7841.44	21849.69	6771.71	27796.28	13794.38	12055.68	2757.62

Protein Name	Gene name	UniProt ID		All ME/CFS		ME/CFS with sr-IBS		ME/CFS without sr-IBS		Control	
			Undetectable values (%)	Mean	SEM	Mean	SEM	Mean	SEM	Mean	SEM
Attractin; Isoform 2 of Attractin; Isoform 3 of Attractin	ATRN	O75882-1; O75882-2; O75882-3	0	19801.09	781.55	19280.23	1172.06	20295.91	1062.10	19881.19	892.41
apolipoprotein M; Isoform 2 of Apolipoprotein M	APOM	O95445-1; O95445-2	0	35530.47	4369.90	33715.33	5433.13	37254.84	6830.23	34421.22	2831.08
Ceruloplasmin	CP	P00450	0	876306.83	45255.65	898398.83	62095.37	855319.43	66563.94	933116.55	40082.77
Coagulation factor XIII A chain	F13A1	P00488	0	13865.92	1170.90	15801.19	1962.08	12027.41	1272.17	14980.87	1416.35
Prothrombin	F2	P00734	0	1262981.74	165627.53	1314867.32	252210.51	1213690.45	223581.59	1105789.38	104690.50
Complement C1r subcomponent	C1R	P00736	0	47220.90	2673.16	48666.96	3993.12	45847.13	3660.54	45290.64	2346.85
Haptoglobin	HP	P00738	0	5340241.10	565834.07	6580744.76	1016937.67	4161762.62	454458.10	5401213.68	405173.39
Haptoglobin-related protein ;Isoform 2 of Haptoglobin-related protein	HPR	P00739-1; P00739-2	0	99488.36	19397.03	121562.35	38461.70	78518.06	12288.11	76147.27	7115.42
Coagulation factor IX	F9	P00740	0	53050.61	8257.43	67681.60	15784.96	39151.17	5572.42	47039.46	6458.26
Coagulation factor X	F10	P00742	0	76038.73	10011.21	78821.65	9049.60	73394.95	17465.43	71628.67	5581.72
Complement factor D	CFD	P00746	1.25	17588.14	1634.69	19284.41	2481.17	15976.68	2158.50	17402.54	1824.37
Plasminogen	PLG	P00747	0	466125.30	31680.58	503227.30	51683.47	430878.40	37828.22	473438.26	26120.62
Coagulation factor XII	F12	P00748	0	73202.98	4447.54	83138.36	6946.82	63764.37	5011.74	71822.97	4587.12
Complement factor B	CFB	P00751-1	0	350665.57	33211.85	397903.82	61184.06	305789.23	29153.50	346975.49	25902.54
Antithrombin-III	SERPINC1	P01008	0	279368.41	16193.41	277829.33	21739.15	280830.55	24290.04	247630.27	13390.90
alpha-1-antitrypsin	SERPINA1	P01009-1	0	298251.09	24074.38	277005.85	31295.16	318434.06	36269.90	357596.89	25599.69
Alpha-1-antichymotrypsin	SERPINA3	P01011-1	0	37193.12	4482.17	29899.39	5869.69	44122.17	6457.75	32458.85	2428.46
Angiotensinogen	AGT	P01019	0	71615.92	5939.36	77702.04	9990.96	65834.10	6821.25	69430.79	5876.69
alpha-2-macroglobulin	A2M	P01023	0	3542954.82	276170.53	3729223.78	410922.80	3365999.30	378087.26	3455762.48	250476.53
Complement C3	C3	P01024	0	859799.15	49944.43	942942.85	82618.15	780812.65	55501.83	810986.56	30257.51
Complement C5	C5	P01031	0	121860.76	6538.42	119877.28	8653.92	123745.06	9879.80	115572.16	4704.08
Cystatin-C	CST3	P01034	22.5	8674.89	1938.95	10476.26	3437.15	6963.59	2017.27	8110.40	1981.07
kininogen-1	KNG1	P01042	1.25	23562.30	3462.71	23032.86	4880.72	24065.26	5016.57	18843.42	4499.31
Isoform LMW of Kininogen-1	Isoform LMW KNG1	P01042-2	0	741701.52	46110.66	846693.84	72090.48	641958.83	51512.50	764789.46	40267.27
Insulin-like growth factor II; Isoform 2 of Insulin-like growth factor II; Isoform 3 of Insulin-like growth factor II	IGF2	P01344; P01344-2; P01344-3	0	60076.05	8133.28	62313.40	11857.90	57950.58	11448.01	65009.06	8920.76
Immunoglobulin J chain	JCHAIN	P01591	0	413358.16	36751.94	432571.19	48865.35	395105.78	55169.22	410095.64	39208.18
Immunoglobulin kappa variable 1-33	IGKV1-33	P01594	0	323526.01	32771.72	334129.19	43470.92	313452.99	49438.25	313234.42	31599.99
Immunoglobulin kappa variable 1-17	IGKV1-17	P01599	1.25	21645.67	2990.02	24007.64	3667.26	19401.80	4665.59	16128.74	1188.19
Immunoglobulin kappa variable 1D-16	IGKV1D-16	P01601	16.25	53988.86	6765.99	52205.89	7313.81	55682.67	11238.44	45819.29	6808.66
Immunoglobulin kappa variable 1-5	IGKV1-5	P01602	6.25	16031.19	1573.51	16646.59	2249.26	15446.57	2247.88	16749.70	1289.95
Immunoglobulin kappa variable 2D-40	IGKV2D-40	P01614	1.25	85402.53	10556.85	98024.10	17516.49	73412.04	12247.47	85819.31	7658.41
Immunoglobulin kappa variable 3-20	IGKV3-20	P01619	0	423363.06	56603.14	427293.79	66453.02	419628.87	91175.08	367673.60	34360.45
Immunoglobulin kappa variable 3-15	IGKV3-15	P01624	1.25	878700.86	121387.61	1031751.70	168346.94	733302.56	171875.49	1024607.03	139840.91
Immunoglobulin lambda variable 1-44	IGLV1-44	P01699	6.25	2479761.79	510506.68	2702731.59	740607.55	2267940.48	719716.39	1816608.11	470063.87
Immunoglobulin lambda variable 1-47	IGLV1-47	P01700	1.25	92804.34	7944.74	103665.26	13458.23	82486.46	8741.28	115326.20	8213.04
immunoglobulin lambda variable 1-51	IGLV1-51	P01701	7.5	33440.96	4351.41	34620.88	5410.37	32320.04	6813.96	36716.73	5270.51
Immunoglobulin lambda variable 2-23	IGLV2-23	P01705	35	4486.18	1019.08	4744.24	2019.98	4241.01	733.38	7947.84	2955.13
Immunoglobulin lambda variable 2-11	IGLV2-11	P01706	21.25	12601.02	1954.80	12189.81	2594.03	12991.67	2951.82	18757.69	2830.94
Immunoglobulin lambda variable 2-8	IGLV2-8	P01709	10	29947.67	5254.26	36192.55	7671.67	24015.03	7150.40	23552.34	2935.55
Immunoglobulin lambda variable 3-19	IGLV3-19	P01714	0	302840.18	50158.21	346006.28	85097.88	261832.39	57368.23	274119.20	27654.62
immunoglobulin lambda variable 3-27	IGLV3-27	P01718	10	10900.74	1355.15	9351.47	1724.97	12372.55	2044.97	10862.10	1304.16
Immunoglobulin heavy variable 3-23; Immunoglobulin heavy variable 3-30	IGHV3-23; IGHV3-30	P01764; P01768	1.25	82435.67	8007.91	77520.62	6420.51	87104.98	14229.09	74742.43	5166.69
Immunoglobulin heavy variable 3-13	IGHV3-13	P01766	7.5	22552.46	2704.67	23400.77	3653.43	21746.58	4033.05	22079.44	2328.77
Immunoglobulin heavy variable 3-7	IGHV3-7	P01780	8.75	6462.21	597.04	5385.25	710.19	7485.31	898.16	6886.52	581.63

Protein Name	Gene name	UniProt ID		All ME/CFS		ME/CFS with sr-IBS		ME/CFS without sr-IBS		Control	
			Undetectable values (%)	Mean	SEM	Mean	SEM	Mean	SEM	Mean	SEM
Immunoglobulin heavy variable 3-9	IGHV3-9	P01782	0	101637.70	7573.97	93473.35	7377.26	109393.84	12754.86	98489.40	7950.39
Polymeric immunoglobulin receptor	PIGR	P01833	60	3212.71	636.74	4305.08	943.78	2174.95	819.64	1298.53	437.70
immunoglobulin kappa constant	IGKC	P01834	1.25	765213.02	61187.68	829845.23	71398.96	703812.42	96842.98	895005.99	70488.61
Immunoglobulin heavy constant gamma 2	IGHG2	P01859	0	5597162.89	533194.72	5947192.05	782587.73	5264635.19	739769.80	5194346.01	433157.19
Immunoglobulin heavy constant gamma 3	IGHG3	P01860	0	2131681.37	276203.69	2492758.74	466977.17	1788657.87	306876.63	1919148.06	191032.10
Immunoglobulin heavy constant gamma 4	IGHG4	P01861	0	282319.11	39438.81	301619.20	57100.75	263984.01	55633.28	361566.36	49811.48
immunoglobulin heavy constant mu; Isoform 2 of Immunoglobulin heavy constant mu	IGHM	P01871; P01871-2	0	2652640.00	317819.82	2720145.13	431878.13	2588510.12	472542.03	2959631.20	410127.84
immunoglobulin heavy constant alpha 1	IGHA1	P01876	0	6032128.86	658454.39	6828149.87	983078.52	5275908.90	875644.83	6509349.49	585008.42
Immunoglobulin heavy constant alpha 2	IGHA2	P01877	0	280775.10	34623.20	332288.41	55926.92	231837.46	40819.94	241664.24	23891.07
Keratin, type I cytoskeletal 14; Keratin, type I cytoskeletal 16	KRT14; KRT16	P02533; P08779	92.5	1721.63	209.80	1511.83	0.00	1920.94	399.25	4945.22	2968.46
Apolipoprotein A-I	APOA1	P02647	0	4315491.49	287110.96	5055802.04	419401.59	3612196.48	335340.34	4106073.93	206767.79
Apolipoprotein E	APOE	P02649	0	172355.51	13809.08	187147.58	18718.40	158303.05	20085.91	165274.90	13902.63
Apolipoprotein A-II	APOA2	P02652	0	563317.54	45177.18	617104.79	66269.74	512219.65	61204.22	566398.30	37587.99
Apolipoprotein C-I	APOC1	P02654	0	99472.57	9145.69	114563.76	16296.55	85135.94	8594.87	100369.85	10954.22
Apolipoprotein C-II	APOC2	P02655	1.25	150339.47	13218.74	155509.74	21976.22	145427.72	15948.68	141494.13	7758.64
Apolipoprotein C-III	APOC3	P02656	0	1542488.19	109352.75	1717165.46	131213.95	1376544.78	166136.82	1400815.11	117227.91
Fibrinogen alpha chain	FGA	P02671-1	0	2370805.49	234426.13	2382481.85	290541.32	2359712.95	368186.45	1958743.02	214592.53
Fibrinogen beta chain	FGB	P02675	0	2066551.80	84165.65	2101250.61	101818.54	2033587.93	133346.70	2174903.87	87877.75
Fibrinogen gamma chain; Isoform Gamma-A of Fibrinogen gamma chain	FGG	P02679; P02679-2	0	946241.86	53672.36	981770.75	98062.34	912489.42	52668.29	948036.67	39536.07
Serum amyloid P-component	APCS	P02743	2.5	10193.03	645.67	10635.50	938.99	9772.67	902.39	9495.30	627.62
Complement C1q subcomponent subunit A	C1QA	P02745	17.5	16308.15	2578.40	14923.79	3922.03	17623.29	3467.60	16287.37	1728.77
Complement C1q subcomponent subunit B	C1QB	P02746	0	85901.02	4597.81	84100.83	6323.52	87611.21	6763.73	92755.32	4089.47
Complement C1q subcomponent subunit C	C1QC	P02747	0	154715.24	6646.57	160964.34	9574.61	148778.60	9274.60	175908.28	9587.81
complement component C9	C9	P02748	0	57271.59	4699.61	60054.65	7728.52	54627.69	5728.27	54778.46	3438.43
Beta-2-glycoprotein 1	APOH	P02749	0	521565.85	66402.20	578172.80	98030.52	467789.24	90972.97	430563.53	38979.08
Leucine-rich alpha-2-glycoprotein	LRG1	P02750	0	168498.10	14936.65	179159.31	20345.61	158369.95	21954.06	209092.88	14012.27
fibronectin; Isoform 11 of Fibronectin; Isoform 14 of Fibronectin; Isoform 15 of Fibronectin; Isoform 3 of Fibronectin; Isoform 7 of Fibronectin; Isoform 8 of Fibronectin	FN1	P02751; P02751-11; P02751-14; P02751-15; P02751-3; P02751-7; P02751-8	0	519915.48	51582.63	563175.16	86271.90	478818.79	60541.46	565253.08	57738.82
Retinol-binding protein 4	RBP4	P02753	0	216747.62	18730.38	244011.42	29650.92	190847.01	22806.40	225034.53	18771.69
Protein AMBP	AMBP	P02760	0	1717661.15	144722.51	1885308.72	245102.10	1558395.95	162485.96	1754894.78	136838.95
Alpha-1-acid glycoprotein 1	ORM1	P02763	0	129238.72	13289.78	137068.27	19420.89	121800.65	18548.43	142580.62	16683.29
Alpha-2-HS-glycoprotein	AHSG	P02765	0	1777913.03	218998.18	1823431.01	295251.38	1734670.96	327350.11	1695715.85	147709.28
Transthyretin	TTR	P02766	0	366940.63	26590.96	382594.05	31736.66	352069.88	42256.97	403933.63	25122.31
Serum albumin	ALB	P02768-1	0	80587844.00	5078241.41	75932053.47	6129170.15	85010845.00	7954110.31	79326887.51	4175792.27
vitamin D-binding protein; Isoform 3 of Vitamin D-binding protein	GC	P02774; P02774-3	0	956663.82	76833.75	1020969.85	125946.30	895573.10	93071.04	1057892.42	66615.35
Platelet basic protein	PPBP	P02775	0	248428.76	25844.77	288007.08	42978.99	210829.35	28905.57	260225.64	22778.86

Protein Name	Gene name	UniProt ID		All ME/CFS		ME/CFS with sr-IBS		ME/CFS without sr-IBS		Control	
			Undetectable values (%)	Mean	SEM	Mean	SEM	Mean	SEM	Mean	SEM
Platelet factor 4	PF4	P02776	2.5	18556.33	1731.46	23068.05	2473.21	14270.19	2068.34	20425.90	1609.20
Serotransferrin	TF	P02787	0	8170880.51	802790.97	9478314.51	1297443.01	6928818.21	931714.57	7782539.29	601370.59
Hemopexin	HPX	P02790	0	4085539.32	223907.93	4457633.95	313361.19	3732049.43	305975.27	4419428.68	232785.29
angiogenin	ANG	P03950	27.5	11071.16	3445.66	8389.04	5198.21	13619.18	4635.52	6387.79	1411.28
Coagulation factor XI; Isoform 2 of Coagulation factor XI	F11	P03951; P03951-2	0	12819.47	1902.07	14495.39	3340.84	11227.36	2028.23	9946.00	673.81
Plasma kallikrein	KLKB1	P03952	0	125948.13	9791.25	122912.95	12124.67	128831.55	15357.52	117536.39	6648.87
C4b-binding protein alpha chain	C4BPA	P04003	0	223380.35	19963.92	212045.38	32819.07	234148.57	24367.97	198775.95	11153.72
Vitronectin	VTN	P04004	0	1125958.98	102857.71	1204586.70	153077.27	1051262.65	140335.22	951617.75	68798.23
Vitamin K-dependent protein C; Isoform 2 of Vitamin K-dependent protein C	PROC	P04070; P04070-2	3.75	5479.66	612.31	5383.91	758.11	5570.62	961.79	5037.10	382.40
fructose-bisphosphate aldolase A; Isoform 2 of Fructose-bisphosphate aldolase A	ALDOA	P04075; P04075-2	5	11326.50	1174.01	13647.76	1839.72	9121.30	1367.72	11240.24	872.91
apolipoprotein B-100	APOB	P04114	0	189431.44	11604.62	184130.54	20353.56	194467.30	12695.89	172557.52	10032.00
phosphatidylcholine-sterol acyltransferase	LCAT	P04180	12.5	9238.58	860.81	7717.09	1216.54	10684.00	1155.64	8217.71	690.40
Histidine-rich glycoprotein	HRG	P04196	0	397342.79	85791.19	495411.33	166190.62	304177.68	63378.63	309742.34	30810.15
Immunoglobulin lambda variable 7-43	IGLV7-43	P04211	10	7409.61	3629.65	11279.45	7608.15	3733.26	734.38	3648.29	499.64
Alpha-1B-glycoprotein	A1BG	P04217	0	1506481.69	153375.47	1683578.57	257455.93	1338239.66	175038.39	1607917.01	137216.44
Keratin, type II cytoskeletal 1	KRT1	P04264	40	5220.25	928.87	5529.77	1554.09	4926.20	1111.10	7532.57	2152.78
Von Willebrand factor	VWF	P04275	0	11851.15	1209.20	10743.52	1089.07	12903.40	2086.58	14803.30	1435.25
Sex hormone-binding globulin	SHBG	P04278-1	3.75	19728.65	2366.06	20097.49	3561.44	19378.25	3237.46	24514.38	2629.88
insulin-like growth factor I; Isoform 2 of Insulin-like growth factor I; Isoform 3 of Insulin-like growth factor I; Isoform 4 of Insulin-like growth factor I	IGF1	P05019; P05019-2; P05019-3; P05019-4	16.25	10229.09	1653.22	10024.83	2420.70	10423.14	2321.51	11186.49	1298.34
fructose-bisphosphate aldolase B	ALDOB	P05062	65	2746.31	637.98	3355.30	1205.42	2167.77	545.36	3175.25	720.90
apolipoprotein D	APOD	P05090	0	308188.78	32667.51	326316.87	50196.93	290967.09	43441.31	360259.75	32927.88
Plasma serine protease inhibitor	SERPINA5	P05154	2.5	6566.14	910.80	5252.73	1069.53	7813.88	1411.45	10578.00	1750.05
Plasma protease C1 inhibitor; Isoform 2 of Plasma protease C1 inhibitor; Isoform 3 of Plasma protease C1 inhibitor	SERPING1	P05155; P05155-2; P05155-3	0	508551.13	15128.64	483284.65	22978.59	532554.28	19028.02	521204.93	17139.35
Complement factor I	CFI	P05156	0	80597.36	6104.37	88909.54	10221.55	72700.79	6866.60	76755.20	4361.16
Coagulation factor XIII B chain	F13B	P05160	0	33601.44	2819.00	32812.57	3863.51	34350.86	4163.87	35455.11	3177.40
Tetranectin	CLEC3B	P05452	0	47485.06	5296.03	44831.93	6360.09	50005.53	8406.97	49338.44	4607.73
thyroxine-binding globulin	SERPINA7	P05543	26.25	3814.45	528.64	3421.83	698.02	4187.45	792.50	3999.31	575.95
Heparin cofactor 2	SERPIND1	P05546	0	63244.48	5818.91	57641.07	6959.67	68567.73	9138.75	63006.39	3809.24
immunoglobulin kappa variable 4-1	IGKV4-1	P06312	0	177064.58	16487.85	185845.80	21974.96	168722.43	24707.03	158449.29	14793.52
Gelsolin	GSN	P06396	0	75601.46	3410.88	72786.94	4987.99	78275.26	4719.48	88248.34	5357.07
ATP synthase subunit beta, mitochondrial	ATP5F1B	P06576	57.5	2663.87	471.63	2570.49	690.32	2752.58	662.13	3318.53	600.49
complement C2	C2	P06681-1	0	47401.08	6798.83	54057.86	11686.17	41077.14	7528.51	40849.38	3712.11
Apolipoprotein A-IV	APOA4	P06727	0	424152.66	23466.22	425599.09	33383.69	422778.55	33766.01	475572.25	20544.97
Vitamin K-dependent protein S	PROS1	P07225	0	42366.36	2081.09	42645.92	3338.25	42100.77	2651.39	43607.60	2103.09
Complement component C8 alpha chain	C8A	P07357	0	41289.91	2845.05	39681.54	3681.79	42817.86	4337.85	39413.33	2249.77
Complement component C8 beta chain	C8B	P07358	0	117664.16	17599.50	120551.21	32169.36	114921.47	17588.45	104508.65	7637.92
Complement component C8 gamma chain	C8G	P07360	0	61322.38	6632.96	53600.11	6389.75	68658.54	11167.93	52349.30	3004.35

Protein Name	Gene name	UniProt ID		All ME/CFS		ME/CFS with sr-IBS		ME/CFS without sr-IBS		Control	
			Undetectable values (%)	Mean	SEM	Mean	SEM	Mean	SEM	Mean	SEM
profilin-1	PFN1	P07737	7.5	32833.58	3670.21	36035.19	4944.86	29792.06	5406.09	34839.06	2715.06
thrombospondin-1 ;Isoform 2 of Thrombospondin-1	THBS1	P07996; P07996-2	2.5	50477.48	5342.89	59348.28	8842.46	42050.23	5935.55	53394.92	4581.64
corticosteroid-binding globulin	SERPINA6	P08185	16.25	9285.62	1317.92	8213.00	1496.26	10304.60	2127.58	10101.21	991.92
apolipoprotein(a)	LPA	P08519	0	86745.86	17538.55	64787.09	8702.53	107606.69	32190.75	84705.24	14409.07
pleckstrin	PLEK	P08567	15	17176.92	3261.63	20246.07	5455.54	14261.22	3804.00	21466.86	3215.80
Monocyte differentiation antigen CD14	CD14	P08571	1.25	18609.87	2059.93	15995.88	2062.75	21093.15	3416.31	22830.57	2181.04
complement factor H	CFH	P08603-1	0	701951.18	72056.72	821472.14	127210.59	588406.26	69411.91	636086.48	44239.18
Low affinity immunoglobulin gamma Fc region receptor III-A	FCGR3A	P08637	15	15779.69	2673.19	17066.15	3979.76	14557.55	3677.92	14176.19	2153.25
Alpha-2-antiplasmin	SERPINF2	P08697-1	0	58231.92	2381.87	55939.21	3936.86	60410.00	2828.18	56736.03	1600.93
Complement C1s subcomponent	C1S	P09871	0	178607.62	18762.16	185318.57	29167.12	172232.22	24748.85	155357.60	10628.03
Complement C4-A	C4A	P0C0L4-1	0	1059020.06	154220.55	1090805.49	201324.24	1028823.90	235231.63	816924.94	73105.43
Complement C4-B	C4B	P0C0L5	0	66982.90	6483.46	73436.21	7363.58	60852.26	10403.53	64440.99	5317.47
Serum amyloid A-1 protein	SAA1	P0DJI8	2.5	49120.45	21315.31	39058.61	7312.54	58679.19	40431.85	29584.46	5105.66
Immunoglobulin alpha-2 heavy chain	IGHA2	P0DOX2	0	2615292.15	257368.21	2487839.66	381536.73	2736372.01	355404.70	3427502.72	304298.03
immunoglobulin delta heavy chain	IGD	P0DOX3	12.5	26100.28	8402.35	29384.11	15786.54	22980.63	7671.74	14555.29	3154.61
immunoglobulin gamma-1 heavy chain	IGG1	P0DOX5	0	4560582.70	295622.24	4660833.38	369606.97	4465344.56	461233.84	5207112.68	299926.68
immunoglobulin mu heavy chain	IGM	P0DOX6	7.5	35377.55	4921.56	34972.91	7533.26	35761.96	6623.33	37820.79	5500.86
immunoglobulin kappa light chain	IGL	P0DOX7	0	3138728.87	314150.59	3480832.60	412633.39	2813730.33	466300.50	3093505.19	327466.54
Immunoglobulin lambda-1 light chain	IGK	P0DOX8	0	3374446.92	331117.74	3637989.12	400185.75	3124081.84	520322.55	3483071.13	304539.55
immunoglobulin lambda constant 3	IGLC3	P0DOY3	0	1050950.09	95074.81	1058760.82	112984.59	1043529.89	152323.98	1072381.99	87086.04
Complement component C7	C7	P10643	0	48281.16	3883.93	52710.59	5258.16	44073.21	5635.31	45576.72	3916.79
Clusterin; Isoform 2 of Clusterin; Isoform 4 of Clusterin; Isoform 5 of Clusterin	CLU	P10909-1; P10909-2; P10909-4; P10909-5	0	250123.62	11856.12	243170.93	17879.08	256728.67	16063.76	260727.13	10362.43
Heat shock cognate 71 kDa protein; Isoform 2 of Heat shock cognate 71 kDa protein; Heat shock-related 70 kDa protein 2	HSPA8; HSPA2	P11142-1; P11142-2; P54652	15	5404.32	479.36	5614.84	729.12	5204.33	646.30	6175.40	526.12
Mannose-binding protein C	MBL2	P11226	23.75	6664.67	785.69	6322.08	975.33	6990.13	1228.63	5549.18	669.31
cholesteryl ester transfer protein; Isoform 2 of Cholesteryl ester transfer protein	CETP	P11597-1; P11597-2	28.75	4312.85	2136.30	6669.94	4461.26	2073.62	523.08	3443.02	484.01
Coagulation factor V	F5	P12259	0	9153.19	396.12	8870.69	607.53	9421.57	525.07	8496.39	381.56
Keratin, type I cytoskeletal 10	KRT10	P13645	65	4817.48	2084.51	5559.20	2997.42	4112.85	2965.20	6256.39	2082.01
Complement component c6	C6	P13671	0	145616.99	9692.62	163761.08	15628.98	128380.12	10955.63	163240.59	9712.41
L-selectin ;Isoform 2 of L-selectin	SELL	P14151; P14151-2	0	49374.96	8754.73	55594.46	15021.33	43466.44	9892.62	41556.27	5786.89
Pyruvate kinase PKM	PKM	P14618	2.5	29497.63	12591.08	13033.10	2752.73	45138.94	23611.87	83489.15	44899.34
Poliovirus receptor; Isoform Beta of Poliovirus receptor; Isoform Gamma of Poliovirus receptor; Isoform Delta of Poliovirus receptor	PVR	P15151-1; P15151-2; P15151-3; P15151-4	27.5	1730.92	259.40	1868.15	343.49	1600.54	390.36	2179.05	276.76
Ras-related C3 botulinum toxin substrate 2 ;Ras-related C3 botulinum toxin substrate 3 ;Ras-related C3 botulinum toxin substrate 1 ;Isoform B of Ras-related C3 botulinum toxin substrate 1	RAC2; RAC3; RAC1	P15153; P60763; P63000-1; P63000-2	90	629.46	47.32	602.48	66.98	655.08	67.88	769.14	140.72
Carboxypeptidase N catalytic chain	CPN1	P15169	2.5	15720.87	1288.24	15385.36	1730.48	16039.61	1929.24	16366.36	2133.86

Protein Name	Gene name	UniProt ID		All ME/CFS		ME/CFS with sr-IBS		ME/CFS without sr-IBS		Control	
			Undetectable values (%)	Mean	SEM	Mean	SEM	Mean	SEM	Mean	SEM
Immunoglobulin lambda-like polypeptide 1	IGLL1	P15814	32.5	15675.30	2856.42	16332.95	4154.01	15050.52	4031.49	14996.26	2269.73
CD44 antigen; Isoform 10 of CD44 antigen; Isoform 11 of CD44 antigen; Isoform 12 of CD44 antigen; Isoform 13 of CD44 antigen; Isoform 14 of CD44 antigen; Isoform 15 of CD44 antigen; Isoform 16 of CD44 antigen; Isoform 17 of CD44 antigen; Isoform 18 of CD44 antigen; Isoform 3 of CD44 antigen; Isoform 4 of CD44 antigen; Isoform 5 of CD44 antigen; Isoform 6 of CD44 antigen; Isoform 7 of CD44 antigen; Isoform 8 of CD44 antigen; Isoform 9 of CD44 antigen	CD44	P16070; P16070-10; P16070-11; P16070-12; P16070-13; P16070-14; P16070-15; P16070-16; P16070-17; P16070-18; P16070-3; P16070-4; P16070-5; P16070-6; P16070-7; P16070-8; P16070-9	0	21119.54	1667.75	22548.31	2583.95	19762.21	2172.46	22574.00	1982.21
Heat shock 70 kDa protein 6; Putative heat shock 70 kDa protein 7	HSPA6; HSPA7	P17066; P48741	50	2876.96	475.34	2516.05	649.52	3219.84	696.12	3475.22	819.21
Insulin-like growth factor-binding protein 3; Isoform 2 of Insulin-like growth factor-binding protein 3	IGFBP3	P17936; P17936-2	0	38280.67	4040.65	40827.36	6314.13	35861.30	5259.86	40066.61	3068.30
lipopolysaccharide-binding protein	LBP	P18428	52.5	4381.83	774.82	4262.03	1152.96	4495.63	1071.94	2241.64	459.80
Alpha-1-acid glycoprotein 2	ORM2	P19652	0	35309.48	4636.91	43845.01	8387.25	27200.72	3996.57	41936.30	6391.85
Inter-alpha-trypsin inhibitor heavy chain H2	ITIH2	P19823	0	696961.85	33118.27	690085.52	46515.35	703494.37	48088.88	810918.24	36544.51
Inter-alpha-trypsin inhibitor heavy chain H1	ITIH1	P19827-1	0	408975.65	26899.76	390062.30	31104.36	426943.34	43229.69	399317.80	23834.27
Pregnancy zone protein	PZP	P20742	0	198715.97	17261.75	175812.66	18807.94	220474.12	27762.97	175387.15	14697.46
C4b-binding protein beta chain; Isoform 2 of C4b-binding protein beta chain	C4BPB	P20851; P20851-2	0	35343.52	2724.86	37499.69	4601.38	33295.16	3154.90	27425.76	1848.94
Filamin-A; Isoform 2 of Filamin-A	FLNA	P21333; P21333-2	11.25	5650.13	708.76	6897.15	1268.94	4465.45	643.34	5369.13	474.25
Glutathione peroxidase 3	GPX3	P22352	2.5	35166.26	4305.04	39507.54	7353.24	31042.06	4814.73	38589.44	5067.41
Carboxypeptidase n subunit 2	CPN2	P22792	0	44441.47	1806.87	43949.72	2423.40	44908.63	2708.31	43062.86	1951.11
Vitamin K-dependent protein Z; Isoform 2 of Vitamin K-dependent protein Z	PROZ	P22891-1; P22891-2	3.75	9782.24	1059.49	10789.07	1707.88	8825.76	1306.38	10983.98	1041.50
Immunoglobulin heavy variable 1-2	IGHV1-2	P23083	62.5	4350.94	1047.30	5922.18	1945.90	2858.26	877.48	3828.04	814.65
Fibulin-1	FBLN1	P23142	0	38859.18	4502.03	44364.38	8217.88	33629.24	4198.66	44110.35	4828.21
Isoform C of Fibulin-1	FBLN1	P23142-4	5	56901.24	7336.13	65649.51	11140.87	48590.38	9591.83	58980.45	7140.06
Cofilin-1	CFL1	P23528	63.75	3218.34	926.16	3787.94	1589.04	2677.23	1051.42	4416.22	992.04
Insulin-like growth factor-binding protein 5	IGFBP5	P24593	66.25	2549.50	494.56	2659.49	727.94	2445.02	690.70	2248.56	593.65
Zinc-alpha-2-glycoprotein	AZGP1	P25311	0	388309.86	60650.22	471943.59	96568.99	308857.81	73760.90	294024.75	57048.73
Hepatocyte growth factor-like protein	MST1	P26927	0	9477.45	1435.46	10508.15	2677.28	8498.29	1316.93	8227.95	541.39
Serum paraoxonase/arylesterase 1	PON1	P27169	0	143174.68	8821.33	124450.34	13585.74	160962.79	10324.56	134793.05	7567.72
Properdin	CFP	P27918	0	66630.55	15683.13	77838.04	27929.40	55983.44	16385.31	58316.63	14032.69
Kallistatin	SERPINA4	P29622	7.5	28531.33	10291.28	29425.21	17923.01	27682.14	11508.94	14592.48	4471.06
Peroxiredoxin-6	PRDX6	P30041	70	1966.59	259.11	1761.58	357.11	2161.34	376.70	1713.54	318.65
Protein disulfide-isomerase A3	PDIA3	P30101	11.25	9252.01	1524.82	12414.09	2820.57	6248.04	1116.87	10055.96	1767.39
Keratin, type I cytoskeletal 9	KRT9	P35527	41.25	4740.40	1180.90	6596.27	2253.74	2977.32	857.49	10664.02	5533.22
Serum amyloid A-4 protein	SAA4	P35542	0	70188.46	5425.38	72159.81	9419.63	68315.67	6075.96	64135.95	7464.73

Protein Name	Gene name	UniProt ID		All ME/CFS		ME/CFS with sr-IBS		ME/CFS without sr-IBS		Control	
			Undetectable values (%)	Mean	SEM	Mean	SEM	Mean	SEM	Mean	SEM
Insulin-like growth factor-binding protein complex acid labile subunit; Isoform 2 of Insulin-like growth factor-binding protein complex acid labile subunit	IGFALS	P35858; P35858-2	0	61314.56	3362.90	59840.11	5218.76	62715.29	4437.03	66936.72	2505.06
Keratin, type II cytoskeletal 2 epidermal	KRT2	P35908	57.5	2114.77	474.68	1850.46	686.18	2365.85	669.38	1573.25	310.13
Pigment epithelium-derived factor	SERPINF1	P36955	0	36922.23	1883.34	36924.95	2224.68	36919.65	3025.81	35861.68	1918.84
Complement factor H-related protein 2	CFHR2	P36980-1	0	50587.76	5336.75	59667.83	8835.57	41961.68	5893.85	46335.04	4036.84
Prostaglandin-H2 D-isomerase	PTGDS	P41222	70	4038.55	1135.31	4182.99	1711.22	3901.32	1551.65	4668.83	1584.80
Biotinidase; Isoform 2 of Biotinidase; Isoform 3 of Biotinidase; Isoform 4 of Biotinidase	BTD	P43251; P43251-2; P43251-3; P43251-4	2.5	27590.06	6127.85	31353.26	10210.20	24015.03	7314.75	27911.55	3772.84
Afamin	AFM	P43652	0	145109.53	8065.44	139196.79	10787.58	150726.64	11994.79	132551.20	5337.60
Mannan-binding lectin serine protease 1	MASP1	P48740-1	15	7009.53	981.57	8349.73	1854.98	5736.34	785.94	7408.18	770.79
Isoform 2 of Mannan-binding lectin serine protease 1; Isoform 4 of Mannan-binding lectin serine protease 1	MASP1	P48740-2; P48740-4	51.25	3557.53	1134.99	2519.13	1003.87	4544.01	1966.29	2302.66	487.47
Selenoprotein P	SELENOP	P49908	0	34312.20	3035.82	36430.77	3625.79	32299.56	4810.37	32934.65	2019.77
cathelicidin antimicrobial peptide	CAMP	P49913	17.5	9274.65	1739.30	11990.83	3347.66	6694.27	1204.55	6104.31	1288.88
Lumican	LUM	P51884	0	77327.23	6286.91	73885.58	6351.46	80596.81	10627.80	74076.13	4914.87
Cysteine-rich secretory protein 3; Isoform 2 of Cysteine-rich secretory protein 3; Isoform 3 of Cysteine-rich secretory protein 3	CRISP3	P54108-1; P54108-2; P54108-3	63.75	2651.41	511.45	2690.49	794.49	2614.28	677.41	1696.74	384.43
Apolipoprotein C-IV	APOC4	P55056	42.5	3665.65	520.08	4380.55	831.18	2986.49	629.43	3450.66	461.77
phospholipid transfer protein	PLTP	P55058	55	4210.39	1256.95	3674.52	1344.68	4719.48	2091.26	3956.67	1100.08
cadherin-13; Isoform 4 of Cadherin-13	CDH13	P55290; P55290-4	46.25	2865.19	505.05	2965.47	671.83	2769.92	761.56	3198.64	623.64
Neutrophil defensin 1; Neutrophil defensin 3	DEFA1; DEFA1; DEFA1B	P59665; P59666	0	28734.67	5911.68	27330.08	6407.56	30069.03	9812.44	24030.77	3200.10
Actin, cytoplasmic 1; Actin, cytoplasmic 2	ACTB; ACTG1	P60709; P63261	0	456456.59	40601.44	529222.40	66314.60	387329.08	45389.90	518261.61	36718.46
Ras-related protein Rap-1b; Isoform 2 of Ras-related protein Rap-1b; Isoform 3 of Ras-related protein Rap-1b; Isoform 4 of Ras-related protein Rap-1b; ras-related protein Rap-1A	RAP1A; RAP1B	P61224-1; P61224-2; P61224-3; P61224-4; P62834	23.75	5200.14	668.14	5154.74	981.32	5243.27	935.93	6858.13	739.46
Beta-2-microglobulin	B2M	P61769	31.25	9494.04	1324.28	10153.80	1606.26	8867.26	2092.44	7044.39	867.37
peptidyl-prolyl cis-trans isomerase A	PPIA	P62937	61.25	4873.95	1192.39	5128.37	1539.81	4632.25	1830.19	3418.68	818.19
14-3-3 protein zeta/delta	YWHAZ	P63104-1	12.5	4462.14	512.58	3674.01	582.41	5210.86	801.72	4432.52	337.55
Tubulin alpha-1B chain; Isoform 2 of Tubulin alpha-1B chain; tubulin alpha-1A chain; Isoform 2 of Tubulin alpha-1A chain; Tubulin alpha-1C chain	TUBA1B; TUBA1A; TUBA1C	P68363; P68363-2; Q71U36; Q71U36-2; Q9BQE3	21.25	22056.60	3373.86	25333.10	5600.54	18943.91	3976.49	35456.35	5305.46
Hemoglobin subunit beta	HBB	P68871	0	156546.21	13915.97	160527.28	22172.33	152764.20	17849.70	131095.20	10251.00
Hemoglobin subunit alpha	HBA1	P69905	0	61582.81	9511.88	66037.40	17840.27	57350.96	8704.11	52512.79	6254.46
Phosphatidylinositol-glycan-specific phospholipase D	GPLD1	P80108	0	18397.60	1727.74	19601.46	2787.61	17253.93	2154.71	14296.16	957.30
Immunoglobulin lambda variable 3-21	IGLV3-21	P80748	0	66629.09	6971.70	60928.32	8878.03	72044.82	10654.11	73277.36	6805.59

Protein Name	Gene name	UniProt ID		All ME/CFS		ME/CFS with sr-IBS		ME/CFS without sr-IBS		Control	
			Undetectable values (%)	Mean	SEM	Mean	SEM	Mean	SEM	Mean	SEM
Complement factor H-related protein 3; Isoform 2 of Complement factor H-related protein 3	CFHR3	Q02985-1; Q02985-2	76.25	1300.45	1000.98	213.26	60.69	2333.29	1899.68	9350.74	8918.97
Complement factor H-related protein 1	CFHR1	Q03591	0	43367.75	3613.07	47900.08	6063.11	39062.04	4090.05	46416.13	4645.42
Hepatocyte growtH factor activator	HGFAC	Q04756	2.5	19719.93	2391.06	20752.03	3875.17	18739.43	2990.33	20212.80	1822.33
Inter-alpha-trypsin inhibitor heavy chain H3; Isoform 2 of Inter-alpha-trypsin inhibitor heavy chain H3	ITIH3	Q06033-1; Q06033-2	1.25	6108.15	856.78	6067.85	1378.77	6146.43	1087.54	5840.60	618.64
peroxiredoxin-1	PRDX1	Q06830	60	3128.21	682.02	2237.21	921.41	3974.66	982.12	3907.81	835.95
DNA polymerase epsilon catalytic subunit A	POLE	Q07864	2.5	78569.00	11962.41	56173.59	8328.38	99844.63	20703.01	72087.78	10817.12
Galectin-3-binding protein	LGALS3BP	Q08380	0	8909.20	1116.16	10056.85	1766.99	7818.92	1403.44	6865.68	652.22
EGF-containing fibulin-like extracellular matrix protein 1; Isoform 2 of EGF-containing fibulin-like extracellular matrix protein 1; Isoform 3 of EGF-containing fibulin-like extracellular matrix protein 1; Isoform 4 of EGF-containing fibulin-like extracellular matrix protein 1; Isoform 5 of EGF-containing fibulin-like extracellular matrix protein 1	EFEMP1	Q12805; Q12805-2; Q12805-3; Q12805-4; Q12805-5	0	23540.71	1911.51	24751.96	2519.41	22390.01	2877.67	25497.41	2134.07
Src substrate cortactin; Isoform 2 of Src substrate cortactin; Isoform 3 of Src substrate cortactin	CTTN	Q14247-1; Q14247-2; Q14247-3	81.25	904.32	125.68	748.12	106.86	1052.71	217.10	885.18	156.98
Hyaluronan-binding protein 2; Isoform 2 of Hyaluronan-binding protein 2	HABP2	Q14520-1; Q14520-2	0	124676.88	14081.94	135985.16	20586.83	113934.01	19506.91	100859.23	8731.29
Inter-alpha-trypsin inhibitor heavy chain H4	ITIH4	Q14624-1	0	308034.60	38463.67	314013.28	41092.51	302354.86	64191.94	270788.88	9378.01
Isoform 2 of Inter-alpha-trypsin inhibitor heavy chain H4; Isoform 3 of Inter-alpha-trypsin inhibitor heavy chain H4	ITIH4	Q14624-2; Q14624-3	0	82115.19	17840.94	89014.31	34412.08	75561.02	14704.92	94906.66	14605.43
Procollagen C-endopeptidase enhancer 1	PCOLCE	Q15113	51.25	4446.45	1103.67	5038.21	1313.22	3884.28	1758.25	5687.16	1064.50
Serum paraoxonase/lactonase 3	PON3	Q15166	1.25	118563.14	10018.51	115184.54	17653.91	121772.80	10893.44	95418.60	7588.92
Transforming growth factor-beta-induced protein ig-h3	TGFBI	Q15582	10	3452.01	432.45	3890.16	794.60	3035.77	405.07	3961.39	336.63
Extracellular matrix protein 1; Isoform 4 of Extracellular matrix protein 1	ECM1	Q16610; Q16610-4	0	61453.32	7186.41	54584.20	5097.56	67978.97	12897.30	59259.85	3716.48
Putative hydroxypyruvate isomerase ;Isoform 2 of Putative hydroxypyruvate isomerase ;Isoform 3 of Putative hydroxypyruvate isomerase ;Isoform 4 of Putative hydroxypyruvate isomerase	HYI	Q5T013; Q5T013-2; Q5T013-3; Q5T013-4	0	424939.56	77504.80	403495.81	109066.65	445311.12	112291.87	356277.55	39035.40
Plexin domain-containing protein 2; Isoform 2 of Plexin domain-containing protein 2	PLXDC2	Q6UX71-1; Q6UX71-2	26.25	4081.43	649.46	4472.13	962.31	3710.26	894.71	3620.76	475.56
Peptidase inhibitor 16; Isoform 2 of Peptidase inhibitor 16	PI16	Q6UXB8-1; Q6UXB8-2	8.75	12868.68	2170.60	12943.23	2706.05	12797.85	3398.99	9063.46	1565.06

Protein Name	Gene name	UniProt ID		All ME/CFS		ME/CFS with sr-IBS		ME/CFS without sr-IBS		Control	
			Undetectable values (%)	Mean	SEM	Mean	SEM	Mean	SEM	Mean	SEM
Fermitin family homolog 3; Isoform 2 of Fermitin family homolog 3	FERMT3	Q86UX7; Q86UX7-2	41.25	10036.41	1438.72	9358.55	1865.64	10680.38	2195.26	7231.96	1149.22
Isoform 2 of Transmembrane protease serine 6; Transmembrane protease serine 6; Isoform 4 of Transmembrane protease serine 6	TMPRSS6	Q8IU80-1; Q8IU80-4; Q8IU80-5	63.75	3231.01	538.77	4116.11	816.33	2390.16	681.38	1882.94	330.23
InaD-like protein; Isoform 2 of InaD-like protein; Isoform 3 of InaD-like protein; Isoform 4 of InaD-like protein; Isoform 5 of InaD-like protein	PATJ	Q8NI35; Q8NI35-2; Q8NI35-3; Q8NI35-4; Q8NI35-5	2.5	15591.25	1326.29	14983.09	1898.89	16169.00	1889.77	17413.26	1135.68
SUN domain-containing protein 3; Isoform 2 of SUN domain-containing protein 3; Isoform 3 of SUN domain-containing protein 3	SUN3	Q8TAQ9-1; Q8TAQ9-2; Q8TAQ9-3	6.25	42376.56	4483.36	42918.39	5889.10	41861.83	6816.85	39623.17	3613.22
Complement factor H-related protein 4; Isoform 2 of Complement factor H-related protein 4	CFHR4	Q92496; Q92496-2	1.25	25248.35	3444.80	28189.11	5567.88	22454.62	4254.30	19941.80	2211.35
Proteoglycan 4; Isoform C of Proteoglycan 4; Isoform F of Proteoglycan 4	PRG4	Q92954-1; Q92954-3; Q92954-6	0	12322.42	1310.44	15553.13	2317.90	9253.24	1022.94	11404.96	1049.43
Carboxypeptidase B2	CPB2	Q96IY4	0	20639.88	1281.44	18640.74	1919.84	22539.06	1653.82	17343.58	855.50
Beta-Ala-His dipeptidase	CNDP1	Q96KN2	11.25	13696.51	2610.19	15510.58	4418.00	11973.14	3024.95	8471.31	2446.53
Fc receptor-like protein 3; Isoform 2 of Fc receptor-like protein 3; Isoform 3 of Fc receptor-like protein 3; Isoform 4 of Fc receptor-like protein 3; Isoform 5 of Fc receptor-like protein 3; Isoform 6 of Fc receptor-like protein 3; Isoform 7 of Fc receptor-like protein 3	FCRL3	Q96P31-1; Q96P31-2; Q96P31-3; Q96P31-4; Q96P31-5; Q96P31-6; Q96P31-7	30	6962.64	1567.51	8323.80	2622.96	5669.53	1835.08	15926.22	2977.23
N-acetylmuramoyl-L-alanine amidase; Isoform 2 of N-acetylmuramoyl-L-alanine amidase	PGLYRP2	Q96PD5; Q96PD5-2	0	166005.35	15039.83	168825.40	20567.97	163326.30	22268.76	144712.60	8213.60
Protein MENT	MENT	Q9BUN1	23.75	2600.62	390.24	2886.57	557.95	2328.97	552.49	2939.49	438.26
Collectin-11; Isoform 10 of Collectin-11; Isoform 2 of Collectin-11; Isoform 3 of Collectin-11; Isoform 4 of Collectin-11; Isoform 5 of Collectin-11; Isoform 6 of Collectin-11; Isoform 7 of Collectin-11; Isoform 8 of Collectin-11; Isoform 9 of Collectin-11	COLEC11	Q9BWP8; Q9BWP8-10; Q9BWP8-2; Q9BWP8-3; Q9BWP8-4; Q9BWP8-5; Q9BWP8-6; Q9BWP8-7; Q9BWP8-8; Q9BWP8-9	42.5	1610.15	316.82	1785.61	468.43	1443.47	437.93	2467.20	490.92
Complement factor H-related protein 5	CFHR5	Q9BXR6	3.75	26913.46	8675.71	38157.06	17752.47	16232.05	3451.98	14143.04	2041.45
SH3 domain-binding glutamic acid-rich-like protein 3	SH3BGRL3	Q9H299	15	20249.50	3049.89	22561.91	4991.82	18052.71	3717.34	21648.25	2303.35
cartilage acidic protein 1; Isoform 2 of Cartilage acidic protein 1; Isoform 3 of Cartilage acidic protein 1	CRTAC1	Q9NQ79; Q9NQ79-2; Q9NQ79-3	5	7992.28	626.92	8351.92	765.92	7650.62	985.62	11562.61	789.02
Complement C1r subcomponent-like protein	C1RL	Q9NZP8	0	15397.19	2243.63	19760.69	4375.69	11251.87	1175.40	13626.86	1229.09

Protein Name	Gene name	UniProt ID		All ME/CFS		ME/CFS with sr-IBS		ME/CFS without sr-IBS		Control	
			Undetectable values (%)	Mean	SEM	Mean	SEM	Mean	SEM	Mean	SEM
Fetuin-B	FETUB	Q9UGM5-1	15	13090.05	1891.76	11988.58	2254.91	14136.45	3008.48	14423.81	2090.71
Protein Z-dependent protease inhibitor	SERPINA10	Q9UK55	15	8103.15	914.29	8590.30	1341.15	7640.37	1273.67	8449.45	831.52
Talin-1	TLN1	Q9Y490	1.25	45812.00	5569.69	57668.53	9386.54	34548.29	5533.85	47050.34	4513.55
FERM, RhoGEF and pleckstrin domain-containing protein 1; Isoform 2 of FERM, RhoGEF and pleckstrin domain-containing protein 1	FARP1	Q9Y4F1; Q9Y4F1-2	92.5	536.36	242.10	286.83	0.00	773.42	460.34	4153.25	2114.24
Isoform 2 of Protocadherin gamma-C5	PCDHGC5	Q9Y5F6-2	73.75	2873.21	838.32	2656.66	880.81	3078.93	1405.69	2444.73	486.08
IgGFc-binding protein	FCGBP	Q9Y6R7	13.75	9081.57	1456.91	8213.24	1853.60	9906.48	2241.40	5799.55	1147.66
