# Supplementary material for: Plasma proteomic profiling suggests an association between antigen driven clonal B cell expansion and ME/CFS
Source: PLoS One. 2020 Jul 21;15(7):e0236148. doi: 10.1371/journal.pone.0236148 (PMC7373296; doi:10.1371/journal.pone.0236148)
Supplement: S2 Table — The aOR, 95% confidence levels and p-values from the logistic regression model in which only the linear term of the protein levels was fitted as an independent variable are shown. Quadratic effect p-value corresponds to the likelihood ratio tests comparing the goodness-of-fit of the model with both linear and quadratic terms of the protein levels to the goodness-of-fit of the model with only the linear term. aOR: adjusted odds ratio, CI: confidence interval. 1Quadratic effect p-value: crude p-value of the likelihood ratio test comparing the goodness-of-fit between the logistic regression model with both linear and quadratic terms of the protein level and the model with only the linear term. Hochberg step-up procedure was applied to correct for the multiple tests over the annotated proteins controlling the family-wise error rate (FWER) at the level of 0.05. (PDF) [file pone.0236148.s004.pdf]

Supplemental Table 2. Statistical analysis of protein association with ME/CFS.

Gene name	UniProt ID	Linear		Quadratic effect
		aOR (95% CI)	p-value	p-value[1]
IGLV4-69	A0A075B6H9	0.735 (0.391 - 1.382)	0.340	0.794
IGLV8-61	A0A075B6I0	1.173 (0.627 - 2.194)	0.619	0.132
IGLV4-60	A0A075B6I1	1.174 (0.681 - 2.023)	0.565	0.269
IGLV2-18	A0A075B6J9	0.977 (0.493 - 1.937)	0.946	0.505
IGLV3-10	A0A075B6K4	0.862 (0.514 - 1.444)	0.572	0.023
IGKV2D-30	A0A075B6S6	1.292 (0.735 - 2.27)	0.373	0.124
IGKV3D-15	A0A087WSY6	0.822 (0.442 - 1.53)	0.536	0.514
IGKV3D-11; IGKV3-11	A0A0A0MRZ8; P04433	1.05 (0.67 - 1.644)	0.833	0.000
IGHV3-49	A0A0A0MS15	1.271 (0.824 - 1.961)	0.277	0.043
IGKV6D-21	A0A0A0MT36	0.734 (0.421 - 1.282)	0.277	0.066
IGHV6-1	A0A0B4J1U7	0.752 (0.374 - 1.51)	0.423	0.617
IGHV3-15	A0A0B4J1V0	0.644 (0.338 - 1.228)	0.182	0.513
IGHV2-26	A0A0B4J1V2	1.334 (0.753 - 2.364)	0.324	0.042
IGHV3-74	A0A0B4J1X5	1.816 (1.004 - 3.283)	0.048	0.635
IGHV3-72	A0A0B4J1Y9	1.536 (0.746 - 3.162)	0.244	0.017
IGHV1D-13	A0A0B4J2D9	1.21 (0.619 - 2.364)	0.577	0.989
IGHV1-69D; IGHV1-69	A0A0B4J2H0; P01742	1.488 (0.815 - 2.714)	0.195	0.418
IGKV6-21	A0A0C4DH24	2.18 (1.022 - 4.653)	0.044	0.890
IGKV3D-20	A0A0C4DH25	1.755 (0.912 - 3.378)	0.092	0.001
IGHV1-18	A0A0C4DH31	1.148 (0.557 - 2.366)	0.708	0.467
IGHV5-51	A0A0C4DH38	1.525 (0.787 - 2.956)	0.211	0.140
IGHV4-61; IGHV4-39; IGHV 4-59; IGHV 4-34; IGHV 4-30-4; IGHV 4-38-2	A0A0C4DH41; P01824; P01825; P06331; P0DP06; P0DP08	1.387 (0.81 - 2.377)	0.234	0.047
IGKV1-8	A0A0C4DH67	1.141 (0.587 - 2.219)	0.697	0.801
IGKV2-24	A0A0C4DH68	1.299 (0.671 - 2.512)	0.438	0.651
IGKV1-12; IGKV1D-12; IGKV1D-39	A0A0C4DH73; P01611; P04432	1.097 (0.583 - 2.067)	0.774	0.088
IGHV50-10-1	A0A0J9YXX1	0.924 (0.462 - 1.849)	0.824	0.744
IGLC7	A0M8Q6	0.405 (0.192 - 0.855)	0.018	0.127
MASP2	O00187-1; O00187-2	0.725 (0.433 - 1.214)	0.222	0.166
QSOX1	O00391; O00391-2	1.27 (0.643 - 2.508)	0.492	0.426
NRP1	O14786-1;O14786-2;O14786-3	1.099 (0.703 - 1.718)	0.679	0.157
APOL1	O14791; O14791-2; O14791-3	1.564 (0.916 - 2.672)	0.101	0.876
CEP290	O15078-1; O15078-2	0.651 (0.307 - 1.38)	0.263	0.927
CD5L	O43866	1.618 (0.808 - 3.24)	0.175	0.708
FCN3	O75636-1	1.553 (0.794 - 3.036)	0.198	0.171
ATRN	O75882-1;O75882-2;O75882-3	1.611 (0.823 - 3.154)	0.164	0.846
APOM	O95445-1; O95445-2	0.787 (0.46 - 1.345)	0.381	0.029
CP	P00450	0.8 (0.443 - 1.444)	0.459	0.907
F13A1	P00488	0.915 (0.479 - 1.747)	0.787	0.392
F2	P00734	1.406 (0.787 - 2.51)	0.250	0.114
C1R	P00736	1.377 (0.769 - 2.466)	0.281	0.490
HP	P00738	0.695 (0.417 - 1.158)	0.162	0.132
HPR	P00739-1; P00739-2	0.963 (0.584 - 1.587)	0.881	0.214
F9	P00740	0.983 (0.505 - 1.912)	0.959	0.814
F10	P00742	1.304 (0.691 - 2.461)	0.413	0.326
CFD	P00746	0.809 (0.412 - 1.588)	0.537	0.013
PLG	P00747	1.03 (0.577 - 1.836)	0.921	0.806
F12	P00748	1.274 (0.717 - 2.262)	0.409	0.306
CFB	P00751-1	0.831 (0.438 - 1.579)	0.572	0.729
SERPINC1	P01008	1.12 (0.655 - 1.915)	0.680	0.028
SERPINA1	P01009-1	0.517 (0.195 - 1.369)	0.184	0.282
SERPINA3	P01011-1	1.316 (0.86 - 2.013)	0.206	0.133
AGT	P01019	1.216 (0.706 - 2.094)	0.480	0.248
A2M	P01023	0.85 (0.553 - 1.305)	0.457	0.171
C3	P01024	0.844 (0.611 - 1.166)	0.304	0.277
C5	P01031	1.017 (0.634 - 1.63)	0.945	0.031
CST3	P01034	1.293 (0.693 - 2.413)	0.419	0.448
KNG1	P01042	3.387 (1.117 - 10.27)	0.031	0.390
Isoform LMW KNG1	P01042-2	0.525 (0.266 - 1.035)	0.063	0.728
IGF2	P01344; P01344-2; P01344-3	1.057 (0.622 - 1.797)	0.836	0.142
JCHAIN	P01591	1.299 (0.713 - 2.366)	0.392	0.078
IGKV1-33	P01594	1.227 (0.619 - 2.432)	0.558	0.043
IGKV1-17	P01599	1.398 (0.839 - 2.33)	0.198	0.006
IGKV1D-16	P01601	1.37 (0.702 - 2.671)	0.356	0.361
IGKV1-5	P01602	1.277 (0.771 - 2.113)	0.342	0.324

Gene name	UniProt ID	Linear		Quadratic effect
		aOR (95% CI)	p-value	p-value[1]
IGKV2D-40	P01614	1.359 (0.671 - 2.755)	0.394	0.285
IGKV3-20	P01619	1.374 (0.761 - 2.483)	0.292	0.017
IGKV3-15	P01624	0.552 (0.305 - 0.998)	0.049	0.236
IGLV1-44	P01699	0.974 (0.491 - 1.933)	0.941	0.869
IGLV1-47	P01700	0.618 (0.358 - 1.067)	0.084	0.441
IGLV1-51	P01701	0.879 (0.461 - 1.678)	0.697	0.546
IGLV2-23	P01705	1.112 (0.574 - 2.154)	0.752	0.249
IGLV2-11	P01706	0.982 (0.524 - 1.842)	0.955	0.225
IGLV2-8	P01709	1.283 (0.657 - 2.508)	0.465	0.064
IGLV3-19	P01714	1.074 (0.558 - 2.069)	0.830	0.041
IGLV3-27	P01718	0.995 (0.569 - 1.741)	0.987	0.027
IGHV3-23; IGHV3-30	P01764; P01768	0.858 (0.568 - 1.297)	0.468	**0.000**
IGHV3-13	P01766	1.255 (0.652 - 2.417)	0.497	0.196
IGHV3-7	P01780	0.902 (0.55 - 1.479)	0.682	0.131
IGHV3-9	P01782	1.775 (0.887 - 3.553)	0.105	0.549
PIGR	P01833	2.289 (1.199 - 4.373)	0.012	0.370
IGKC	P01834	0.893 (0.55 - 1.451)	0.648	0.044
IGHG2	P01859	1.269 (0.648 - 2.484)	0.488	0.005
IGHG3	P01860	1.011 (0.514 - 1.988)	0.975	0.167
IGHG4	P01861	0.989 (0.58 - 1.686)	0.968	0.026
IGHM	P01871; P01871-2	1.301 (0.722 - 2.342)	0.381	0.655
IGHA1	P01876	0.89 (0.504 - 1.572)	0.688	0.219
IGHA2	P01877	1.498 (0.814 - 2.759)	0.194	0.574
KRT14; KRT16	P02533; P08779	1.13 (0.694 - 1.84)	0.624	0.434
APOA1	P02647	0.757 (0.48 - 1.194)	0.231	0.064
APOE	P02649	0.749 (0.41 - 1.368)	0.347	0.292
APOA2	P02652	0.8 (0.471 - 1.358)	0.408	0.133
APOC1	P02654	1.715 (0.858 - 3.427)	0.127	0.918
APOC2	P02655	1.011 (0.792 - 1.292)	0.929	0.004
APOC3	P02656	0.978 (0.522 - 1.834)	0.945	0.259
FGA	P02671-1	1.705 (0.752 - 3.866)	0.202	0.259
FGB	P02675	1.112 (0.649 - 1.906)	0.698	0.026
FGG	P02679; P02679-2	0.82 (0.567 - 1.184)	0.289	0.453
APCS	P02743	1.473 (0.792 - 2.739)	0.221	0.455
C1QA	P02745	0.737 (0.399 - 1.361)	0.329	0.019
C1QB	P02746	0.911 (0.493 - 1.683)	0.765	0.395
C1QC	P02747	1.027 (0.541 - 1.949)	0.935	0.380
C9	P02748	1.495 (0.714 - 3.129)	0.286	0.346
APOH	P02749	1.529 (0.78 - 3)	0.217	0.887
LRG1	P02750	0.788 (0.429 - 1.45)	0.444	0.611
FN1	P02751; P02751-11; P02751-14; P02751-15; P02751-3; P02751-7; P02751-8	1.032 (0.538 - 1.98)	0.923	0.646
RBP4	P02753	0.64 (0.347 - 1.181)	0.154	0.501
AMBP	P02760	0.978 (0.505 - 1.894)	0.947	0.127
ORM1	P02763	1.03 (0.471 - 2.252)	0.941	0.146
AHSG	P02765	1.202 (0.81 - 1.784)	0.360	0.031
TTR	P02766	0.768 (0.446 - 1.321)	0.340	0.025
ALB	P02768-1	0.895 (0.688 - 1.166)	0.411	0.035
GC	P02774; P02774-3	0.738 (0.462 - 1.18)	0.205	0.802
PPBP	P02775	1.162 (0.738 - 1.829)	0.517	0.054
PF4	P02776	1.045 (0.762 - 1.434)	0.783	0.027
TF	P02787	0.815 (0.476 - 1.396)	0.456	0.169
HPX	P02790	0.558 (0.283 - 1.099)	0.092	0.881
ANG	P03950	1.793 (0.933 - 3.446)	0.080	0.025
F11	P03951; P03951-2	1.379 (0.853 - 2.227)	0.190	0.138
KLKB1	P03952	1.31 (0.821 - 2.09)	0.257	0.104
C4BPA	P04003	0.876 (0.619 - 1.238)	0.452	0.159
VTN	P04004	0.913 (0.668 - 1.249)	0.570	0.082
PROC	P04070; P04070-2	1.211 (0.712 - 2.06)	0.480	0.169
ALDOA	P04075; P04075-2	0.814 (0.455 - 1.456)	0.488	0.866
APOB	P04114	2.256 (1.081 - 4.71)	0.030	0.589
LCAT	P04180	1.134 (0.639 - 2.012)	0.667	0.301
HRG	P04196	1.285 (0.695 - 2.376)	0.425	0.184
IGLV7-43	P04211	1.146 (0.626 - 2.098)	0.659	0.526
A1BG	P04217	0.733 (0.45 - 1.195)	0.213	0.426
KRT1	P04264	1.13 (0.609 - 2.097)	0.698	0.272

Gene name	UniProt ID	Linear		Quadratic effect
		aOR (95% CI)	p-value	p-value[1]
VWF	P04275	0.68 (0.333 - 1.387)	0.289	0.956
SHBG	P04278-1	0.679 (0.393 - 1.172)	0.165	0.260
IGF1	P05019; P05019-2; P05019-3; P05019-4	1.013 (0.643 - 1.596)	0.956	0.029
ALDOB	P05062	1.467 (0.729 - 2.952)	0.283	0.186
APOD	P05090	0.918 (0.488 - 1.729)	0.792	0.566
SERPINA5	P05154	0.949 (0.495 - 1.817)	0.873	0.440
SERPING1	P05155; P05155-2; P05155-3	0.798 (0.527 - 1.21)	0.289	0.521
CFI	P05156	0.826 (0.443 - 1.539)	0.547	0.202
F13B	P05160	0.773 (0.418 - 1.431)	0.413	0.997
CLEC3B	P05452	1.205 (0.623 - 2.329)	0.580	0.561
SERPINA7	P05543	1.053 (0.56 - 1.978)	0.873	0.453
SERPIND1	P05546	0.65 (0.342 - 1.234)	0.188	0.159
IGKV4-1	P06312	1.071 (0.63 - 1.819)	0.801	0.010
GSN	P06396	0.668 (0.304 - 1.468)	0.315	0.003
ATP5F1B	P06576	1.038 (0.569 - 1.894)	0.904	0.156
C2	P06681-1	1.111 (0.62 - 1.99)	0.724	0.844
APOA4	P06727	0.584 (0.335 - 1.019)	0.058	0.432
PROS1	P07225	1.276 (0.742 - 2.196)	0.378	0.432
C8A	P07357	1.258 (0.673 - 2.355)	0.472	0.257
C8B	P07358	0.878 (0.537 - 1.437)	0.606	0.030
C8G	P07360	1.688 (0.912 - 3.123)	0.096	0.734
PFN1	P07737	0.695 (0.409 - 1.182)	0.179	0.034
THBS1	P07996; P07996-2	0.691 (0.397 - 1.203)	0.191	0.044
SERPINA6	P08185	0.878 (0.425 - 1.813)	0.724	0.652
LPA	P08519	1.124 (0.532 - 2.373)	0.760	0.384
PLEK	P08567	0.786 (0.444 - 1.393)	0.410	0.172
CD14	P08571	0.86 (0.475 - 1.556)	0.618	0.516
CFH	P08603-1	0.863 (0.518 - 1.436)	0.570	0.102
FCGR3A	P08637	1.706 (0.781 - 3.727)	0.181	0.325
SERPINF2	P08697-1	1.569 (0.92 - 2.676)	0.098	0.111
C1S	P09871	1.006 (0.591 - 1.712)	0.981	0.039
C4A	P0C0L4-1	0.966 (0.671 - 1.39)	0.852	0.010
C4B	P0C0L5	1.363 (0.788 - 2.358)	0.268	0.527
SAA1	P0DJI8	0.778 (0.404 - 1.5)	0.454	0.740
IGHA2	P0DOX2	1.253 (0.639 - 2.455)	0.511	0.701
IGD	P0DOX3	1.179 (0.657 - 2.114)	0.581	0.004
IGG1	P0DOX5	0.686 (0.37 - 1.273)	0.232	0.126
IGM	P0DOX6	1.523 (0.809 - 2.867)	0.193	0.042
IGL	P0DOX7	1.327 (0.608 - 2.895)	0.478	0.130
IGK	P0DOX8	0.768 (0.474 - 1.242)	0.282	0.011
IGLC3	P0DOY3	1.084 (0.567 - 2.07)	0.808	0.707
C7	P10643	0.914 (0.52 - 1.605)	0.753	0.305
CLU	P10909-1; P10909-2; P10909-4; P10909-5	0.647 (0.363 - 1.153)	0.139	0.429
HSPA8; HSPA2	P11142-1; P11142-2; P54652	0.579 (0.306 - 1.097)	0.094	0.607
MBL2	P11226	1.02 (0.512 - 2.035)	0.954	0.532
CETP	P11597-1; P11597-2	0.519 (0.281 - 0.959)	0.036	0.611
F5	P12259	2.902 (1.23 - 6.849)	0.015	0.441
KRT10	P13645	0.745 (0.379 - 1.463)	0.393	0.133
C6	P13671	0.667 (0.394 - 1.132)	0.133	0.994
SELL	P14151; P14151-2	1.112 (0.596 - 2.076)	0.739	0.257
PKM	P14618	0.766 (0.396 - 1.481)	0.428	0.257
PVR	P15151-1; P15151-2; P15151-3; P15151-4	0.6 (0.329 - 1.092)	0.095	0.014
RAC2; RAC3; RAC1	P15153; P60763; P63000-1; P63000-2	1.24 (0.777 - 1.979)	0.366	0.677
CPN1	P15169	1.279 (0.625 - 2.618)	0.501	0.203
IGLL1	P15814	0.762 (0.405 - 1.433)	0.399	0.462
CD44	P16070; P16070-10; P16070-11; P16070-12; P16070-13; P16070-14; P16070-15; P16070-16; P16070-17; P16070-18; P16070-3; P16070-4; P16070-5; P16070-6; P16070-7; P16070-8; P16070-9	1.112 (0.555 - 2.227)	0.765	0.631
HSPA6; HSPA7	P17066; P48741	1.237 (0.592 - 2.582)	0.572	0.926
IGFBP3	P17936; P17936-2	0.69 (0.366 - 1.3)	0.251	0.103
LBP	P18428	1.709 (0.95 - 3.075)	0.074	0.221
ORM2	P19652	0.877 (0.423 - 1.818)	0.724	0.721
ITIH2	P19823	0.607 (0.328 - 1.12)	0.110	0.801
ITIH1	P19827-1	1.316 (0.722 - 2.4)	0.370	0.079

Gene name	UniProt ID	Linear		Quadratic effect
		aOR (95% CI)	p-value	p-value[1]
PZP	P20742	0.828 (0.524 - 1.307)	0.417	0.002
C4BPB	P20851; P20851-2	1.327 (0.646 - 2.725)	0.441	0.564
FLNA	P21333; P21333-2	1.107 (0.595 - 2.059)	0.749	0.480
GPX3	P22352	0.684 (0.366 - 1.278)	0.233	0.871
CPN2	P22792	1.067 (0.605 - 1.881)	0.822	0.676
PROZ	P22891-1; P22891-2	1.099 (0.606 - 1.994)	0.756	0.960
IGHV1-2	P23083	1.212 (0.594 - 2.473)	0.597	0.625
FBLN1	P23142	0.907 (0.483 - 1.703)	0.762	0.912
FBLN1	P23142-4	0.783 (0.442 - 1.388)	0.402	0.172
CFL1	P23528	0.951 (0.494 - 1.83)	0.880	0.106
IGFBP5	P24593	1.728 (0.906 - 3.296)	0.097	0.534
AZGP1	P25311	1.2 (0.582 - 2.475)	0.622	0.077
MST1	P26927	1.157 (0.723 - 1.853)	0.544	0.012
PON1	P27169	0.895 (0.601 - 1.332)	0.585	0.098
CFP	P27918	0.939 (0.568 - 1.552)	0.806	0.214
SERPINA4	P29622	0.973 (0.6 - 1.579)	0.912	0.026
PRDX6	P30041	1.741 (0.858 - 3.53)	0.124	0.191
PDIA3	P30101	1.711 (0.819 - 3.573)	0.153	0.821
KRT9	P35527	1.016 (0.528 - 1.958)	0.961	0.196
SAA4	P35542	0.724 (0.375 - 1.397)	0.336	0.449
IGFALS	P35858; P35858-2	0.56 (0.305 - 1.027)	0.061	0.094
KRT2	P35908	1.4 (0.713 - 2.75)	0.329	0.416
SERPINF1	P36955	0.79 (0.473 - 1.321)	0.369	0.101
CFHR2	P36980-1	0.772 (0.428 - 1.392)	0.390	0.294
PTGDS	P41222	1.133 (0.672 - 1.909)	0.639	0.458
BTD	P43251; P43251-2; P43251-3; P43251-4	0.769 (0.438 - 1.349)	0.359	0.067
AFM	P43652	0.85 (0.567 - 1.274)	0.431	0.021
MASP1	P48740-1	0.838 (0.46 - 1.524)	0.562	0.789
MASP1	P48740-2; P48740-4	1.036 (0.572 - 1.877)	0.907	0.224
SELENOP	P49908	0.882 (0.556 - 1.398)	0.592	0.010
CAMP	P49913	1.943 (0.962 - 3.926)	0.064	0.319
LUM	P51884	0.704 (0.405 - 1.221)	0.211	0.029
CRISP3	P54108-1; P54108-2; P54108-3	1.83 (0.941 - 3.56)	0.075	0.339
APOC4	P55056	0.631 (0.273 - 1.459)	0.281	0.733
PLTP	P55058	0.878 (0.469 - 1.642)	0.683	0.270
CDH13	P55290; P55290-4	1.315 (0.733 - 2.357)	0.358	0.336
DEFA1; DEFA1; DEFA1B	P59665; P59666	0.977 (0.524 - 1.821)	0.941	0.173
ACTB; ACTG1	P60709; P63261	0.739 (0.431 - 1.266)	0.271	0.632
RAP1A; RAP1B	P61224-1; P61224-2; P61224-3; P61224-4; P62834	0.71 (0.328 - 1.538)	0.385	0.458
B2M	P61769	0.927 (0.506 - 1.697)	0.805	0.119
PPIA	P62937	1.579 (0.768 - 3.245)	0.214	0.548
YWHAZ	P63104-1	1.502 (0.802 - 2.814)	0.204	0.195
TUBA1B; TUBA1A; TUBA1C	P68363; P68363-2; Q71U36; Q71U36-2; Q9BQE3	0.481 (0.209 - 1.107)	0.085	0.040
HBB	P68871	1.105 (0.618 - 1.976)	0.735	0.139
HBA1	P69905	2.131 (0.864 - 5.254)	0.100	0.091
GPLD1	P80108	1.16 (0.624 - 2.156)	0.638	0.106
IGLV3-21	P80748	0.765 (0.358 - 1.635)	0.490	0.523
CFHR3	Q02985-1; Q02985-2	0.984 (0.547 - 1.77)	0.957	0.445
CFHR1	Q03591	0.764 (0.412 - 1.415)	0.392	0.762
HGFAC	Q04756	0.796 (0.457 - 1.386)	0.420	0.760
ITIH3	Q06033-1; Q06033-2	1.466 (0.727 - 2.957)	0.286	0.587
PRDX1	Q06830	1.282 (0.662 - 2.484)	0.461	0.891
POLE	Q07864	1.042 (0.577 - 1.885)	0.891	0.378
LGALS3BP	Q08380	0.779 (0.465 - 1.306)	0.344	0.065
EFEMP1	Q12805; Q12805-2; Q12805-3; Q12805-4; Q12805-5	1.162 (0.583 - 2.317)	0.670	0.661
CTTN	Q14247-1; Q14247-2; Q14247-3	1.272 (0.805 - 2.009)	0.302	0.563
HABP2	Q14520-1; Q14520-2	1.286 (0.721 - 2.295)	0.394	0.127
ITIH4	Q14624-1	0.824 (0.56 - 1.211)	0.325	0.001
ITIH4	Q14624-2; Q14624-3	1.024 (0.511 - 2.053)	0.947	0.115
PCOLCE	Q15113	0.866 (0.458 - 1.637)	0.657	0.504
PON3	Q15166	1.913 (1.009 - 3.627)	0.047	0.876
TGFBI	Q15582	0.898 (0.561 - 1.437)	0.654	0.108
ECM1	Q16610; Q16610-4	0.86 (0.534 - 1.384)	0.534	0.082

Gene name	UniProt ID	Linear		Quadratic effect
		aOR (95% CI)	p-value	p-value[1]
HYI	Q5T013; Q5T013-2; Q5T013-3; Q5T013-4	0.847 (0.533 - 1.346)	0.483	0.060
PLXDC2	Q6UX71-1; Q6UX71-2	1.027 (0.572 - 1.844)	0.928	0.200
PI16	Q6UXB8-1; Q6UXB8-2	1.081 (0.599 - 1.952)	0.796	0.007
FERMT3	Q86UX7; Q86UX7-2	1.451 (0.739 - 2.849)	0.280	0.104
TMPRSS6	Q8IU80-1; Q8IU80-4; Q8IU80-5	1.372 (0.809 - 2.325)	0.240	0.055
PATJ	Q8NI35; Q8NI35-2; Q8NI35-3; Q8NI35-4; Q8NI35-5	0.79 (0.511 - 1.222)	0.290	0.143
SUN3	Q8TAQ9-1; Q8TAQ9-2; Q8TAQ9-3	0.612 (0.369 - 1.015)	0.057	0.215
CFHR4	Q92496; Q92496-2	1.29 (0.668 - 2.491)	0.449	0.398
PRG4	Q92954-1; Q92954-3; Q92954-6	0.808 (0.41 - 1.591)	0.537	0.720
CPB2	Q96IY4	1.219 (0.697 - 2.132)	0.488	0.001
CNDP1	Q96KN2	1.622 (0.882 - 2.983)	0.119	0.657
FCRL3	Q96P31-1; Q96P31-2; Q96P31-3; Q96P31-4; Q96P31-5; Q96P31-6; Q96P31-7	0.353 (0.133 - 0.933)	0.036	0.145
PGLYRP2	Q96PD5; Q96PD5-2	0.966 (0.654 - 1.425)	0.861	0.031
MENT	Q9BUN1	0.973 (0.544 - 1.74)	0.926	0.549
COLEC11	Q9BWP8; Q9BWP8-10; Q9BWP8-2; Q9BWP8-3; Q9BWP8-4; Q9BWP8-5; Q9BWP8-6; Q9BWP8-7; Q9BWP8-8; Q9BWP8-9	0.644 (0.318 - 1.306)	0.222	0.675
CFHR5	Q9BXR6	1.575 (0.752 - 3.299)	0.229	0.988
SH3BGRL3	Q9H299	0.839 (0.457 - 1.54)	0.572	0.615
CRTAC1	Q9NQ79; Q9NQ79-2; Q9NQ79-3	0.607 (0.329 - 1.119)	0.109	0.994
C1RL	Q9NZP8	0.811 (0.412 - 1.595)	0.544	0.942
FETUB	Q9UGM5-1	0.697 (0.365 - 1.332)	0.275	0.722
SERPINA10	Q9UK55	0.871 (0.493 - 1.536)	0.633	0.288
TLN1	Q9Y490	0.62 (0.374 - 1.028)	0.064	0.715
FARP1	Q9Y4F1; Q9Y4F1-2	0.932 (0.467 - 1.858)	0.841	0.443
PCDHGC5	Q9Y5F6-2	1.352 (0.695 - 2.632)	0.374	0.097
FCGBP	Q9Y6R7	1.133 (0.642 - 2)	0.666	0.037
