# Supplementary material for: Plasma proteomic profiling suggests an association between antigen driven clonal B cell expansion and ME/CFS
Source: PLoS One. 2020 Jul 21;15(7):e0236148. doi: 10.1371/journal.pone.0236148 (PMC7373296; doi:10.1371/journal.pone.0236148)
Supplement: S3 Table — aOR, 95% confidence levels and p-values from the logistic regression model in which only the linear term of the protein levels was fitted as an independent variable are shown in females only. Quadratic effect p-value corresponds to the likelihood ratio tests that compare the goodness-of-fit of the model with both linear and quadratic terms of the protein levels to that of the model with only the linear term in females only. ME/CFS: myalgic encephalomyelitis/chronic fatigue syndrome, SEM: standard error of mean, aOR: adjusted odds ratio, CI: confidence interval. 1Quadratic effect p-value: crude p-value of the likelihood ratio test comparing the goodness-of-fit between the logistic regression model with both linear and quadratic terms of the protein level and the model with only the linear term. Hochberg step-up procedure was applied to correct for the multiple tests over the annotated proteins controlling the family-wise error rate (FWER) at the level of 0.05. (PDF) [file pone.0236148.s005.pdf]

Supplementary Table 3. Mean levels of proteins detected in all female ME/CFS cases versus female controls

Gene name	UniProt ID	ME/CFS		Controls		Linear		Quadratic effect
		Mean	SEM	Mean	SEM	OR (95% CI)	p-value	p-value[1]
IGLV4-69	A0A075B6H9	11977.47	7447.05	13106.86	4229.08	0.585 (0.263 - 1.305)	0.191	0.357
IGLV8-61	A0A075B6I0	31158.98	5082.32	28270.44	4790.55	1.723 (0.718 - 4.137)	0.224	0.010
IGLV4-60	A0A075B6I1	382.77	67.36	527.48	92.45	1.176 (0.655 - 2.11)	0.588	0.206
IGLV2-18	A0A075B6J9	28010.96	3799.53	57392.05	13953.43	0.784 (0.316 - 1.945)	0.599	0.366
IGLV3-10	A0A075B6K4	31472.26	4144.82	27469.45	2661.29	1.097 (0.557 - 2.161)	0.789	0.231
IGKV2D-30	A0A075B6S6	31082.24	2854.94	30713.69	2816.39	1.603 (0.721 - 3.562)	0.247	0.177
IGKV3D-15	A0A087WSY6	64650.70	10319.08	56019.93	13048.23	1.118 (0.479 - 2.609)	0.796	0.197
IGKV3D-11; IGKV3-11	A0A0A0MRZ8; P04433	1006944.21	169940.72	789064.54	64400.45	0.998 (0.616 - 1.616)	0.992	0.003
IGHV3-49	A0A0A0MS15	42082.64	4912.29	40052.50	4663.56	1.581 (0.913 - 2.737)	0.102	0.090
IGKV6D-21	A0A0A0MT36	24815.75	3663.71	21005.62	3037.39	1.03 (0.525 - 2.019)	0.932	0.092
IGHV6-1	A0A0B4J1U7	103756.94	15702.02	101231.38	9636.45	0.815 (0.38 - 1.746)	0.599	0.597
IGHV3-15	A0A0B4J1V0	19047.78	4441.12	28508.32	6688.86	0.891 (0.429 - 1.854)	0.759	0.487
IGHV2-26	A0A0B4J1V2	5918.59	555.68	5657.42	705.86	1.742 (0.81 - 3.747)	0.156	0.601
IGHV3-74	A0A0B4J1X5	790.13	251.20	595.05	122.47	1.612 (0.879 - 2.956)	0.123	0.749
IGHV3-72	A0A0B4J1Y9	129275.19	9597.50	124413.65	6968.90	1.257 (0.617 - 2.56)	0.529	0.017
IGHV1D-13	A0A0B4J2D9	11965.80	4617.83	19836.86	7939.78	1.52 (0.642 - 3.602)	0.341	0.233
IGHV1-69D; IGHV1-69	A0A0B4J2H0; P01742	32048.75	3881.40	32478.16	3227.35	2.135 (0.916 - 4.975)	0.079	0.818
IGKV6-21	A0A0C4DH24	37026.53	6588.15	33432.83	6849.83	3.533 (1.228 - 10.166)	0.019	0.659
IGKV3D-20	A0A0C4DH25	316774.01	45617.23	260622.78	31066.05	2.099 (0.956 - 4.607)	0.065	0.002
IGHV1-18	A0A0C4DH31	133932.02	18037.72	175156.96	24123.47	1.36 (0.615 - 3.007)	0.447	0.604
IGHV5-51	A0A0C4DH38	110502.44	19941.55	92117.91	14206.42	1.786 (0.873 - 3.654)	0.112	0.621
IGHV4-61; IGHV4-39; IGHV 4-59; IGHV 4-34; IGHV 4-30-4; IGHV 4-38-2	A0A0C4DH41; P01824; P01825; P06331; P0DP06; P0DP08	52230.91	5622.61	41415.37	2924.87	1.602 (0.803 - 3.199)	0.181	0.494
IGKV1-8	A0A0C4DH67	81856.19	8634.13	83276.44	11627.06	1.453 (0.684 - 3.089)	0.331	0.864
IGKV2-24	A0A0C4DH68	16733.13	2510.62	16958.93	2769.10	2.173 (0.842 - 5.61)	0.109	0.184
IGKV1-12; IGKV1D-12; IGKV1D-39	A0A0C4DH73; P01611; P04432	153317.75	11971.46	144388.56	18970.21	1.18 (0.6 - 2.318)	0.632	0.145
IGHV50-10-1	A0A0J9YXX1	3255.70	786.96	3337.13	822.06	0.98 (0.428 - 2.247)	0.962	0.396
IGLC7	A0M8Q6	8086.33	1839.89	7413.64	1217.86	0.374 (0.147 - 0.952)	0.039	0.007
MASP2	O00187-1; O00187-2	16377.63	1525.46	15391.66	1424.20	1.07 (0.546 - 2.097)	0.844	0.187
QSOX1	O00391; O00391-2	4015.99	523.10	4341.70	636.51	1.302 (0.566 - 2.999)	0.535	0.425
NRP1	O14786-1; O14786-2; O14786-3	1607.78	444.76	2062.13	374.17	1.06 (0.656 - 1.711)	0.813	0.081
APOL1	O14791; O14791-2; O14791-3	15844.73	2653.24	13507.33	1548.91	1.466 (0.739 - 2.905)	0.273	0.744
CEP290	O15078-1; O15078-2	689336.74	80593.32	768486.73	84504.02	0.67 (0.283 - 1.586)	0.363	0.863
CD5L	O43866	83984.99	8713.55	77297.74	7786.40	1.25 (0.516 - 3.027)	0.621	0.131
FCN3	O75636-1	17209.23	4347.58	11599.25	3187.48	1.217 (0.544 - 2.721)	0.633	0.291
ATRN	O75882-1; O75882-2; O75882-3	19693.90	794.48	19899.61	1095.84	1.528 (0.736 - 3.172)	0.256	0.815
APOM	O95445-1; O95445-2	35120.21	4073.24	35777.98	3564.37	0.762 (0.4 - 1.452)	0.409	0.583
CP	P00450	910964.82	53220.54	978204.62	46624.95	0.737 (0.372 - 1.461)	0.383	0.956
F13A1	P00488	14826.70	1426.48	14770.18	1644.32	1.085 (0.539 - 2.184)	0.820	0.340
F2	P00734	1224782.91	174975.68	1069467.77	124478.49	1.383 (0.73 - 2.617)	0.320	0.262
C1R	P00736	47167.93	3037.69	44678.70	2905.55	1.256 (0.684 - 2.305)	0.463	0.580
HP	P00738	5698694.78	685472.18	5553579.93	476756.19	0.735 (0.43 - 1.254)	0.259	0.083
HPR	P00739-1; P00739-2	100823.72	24423.53	80209.83	8920.27	0.764 (0.412 - 1.417)	0.393	0.314
F9	P00740	56349.44	10431.89	48308.76	8152.30	0.974 (0.478 - 1.982)	0.941	0.858
F10	P00742	71331.92	7240.73	70837.31	6662.43	1.183 (0.599 - 2.335)	0.629	0.621
CFD	P00746	17955.62	1939.88	17017.57	2069.14	0.91 (0.46 - 1.802)	0.787	0.031
PLG	P00747	479369.34	36538.14	462173.61	30676.86	1.191 (0.598 - 2.371)	0.620	0.473
F12	P00748	76593.43	5474.23	73404.70	5539.55	1.207 (0.677 - 2.154)	0.524	0.278
CFB	P00751-1	352203.03	40762.97	324671.87	28700.51	0.872 (0.427 - 1.78)	0.707	0.658

Gene name	UniProt ID	ME/CFS		Controls		Linear		Quadratic effect p-value[1]
		Mean	SEM	Mean	SEM	OR (95% CI)	p-value	
SERPINC1	P01008	278871.45	16813.61	237252.57	13335.97	1.149 (0.617 - 2.138)	0.661	0.024
SERPINA1	P01009-1	284808.75	28579.05	360690.49	29917.30	0.224 (0.059 - 0.854)	0.028	0.222
SERPINA3	P01011-1	34945.75	4204.92	33944.20	2934.04	1.26 (0.798 - 1.989)	0.321	0.184
AGT	P01019	73384.53	7231.36	72268.50	7191.78	1.222 (0.681 - 2.194)	0.501	0.416
A2M	P01023	3765704.84	319870.28	3576531.55	294577.56	0.804 (0.501 - 1.291)	0.367	0.289
C3	P01024	875373.09	55871.77	786830.87	30288.44	0.904 (0.687 - 1.188)	0.469	0.042
C5	P01031	117649.69	6279.53	116838.98	5587.16	0.737 (0.402 - 1.354)	0.326	0.297
CST3	P01034	10034.63	2448.71	8653.37	2378.22	1.48 (0.702 - 3.123)	0.303	0.523
KNG1	P01042	21495.84	3645.30	20895.64	5611.92	2.936 (0.869 - 9.917)	0.083	0.106
Isoform LMW KNG1	P01042-2	784860.19	53045.70	757464.74	48082.01	0.649 (0.302 - 1.394)	0.267	0.688
IGF2	P01344; P01344-2; P01344-3	61823.90	9094.09	62154.54	10550.05	1.075 (0.579 - 1.994)	0.819	0.129
JCHAIN	P01591	444530.07	43730.99	412298.34	39226.35	1.247 (0.638 - 2.436)	0.519	0.007
IGKV1-33	P01594	313965.35	33029.76	293149.68	32424.06	1.34 (0.612 - 2.933)	0.464	0.079
IGKV1-17	P01599	23891.80	3711.78	16232.25	1451.49	2.123 (1.032 - 4.371)	0.041	0.151
IGKV1D-16	P01601	55686.62	7116.23	40940.35	6080.79	1.654 (0.683 - 4.008)	0.265	0.149
IGKV1-5	P01602	15638.18	1532.60	16630.21	1434.43	1.585 (0.797 - 3.15)	0.189	0.737
IGKV2D-40	P01614	86693.44	11952.08	76763.41	7235.29	1.579 (0.667 - 3.736)	0.299	0.167
IGKV3-20	P01619	404906.22	51196.99	342760.79	33953.34	1.498 (0.712 - 3.151)	0.287	0.042
IGKV3-15	P01624	882596.24	140551.50	1063507.39	166455.19	0.533 (0.26 - 1.092)	0.086	0.529
IGLV1-44	P01699	2235027.65	531452.89	1452559.84	443123.64	0.922 (0.395 - 2.149)	0.850	0.850
IGLV1-47	P01700	98310.08	9382.04	113359.01	9124.54	0.77 (0.425 - 1.396)	0.389	0.320
IGLV1-51	P01701	32762.41	4305.82	36198.33	5830.28	0.648 (0.238 - 1.769)	0.397	0.744
IGLV2-23	P01705	4593.96	1292.34	5289.80	1353.61	1.224 (0.516 - 2.902)	0.647	0.646
IGLV2-11	P01706	14138.77	2280.11	19718.42	3524.74	1.46 (0.651 - 3.274)	0.358	0.589
IGLV2-8	P01709	35657.14	6405.03	23989.46	3077.85	1.556 (0.646 - 3.752)	0.325	0.003
IGLV3-19	P01714	299574.83	61371.81	268612.67	33521.41	1.093 (0.527 - 2.268)	0.811	0.068
IGLV3-27	P01718	10586.00	1502.17	11114.29	1557.88	1.045 (0.555 - 1.968)	0.891	0.133
IGHV3-23; IGHV3-30	P01764; P01768	89499.40	9567.12	77386.28	5742.08	0.985 (0.59 - 1.642)	0.952	**0.000**
IGHV3-13	P01766	24418.14	3288.78	22667.30	2823.38	1.438 (0.628 - 3.291)	0.390	0.413
IGHV3-7	P01780	6619.77	582.03	6764.39	655.26	1.343 (0.697 - 2.588)	0.378	0.676
IGHV3-9	P01782	103646.69	9028.61	103244.60	9673.28	1.622 (0.765 - 3.436)	0.207	0.726
PIGR	P01833	3445.26	738.36	1524.78	548.79	2.057 (1.067 - 3.963)	0.031	0.249
IGKC	P01834	815744.32	63208.28	836430.94	81354.46	1.448 (0.725 - 2.891)	0.294	0.109
IGHG2	P01859	5751351.31	638199.37	4825995.59	455919.90	1.366 (0.656 - 2.845)	0.405	0.004
IGHG3	P01860	2199880.26	322660.81	1934682.62	229173.56	0.936 (0.444 - 1.974)	0.862	0.152
IGHG4	P01861	279631.95	43485.38	317592.10	51612.38	1.072 (0.624 - 1.841)	0.802	0.040
IGHM	P01871; P01871-2	2676043.20	294839.22	2870602.20	317951.41	1.191 (0.528 - 2.689)	0.674	0.254
IGHA1	P01876	6553305.42	788942.62	6304915.12	619035.63	0.924 (0.509 - 1.675)	0.794	0.182
IGHA2	P01877	300570.71	42923.66	241319.25	24938.09	1.586 (0.755 - 3.33)	0.223	0.832
KRT14; KRT16	P02533; P08779	1511.83	0.00	5910.86	3798.79	1.076 (0.637 - 1.816)	0.784	0.012
APOA1	P02647	4671569.33	308591.40	3991133.81	221983.06	1.033 (0.614 - 1.738)	0.901	0.009
APOE	P02649	188802.39	15501.36	156783.81	13404.32	1.032 (0.504 - 2.113)	0.931	0.292
APOA2	P02652	566271.42	54005.00	546119.52	41482.27	0.829 (0.47 - 1.46)	0.516	0.032
APOC1	P02654	107018.14	10904.64	101727.50	13500.50	2.027 (0.851 - 4.825)	0.111	0.533
APOC2	P02655	164282.65	15430.45	140238.41	9783.14	1.371 (0.933 - 2.013)	0.108	0.066
APOC3	P02656	1616395.10	129485.51	1241692.04	123880.03	1.382 (0.697 - 2.739)	0.355	0.089
FGA	P02671-1	2288756.84	227289.61	2109829.18	260117.94	1.095 (0.417 - 2.877)	0.854	0.296
FGB	P02675	2102164.99	92054.05	2196542.95	103734.48	1.105 (0.62 - 1.97)	0.735	0.046
FGG	P02679; P02679-2	988211.62	65266.52	914607.23	44316.26	0.911 (0.66 - 1.256)	0.568	0.074

Gene name	UniProt ID	ME/CFS		Controls		Linear		Quadratic effect p-value[1]
		Mean	SEM	Mean	SEM	OR (95% CI)	p-value	
APCS	P02743	9967.88	788.16	8603.64	613.41	1.961 (0.874 - 4.402)	0.103	0.082
C1QA	P02745	16895.13	3075.11	16580.00	1862.27	0.786 (0.371 - 1.666)	0.530	0.089
C1QB	P02746	83950.93	4976.13	91207.22	4901.92	0.896 (0.442 - 1.815)	0.760	0.763
C1QC	P02747	155398.94	7407.04	170540.60	10329.13	1.16 (0.567 - 2.371)	0.685	0.624
C9	P02748	56476.24	5261.34	54024.56	4165.45	1.362 (0.596 - 3.113)	0.464	0.674
APOH	P02749	505736.86	66716.64	401141.77	42028.89	1.612 (0.718 - 3.618)	0.247	0.678
LRG1	P02750	162008.51	14974.64	218833.63	16565.91	0.539 (0.256 - 1.136)	0.104	0.616
FN1	P02751; P02751-11; P02751-14; P02751-15; P02751-3; P02751-7; P02751-8	531009.39	61857.99	513549.81	64566.66	1.212 (0.6 - 2.445)	0.592	0.317
RBP4	P02753	218505.74	23030.19	218968.17	21944.61	0.628 (0.313 - 1.261)	0.191	0.852
AMBP	P02760	1786710.79	168718.86	1701797.58	153627.86	1.134 (0.53 - 2.426)	0.746	0.082
ORM1	P02763	119479.70	11881.87	148529.76	20595.07	0.959 (0.411 - 2.238)	0.924	0.091
AHSG	P02765	1654067.09	201763.65	1637535.57	179676.51	1.163 (0.771 - 1.757)	0.472	0.081
TTR	P02766	367725.43	26559.70	377543.80	25369.80	1.059 (0.535 - 2.095)	0.870	0.052
ALB	P02768-1	80443943.20	5366074.19	76299276.63	4543870.93	0.906 (0.692 - 1.185)	0.471	0.003
GC	P02774; P02774-3	946007.19	87957.43	1058017.22	74359.78	0.62 (0.323 - 1.19)	0.151	0.806
PPBP	P02775	271155.37	30004.30	258407.30	26434.29	1.358 (0.776 - 2.377)	0.284	0.159
PF4	P02776	21278.19	1782.35	20771.25	1811.22	1.323 (0.771 - 2.271)	0.310	0.528
TF	P02787	8227498.74	902202.43	7610427.63	708331.68	0.745 (0.405 - 1.371)	0.344	0.406
HPX	P02790	4230868.96	233950.08	4303507.00	244040.63	0.686 (0.298 - 1.577)	0.374	0.988
ANG	P03950	10359.48	4288.64	5623.47	1346.41	1.527 (0.671 - 3.474)	0.313	0.022
F11	P03951; P03951-2	12787.45	2135.31	9915.64	821.15	1.295 (0.774 - 2.168)	0.325	0.268
KLKB1	P03952	116213.36	8631.71	125997.10	7736.20	0.985 (0.571 - 1.698)	0.957	0.053
C4BPA	P04003	210495.73	20676.23	201566.84	13989.13	0.805 (0.521 - 1.245)	0.330	0.636
VTN	P04004	1083403.59	101807.68	935046.59	82422.51	0.853 (0.586 - 1.242)	0.406	0.278
PROC	P04070; P04070-2	5078.69	502.15	5110.92	443.30	1.06 (0.585 - 1.923)	0.847	0.192
ALDOA	P04075; P04075-2	12155.28	1296.44	10819.59	882.24	1.094 (0.506 - 2.365)	0.819	0.299
APOB	P04114	189588.84	14048.52	173674.68	11828.44	3.281 (1.084 - 9.931)	0.036	0.682
LCAT	P04180	8604.65	905.36	8717.64	792.03	0.948 (0.468 - 1.921)	0.883	0.916
HRG	P04196	394804.07	105146.25	305594.47	37370.05	1.246 (0.641 - 2.42)	0.516	0.504
IGLV7-43	P04211	9039.10	4691.53	3324.04	442.89	1.714 (0.711 - 4.134)	0.230	0.391
A1BG	P04217	1500149.70	174131.77	1471828.30	141280.11	0.757 (0.448 - 1.278)	0.297	0.315
KRT1	P04264	5162.68	1109.80	8795.76	2697.33	0.839 (0.411 - 1.714)	0.630	0.884
VWF	P04275	10799.67	987.84	13653.96	1516.13	0.752 (0.305 - 1.853)	0.535	0.820
SHBG	P04278-1	19522.17	2353.77	25623.44	3143.70	0.517 (0.255 - 1.048)	0.067	0.944
IGF1	P05019; P05019-2; P05019-3; P05019-4	9773.75	1619.41	11186.51	1577.12	0.896 (0.514 - 1.563)	0.700	0.029
ALDOB	P05062	2833.98	788.15	3784.16	886.50	1.28 (0.591 - 2.772)	0.531	0.362
APOD	P05090	308003.75	37953.64	333987.19	35135.83	0.975 (0.492 - 1.932)	0.942	0.610
SERPINA5	P05154	5747.31	930.82	10999.47	2148.39	0.796 (0.379 - 1.673)	0.548	0.572
SERPING1	P05155; P05155-2; P05155-3	488314.12	17037.36	521323.59	20786.63	0.717 (0.412 - 1.249)	0.240	0.927
CFI	P05156	81317.78	6779.41	76512.93	5287.76	0.716 (0.335 - 1.533)	0.390	0.536
F13B	P05160	34031.62	3277.67	34220.57	3560.77	0.733 (0.347 - 1.55)	0.417	0.692
CLEC3B	P05452	44547.67	5064.99	45142.11	4895.01	1.161 (0.546 - 2.469)	0.698	0.974
SERPINA7	P05543	4050.57	591.12	4281.29	664.78	1.215 (0.591 - 2.499)	0.597	0.548
SERPIND1	P05546	62058.84	5959.17	65616.58	3953.55	0.591 (0.268 - 1.304)	0.193	0.402
IGKV4-1	P06312	178860.41	17716.95	149752.64	14861.67	1.11 (0.617 - 1.996)	0.728	0.006
GSN	P06396	76561.03	3899.37	84518.02	6118.04	0.878 (0.396 - 1.946)	0.749	0.003
ATP5F1B	P06576	2726.84	523.54	3428.25	676.78	1.061 (0.528 - 2.131)	0.869	0.161
C2	P06681-1	47419.66	7487.33	40002.72	4292.16	1.032 (0.51 - 2.089)	0.931	0.567

Gene name	UniProt ID	ME/CFS		Controls		Linear		Quadratic effect p-value[1]
		Mean	SEM	Mean	SEM	OR (95% CI)	p-value	
APOA4	P06727	425522.17	29080.01	488615.89	24795.04	0.465 (0.224 - 0.964)	0.040	0.296
PROS1	P07225	42319.16	2137.89	42209.89	2243.47	1.326 (0.693 - 2.535)	0.394	0.720
C8A	P07357	40335.63	2926.54	38128.49	2515.43	1.348 (0.642 - 2.831)	0.430	0.465
C8B	P07358	115512.91	20703.56	105434.62	8970.22	0.707 (0.398 - 1.258)	0.239	0.142
C8G	P07360	55515.29	4379.45	53361.31	3584.93	1.401 (0.67 - 2.932)	0.370	0.075
PFN1	P07737	36535.66	3484.50	34765.45	2962.90	1.01 (0.507 - 2.011)	0.978	0.067
THBS1	P07996; P07996-2	57362.07	6144.39	53627.85	5622.61	1.142 (0.557 - 2.342)	0.717	0.185
SERPINA6	P08185	8879.04	1149.96	10362.30	1121.77	1.051 (0.397 - 2.782)	0.921	0.060
LPA	P08519	92065.94	22133.64	92390.47	17957.28	1.003 (0.444 - 2.266)	0.994	0.410
PLEK	P08567	17517.24	3420.37	20733.75	3703.03	1.021 (0.532 - 1.958)	0.951	0.530
CD14	P08571	17965.97	2478.96	21342.89	2601.56	0.965 (0.502 - 1.857)	0.915	0.256
CFH	P08603-1	711805.08	86302.14	611440.21	51919.60	0.888 (0.509 - 1.546)	0.674	0.116
FCGR3A	P08637	15224.75	2767.71	11812.52	2093.83	2.258 (0.873 - 5.838)	0.093	0.150
SERPINF2	P08697-1	58592.00	2837.25	58539.49	1810.28	1.366 (0.821 - 2.273)	0.230	0.226
C1S	P09871	182444.23	22375.75	152581.29	12825.40	0.919 (0.515 - 1.64)	0.774	0.101
C4A	P0C0L4-1	981681.44	140400.28	804827.26	89222.52	0.907 (0.61 - 1.35)	0.632	0.043
C4B	P0C0L5	67004.05	7549.35	58664.60	5395.26	1.881 (0.862 - 4.105)	0.113	0.695
SAA1	P0DJI8	59180.53	27520.00	23085.75	3763.25	1.843 (0.644 - 5.272)	0.254	0.808
IGHA2	P0DOX2	2770944.14	318953.45	3281232.25	301370.78	1.294 (0.606 - 2.763)	0.506	0.596
IGD	P0DOX3	26752.70	10078.76	15037.15	3812.50	1.256 (0.595 - 2.651)	0.549	0.019
IGG1	P0DOX5	4680402.85	315572.50	5035154.28	336914.81	0.856 (0.396 - 1.849)	0.692	0.286
IGM	P0DOX6	37609.02	5581.32	32070.84	5517.53	3.267 (0.931 - 11.469)	0.065	0.115
IGL	P0DOX7	3356181.12	343582.04	2881453.89	366388.42	2.116 (0.808 - 5.545)	0.127	0.093
IGK	P0DOX8	3574661.55	382659.30	3215365.37	309723.94	0.915 (0.567 - 1.477)	0.715	**0.000**
IGLC3	P0DOY3	1076230.46	114731.83	1055223.88	100171.90	1.174 (0.577 - 2.39)	0.658	0.596
C7	P10643	49092.78	4153.85	47687.09	4766.22	0.801 (0.434 - 1.476)	0.477	0.582
CLU	P10909-1; P10909-2; P10909-4; P10909-5	254148.33	14508.11	260549.83	12293.21	0.68 (0.369 - 1.254)	0.217	0.394
HSPA8; HSPA2	P11142-1; P11142-2; P54652	5789.35	502.19	6448.58	658.39	0.789 (0.404 - 1.539)	0.487	0.376
MBL2	P11226	6429.17	876.61	5540.63	697.99	0.811 (0.347 - 1.898)	0.629	0.251
CETP	P11597-1; P11597-2	4711.22	2773.22	3082.96	444.82	0.361 (0.143 - 0.908)	0.030	0.285
F5	P12259	9052.27	443.98	8448.59	453.75	2.617 (0.977 - 7.009)	0.056	0.481
KRT10	P13645	5996.88	2678.27	7764.23	2609.62	0.777 (0.383 - 1.575)	0.484	0.169
C6	P13671	153431.38	11012.02	164335.68	11720.20	0.674 (0.384 - 1.181)	0.168	0.999
SELL	P14151; P14151-2	46994.45	9647.30	39931.32	6807.73	1.041 (0.517 - 2.099)	0.910	0.783
PKM	P14618	28269.70	14128.32	103975.41	57192.48	0.886 (0.432 - 1.815)	0.741	0.803
PVR	P15151-1; P15151-2; P15151-3; P15151-4	1726.73	295.21	2474.14	324.22	0.363 (0.15 - 0.882)	0.025	0.002
RAC2; RAC3; RAC1	P15153; P60763; P63000-1; P63000-2	616.97	48.07	834.35	179.20	1.17 (0.725 - 1.89)	0.520	0.366
CPN1	P15169	15710.18	1636.99	17641.08	2653.05	1.093 (0.506 - 2.361)	0.821	0.351
IGLL1	P15814	17150.04	3256.97	14236.56	2666.94	1.132 (0.528 - 2.425)	0.751	0.859
CD44	P16070; P16070-10; P16070-11; P16070-12; P16070-13; P16070-14; P16070-15; P16070-16; P16070-17; P16070-18; P16070-3; P16070-4; P16070-5; P16070-6; P16070-7; P16070-8; P16070-9	21243.05	1952.30	22506.21	2374.63	1.026 (0.493 - 2.137)	0.945	0.630
HSPA6;HSPA7	P17066; P48741	2966.61	540.89	3951.82	1026.97	1.188 (0.53 - 2.661)	0.675	0.959
IGFBP3	P17936; P17936-2	36875.08	4239.12	38426.17	3696.27	0.574 (0.265 - 1.244)	0.160	0.370
LBP	P18428	4395.47	915.74	2442.18	539.91	1.803 (0.902 - 3.6)	0.095	0.483
ORM2	P19652	34580.86	3901.51	40348.87	7505.27	1.353 (0.57 - 3.213)	0.493	0.901
ITIH2	P19823	691895.75	35441.07	783473.34	39928.63	0.685 (0.362 - 1.297)	0.246	0.652
ITIH1	P19827-1	407271.65	25309.75	380960.93	25205.43	1.262 (0.629 - 2.531)	0.512	0.047

Gene name	UniProt ID	ME/CFS		Controls		Linear		Quadratic effect p-value[1]
		Mean	SEM	Mean	SEM	OR (95% CI)	p-value	
PZP	P20742	195550.09	19532.97	186580.36	16975.72	0.69 (0.391 - 1.218)	0.201	0.008
C4BPB	P20851; P20851-2	35273.00	3121.22	27517.09	2079.57	1.367 (0.552 - 3.383)	0.499	0.989
FLNA	P21333; P21333-2	6288.52	799.06	5441.21	556.77	2.104 (0.82 - 5.398)	0.122	0.893
GPX3	P22352	35384.86	5098.64	37914.24	5084.52	0.654 (0.291 - 1.471)	0.305	0.517
CPN2	P22792	43565.91	1803.53	43195.77	2103.98	0.726 (0.325 - 1.625)	0.436	0.589
PROZ	P22891-1; P22891-2	9294.10	1133.54	10713.39	1072.83	0.902 (0.423 - 1.924)	0.790	0.184
IGHV1-2	P23083	5224.14	1321.42	4216.45	991.28	1.224 (0.568 - 2.639)	0.606	0.571
FBLN1	P23142	39125.01	5533.02	42141.10	5454.15	0.779 (0.378 - 1.604)	0.498	0.751
FBLN1	P23142-4	56241.96	7751.07	59540.26	8795.24	0.79 (0.408 - 1.531)	0.485	0.470
CFL1	P23528	3894.74	1177.00	5407.37	1216.07	0.952 (0.48 - 1.889)	0.888	0.111
IGFBP5	P24593	2571.91	602.18	2439.85	737.32	1.804 (0.852 - 3.818)	0.123	0.842
AZGP1	P25311	396953.28	70938.47	258476.78	61853.02	1.421 (0.611 - 3.307)	0.415	0.089
MST1	P26927	9170.37	1703.02	8349.46	614.77	0.984 (0.582 - 1.663)	0.952	0.014
PON1	P27169	136941.24	9883.23	134502.22	8709.16	0.796 (0.494 - 1.281)	0.347	0.346
CFP	P27918	62871.60	17671.85	61138.10	17866.88	0.798 (0.451 - 1.411)	0.437	0.512
SERPINA4	P29622	23836.26	11086.22	15955.29	5693.56	0.916 (0.495 - 1.697)	0.781	0.484
PRDX6	P30041	1919.24	297.20	1960.01	398.67	1.654 (0.702 - 3.898)	0.250	0.427
PDIA3	P30101	9987.46	1871.55	10124.70	2162.31	1.582 (0.736 - 3.403)	0.240	0.743
KRT9	P35527	5787.53	1483.67	12879.74	7049.52	0.917 (0.427 - 1.97)	0.824	0.523
SAA4	P35542	72333.19	6746.32	67000.85	8994.27	0.639 (0.298 - 1.371)	0.250	0.209
IGFALS	P35858; P35858-2	63038.27	3983.94	66859.66	2916.79	0.495 (0.247 - 0.99)	0.047	0.078
KRT2	P35908	2322.77	590.60	1922.33	375.63	1.133 (0.571 - 2.249)	0.722	0.151
SERPINF1	P36955	37144.37	2126.85	35006.05	2280.18	0.996 (0.588 - 1.689)	0.989	0.058
CFHR2	P36980-1	54582.65	6161.30	43232.07	4199.59	0.96 (0.46 - 2.004)	0.913	0.471
PTGDS	P41222	3702.22	1132.66	4353.99	1733.18	1.185 (0.639 - 2.197)	0.591	0.267
BTD	P43251; P43251-2; P43251-3; P43251-4	25945.13	6486.07	28556.82	4665.68	0.7 (0.327 - 1.498)	0.358	0.598
AFM	P43652	137797.07	8581.00	133754.54	6624.91	0.753 (0.456 - 1.245)	0.269	0.183
MASP1	P48740-1	7398.88	1221.09	7599.06	923.52	0.978 (0.468 - 2.044)	0.953	0.971
MASP1	P48740-2; P48740-4	2320.77	638.21	1869.68	393.20	0.918 (0.385 - 2.189)	0.846	0.981
SELENOP	P49908	33054.76	2604.32	32546.04	2433.04	0.793 (0.432 - 1.456)	0.455	0.265
CAMP	P49913	10187.15	2209.04	6188.42	1621.66	2.073 (0.862 - 4.987)	0.103	0.426
LUM	P51884	72821.16	6810.32	74131.39	5980.93	0.505 (0.238 - 1.072)	0.075	0.072
CRISP3	P54108-1; P54108-2; P54108-3	2273.49	520.26	1457.36	360.94	1.487 (0.691 - 3.198)	0.310	0.300
APOC4	P55056	4087.91	614.08	3228.52	424.85	0.793 (0.312 - 2.016)	0.626	0.130
PLTP	P55058	4912.61	1602.62	4549.31	1369.67	0.774 (0.379 - 1.582)	0.483	0.133
CDH13	P55290; P55290-4	2662.11	520.62	3156.51	716.66	1.244 (0.668 - 2.317)	0.490	0.483
DEFA1; DEFA1; DEFA1B	P59665; P59666	25618.34	4287.37	23239.29	3788.34	0.881 (0.448 - 1.729)	0.712	0.551
ACTB; ACTG1	P60709; P63261	493862.98	43987.72	517271.04	39488.58	0.791 (0.432 - 1.45)	0.449	0.507
RAP1A; RAP1B	P61224-1; P61224-2; P61224-3; P61224-4; P62834	5746.81	731.56	6878.80	789.36	0.849 (0.368 - 1.957)	0.701	0.448
B2M	P61769	9574.86	1337.25	7031.17	1030.48	0.847 (0.393 - 1.822)	0.670	0.568
PPIA	P62937	4990.75	1106.00	3394.40	857.45	2.55 (0.996 - 6.529)	0.051	0.422
YWHAZ	P63104-1	4174.88	432.42	4363.31	356.00	1.996 (0.73 - 5.461)	0.178	0.449
TUBA1B; TUBA1A; TUBA1C	P68363; P68363-2; Q71U36; Q71U36-2; Q9BQE3	25251.04	3980.34	34568.38	6494.80	0.688 (0.259 - 1.823)	0.452	0.067
HBB	P68871	139580.88	13992.87	122471.01	10149.04	1.062 (0.564 - 1.997)	0.853	0.111
HBA1	P69905	56676.89	11352.97	46182.97	5239.84	2.478 (0.846 - 7.258)	0.098	0.219
GPLD1	P80108	17200.34	1889.70	12531.95	899.78	1.127 (0.553 - 2.296)	0.741	0.127
IGLV3-21	P80748	67238.73	7753.71	71013.87	7711.09	0.829 (0.334 - 2.06)	0.686	0.887
CFHR3	Q02985-1; Q02985-2	1567.14	1301.39	11899.08	11426.11	0.948 (0.509 - 1.765)	0.867	0.323
CFHR1	Q03591	45452.93	4354.19	43712.39	4640.73	0.874 (0.43 - 1.777)	0.710	0.376

Gene name	UniProt ID	ME/CFS		Controls		Linear		Quadratic effect p-value[1]
		Mean	SEM	Mean	SEM	OR (95% CI)	p-value	
HGFAC	Q04756	20981.78	2889.45	19723.47	1924.56	0.877 (0.468 - 1.643)	0.682	0.377
ITIH3	Q06033-1; Q06033-2	5944.95	898.09	6482.16	732.17	1.192 (0.519 - 2.738)	0.678	0.246
PRDX1	Q06830	3226.82	830.90	4074.98	1039.42	1.372 (0.628 - 2.996)	0.427	0.965
POLE	Q07864	69922.72	8917.82	71161.06	13448.93	1.141 (0.573 - 2.272)	0.708	0.804
LGALS3BP	Q08380	9255.76	1186.92	7155.88	798.43	0.695 (0.359 - 1.346)	0.281	0.449
EFEMP1	Q12805; Q12805-2; Q12805-3; Q12805-4; Q12805-5	23983.29	2177.29	25586.78	2521.49	1.221 (0.575 - 2.59)	0.604	0.520
CTTN	Q14247-1; Q14247-2; Q14247-3	871.77	135.51	711.92	81.53	1.708 (0.663 - 4.397)	0.267	0.504
HABP2	Q14520-1; Q14520-2	122499.27	14301.41	96143.20	10021.37	1.243 (0.636 - 2.43)	0.524	0.136
ITIH4	Q14624-1	284208.53	27052.07	272866.69	10249.46	0.574 (0.32 - 1.03)	0.063	0.048
ITIH4	Q14624-2; Q14624-3	79295.40	22035.55	100109.87	17466.76	0.977 (0.451 - 2.119)	0.954	0.121
PCOLCE	Q15113	4082.61	928.46	5381.74	966.93	0.905 (0.393 - 2.082)	0.814	0.538
PON3	Q15166	117591.32	12318.07	91466.72	8492.44	1.963 (0.926 - 4.158)	0.078	0.763
TGFBI	Q15582	3189.00	475.92	3877.63	367.64	0.943 (0.556 - 1.602)	0.829	0.105
ECM1	Q16610; Q16610-4	56294.87	4131.51	56717.51	4159.62	0.723 (0.403 - 1.295)	0.276	0.564
HYI	Q5T013; Q5T013-2; Q5T013-3; Q5T013-4	326630.08	63723.79	365729.02	45039.51	0.607 (0.335 - 1.101)	0.100	0.244
PLXDC2	Q6UX71-1; Q6UX71-2	3885.36	739.12	3622.47	482.01	0.839 (0.422 - 1.668)	0.617	0.049
PI16	Q6UXB8-1; Q6UXB8-2	13013.96	2361.07	8781.12	1589.89	1.025 (0.525 - 2.001)	0.942	0.001
FERMT3	Q86UX7; Q86UX7-2	10369.24	1551.33	8192.23	1369.34	1.53 (0.692 - 3.385)	0.294	0.259
TMPRSS6	Q8IU80-1; Q8IU80-4; Q8IU80-5	3285.78	619.66	2172.12	409.87	1.191 (0.67 - 2.116)	0.551	0.008
PATJ	Q8NI35; Q8NI35-2; Q8NI35-3; Q8NI35-4; Q8NI35-5	15512.39	1433.21	16380.55	1162.39	0.928 (0.521 - 1.655)	0.801	0.259
SUN3	Q8TAQ9-1; Q8TAQ9-2; Q8TAQ9-3	42493.82	5047.21	38888.46	3376.37	0.505 (0.249 - 1.025)	0.058	0.009
CFHR4	Q92496; Q92496-2	26325.50	3886.60	19045.18	2401.31	1.614 (0.688 - 3.789)	0.271	0.122
PRG4	Q92954-1; Q92954-3; Q92954-6	12812.86	1601.51	11258.67	1295.00	1.049 (0.501 - 2.197)	0.900	0.527
CPB2	Q96IY4	19443.92	1409.15	18074.83	858.28	0.749 (0.375 - 1.496)	0.412	0.011
CNDP1	Q96KN2	14085.73	3111.56	9098.63	3101.30	1.588 (0.776 - 3.253)	0.206	0.900
FCRL3	Q96P31-1; Q96P31-2; Q96P31-3; Q96P31-4; Q96P31-5; Q96P31-6; Q96P31-7	6961.08	1799.81	12718.52	2474.09	0.394 (0.122 - 1.27)	0.119	0.111
PGLYRP2	Q96PD5; Q96PD5-2	155906.20	13732.39	139750.46	8841.93	0.885 (0.578 - 1.356)	0.576	0.144
MENT	Q9BUN1	2688.82	432.45	2832.51	507.59	1.233 (0.621 - 2.449)	0.550	0.567
COLEC11	Q9BWP8; Q9BWP8-10; Q9BWP8-2; Q9BWP8-3; Q9BWP8-4; Q9BWP8-5; Q9BWP8-6; Q9BWP8-7; Q9BWP8-8; Q9BWP8-9	1835.31	376.58	2565.65	563.13	0.686 (0.301 - 1.564)	0.371	0.816
CFHR5	Q9BXR6	29834.63	11153.35	13526.76	2427.48	1.972 (0.783 - 4.966)	0.149	0.473
SH3BGRL3	Q9H299	21176.76	3469.57	20296.40	2301.76	1.01 (0.471 - 2.166)	0.981	0.503
CRTAC1	Q9NQ79; Q9NQ79-2; Q9NQ79-3	8360.14	559.00	11803.87	983.85	0.789 (0.38 - 1.641)	0.526	0.367
C1RL	Q9NZP8	16656.34	2830.60	12433.38	1259.91	1.078 (0.49 - 2.373)	0.852	0.796
FETUB	Q9UGM5-1	12964.44	1911.25	13359.59	2106.63	0.855 (0.364 - 2.007)	0.719	0.834
SERPINA10	Q9UK55	8019.87	948.67	8203.11	915.19	0.79 (0.389 - 1.605)	0.515	0.124
TLN1	Q9Y490	50207.48	6411.95	43628.71	4597.76	0.762 (0.432 - 1.344)	0.348	0.415
FARP1	Q9Y4F1; Q9Y4F1-2	296.39	9.56	5084.90	2690.75	0.968 (0.452 - 2.074)	0.933	0.032
PCDHGC5	Q9Y5F6-2	3065.99	1057.09	2310.96	500.70	1.279 (0.615 - 2.66)	0.510	0.199
FCGBP	Q9Y6R7	8723.35	1686.72	5510.87	1432.51	1.284 (0.66 - 2.498)	0.461	0.184
