# Supplementary material for: Plasma proteomic profiling suggests an association between antigen driven clonal B cell expansion and ME/CFS
Source: PLoS One. 2020 Jul 21;15(7):e0236148. doi: 10.1371/journal.pone.0236148 (PMC7373296; doi:10.1371/journal.pone.0236148)
Supplement: S4 Table — aOR, 95% confidence levels and p-values from the logistic regression model in which only the linear term of the protein levels was fitted as an independent variable are shown. Quadratic effect p-value corresponds to the likelihood ratio tests that compare the goodness-of-fit of the model with both linear and quadratic terms of the protein levels to that of the model with only the linear term. ME/CFS: myalgic encephalomyelitis/chronic fatigue syndrome, sr-IBS: self-reported irritable bowel syndrome, aOR: adjusted odds ratio, CI: confidence interval. 1Quadratic effect p-value: crude p-value of the likelihood ratio test comparing the goodness-of-fit between the logistic regression model with both linear and quadratic terms of the protein level and the model with only the linear term. Hochberg step-up procedure was applied to correct for the multiple tests over the annotated proteins controlling the family-wise error rate (FWER) at the level of 0.05. (PDF) [file pone.0236148.s006.pdf]

Supplementary Table 4. Statistical analysis of individual protein associations with ME/CFS with sr-IBS and ME/CFS without sr-IBS.

Gene name	UniProt ID	ME/CFS with sr-IBS			ME/CFS without sr-IBS		
		Linear		Quadratic effect	Linear		Quadratic effect
		OR (95% CI)	p-value	p-value[1]	OR (95% CI)	p-value	p-value[1]
IGLV4-69	A0A075B6H9	0.613 (0.271 - 1.385)	0.239	0.974	0.732 (0.382 - 1.403)	0.348	0.654
IGLV8-61	A0A075B6I0	0.908 (0.431 - 1.914)	0.800	0.538	1.156 (0.588 - 2.273)	0.673	0.187
IGLV4-60	A0A075B6I1	0.777 (0.326 - 1.849)	0.568	0.298	1.169 (0.665 - 2.053)	0.587	0.262
IGLV2-18	A0A075B6J9	0.941 (0.404 - 2.19)	0.888	0.129	0.892 (0.438 - 1.817)	0.754	0.333
IGLV3-10	A0A075B6K4	0.918 (0.5 - 1.686)	0.784	0.023	0.823 (0.466 - 1.451)	0.501	0.032
IGKV2D-30	A0A075B6S6	0.923 (0.451 - 1.888)	0.826	0.034	1.243 (0.691 - 2.235)	0.467	0.125
IGKV3D-15	A0A087WSY6	0.997 (0.404 - 2.461)	0.994	0.195	0.834 (0.435 - 1.599)	0.586	0.653
IGKV3D-11; IGKV3-11	A0A0A0MRZ8; P04433	1.231 (0.6 - 2.527)	0.570	0.808	1.012 (0.643 - 1.595)	0.956	**0.000**
IGHV3-49	A0A0A0MS15	0.889 (0.521 - 1.514)	0.664	0.168	1.381 (0.783 - 2.437)	0.264	0.055
IGKV6D-21	A0A0A0MT36	0.748 (0.325 - 1.72)	0.494	0.936	0.682 (0.378 - 1.23)	0.204	0.112
IGHV6-1	A0A0B4J1U7	0.533 (0.213 - 1.335)	0.179	0.472	0.689 (0.335 - 1.42)	0.314	0.798
IGHV3-15	A0A0B4J1V0	1.166 (0.516 - 2.635)	0.711	0.817	0.624 (0.321 - 1.214)	0.166	0.229
IGHV2-26	A0A0B4J1V2	1.013 (0.491 - 2.092)	0.971	0.792	1.427 (0.753 - 2.701)	0.275	0.043
IGHV3-74	A0A0B4J1X5	1.272 (0.62 - 2.606)	0.512	1.000	1.885 (1.007 - 3.526)	0.047	0.739
IGHV3-72	A0A0B4J1Y9	0.762 (0.359 - 1.62)	0.480	0.143	1.542 (0.715 - 3.321)	0.269	0.004
IGHV1D-13	A0A0B4J2D9	0.39 (0.122 - 1.252)	0.114	0.835	1.265 (0.627 - 2.549)	0.511	0.976
IGHV1-69D; IGHV1-69	A0A0B4J2H0; P01742	0.99 (0.488 - 2.006)	0.977	0.425	1.602 (0.832 - 3.083)	0.158	0.495
IGKV6-21	A0A0C4DH24	1.45 (0.6 - 3.502)	0.409	0.020	1.875 (0.899 - 3.912)	0.094	0.578
IGKV3D-20	A0A0C4DH25	0.947 (0.442 - 2.029)	0.889	0.921	1.718 (0.875 - 3.372)	0.115	0.000
IGHV1-18	A0A0C4DH31	0.591 (0.266 - 1.313)	0.197	0.559	1.243 (0.567 - 2.723)	0.587	0.210
IGHV5-51	A0A0C4DH38	0.924 (0.392 - 2.174)	0.856	0.341	1.579 (0.77 - 3.235)	0.212	0.169
IGHV4-61; IGHV4-39; IGHV 4-59; IGHV 4-34; IGHV 4-30-4; IGHV 4-38-2	A0A0C4DH41; P01824; P01825; P06331; P0DP06; P0DP08	1.239 (0.716 - 2.144)	0.444	0.092	1.345 (0.769 - 2.353)	0.298	0.026
IGKV1-8	A0A0C4DH67	1.011 (0.501 - 2.044)	0.975	0.974	1.06 (0.536 - 2.095)	0.866	0.836
IGKV2-24	A0A0C4DH68	1.148 (0.538 - 2.452)	0.721	0.300	1.328 (0.652 - 2.705)	0.434	0.887
IGKV1-12; IGKV1D-12; IGKV1D-39	A0A0C4DH73; P01611; P04432	1.159 (0.461 - 2.914)	0.754	0.094	1.044 (0.546 - 1.995)	0.896	0.123
IGHV50-10-1	A0A0J9YXX1	1.684 (0.737 - 3.849)	0.217	0.287	0.92 (0.432 - 1.956)	0.829	0.849
IGLC7	A0M8Q6	1.502 (0.621 - 3.629)	0.366	**0.000**	0.378 (0.171 - 0.836)	0.016	0.502
MASP2	O00187-1; O00187-2	0.691 (0.352 - 1.354)	0.281	0.073	0.695 (0.401 - 1.203)	0.194	0.246
QSOX1	O00391; O00391-2	1.697 (0.692 - 4.16)	0.248	0.239	1.276 (0.624 - 2.611)	0.503	0.261
NRP1	O14786-1; O14786-2; O14786-3	0.4 (0.162 - 0.986)	0.047	0.316	1.125 (0.712 - 1.777)	0.613	0.250
APOL1	O14791; O14791-2; O14791-3	1.759 (0.864 - 3.579)	0.119	0.881	1.608 (0.916 - 2.823)	0.098	0.929
CEP290	O15078-1; O15078-2	0.797 (0.319 - 1.995)	0.629	0.894	0.703 (0.324 - 1.526)	0.374	0.854
CD5L	O43866	1.76 (0.755 - 4.101)	0.190	0.909	1.446 (0.717 - 2.917)	0.302	0.522
FCN3	O75636-1	2.114 (0.891 - 5.014)	0.089	0.874	1.574 (0.78 - 3.174)	0.205	0.173
ATRN	O75882-1; O75882-2; O75882-3	0.769 (0.378 - 1.566)	0.470	0.525	1.617 (0.797 - 3.278)	0.182	0.823
APOM	O95445-1; O95445-2	0.846 (0.433 - 1.654)	0.625	0.150	0.722 (0.405 - 1.285)	0.268	0.039
CP	P00450	0.575 (0.266 - 1.242)	0.159	0.965	0.892 (0.486 - 1.635)	0.712	0.958
F13A1	P00488	0.953 (0.409 - 2.223)	0.912	0.261	0.913 (0.457 - 1.824)	0.798	0.548
F2	P00734	1.11 (0.571 - 2.156)	0.759	0.193	1.324 (0.731 - 2.397)	0.353	0.116
C1R	P00736	1.258 (0.666 - 2.377)	0.479	0.792	1.297 (0.715 - 2.353)	0.390	0.573
HP	P00738	0.694 (0.335 - 1.44)	0.327	0.338	0.686 (0.395 - 1.19)	0.181	0.185
HPR	P00739-1; P00739-2	0.936 (0.531 - 1.648)	0.818	0.003	0.879 (0.507 - 1.524)	0.647	0.359
F9	P00740	1.206 (0.631 - 2.305)	0.570	0.748	0.88 (0.43 - 1.8)	0.728	0.991
F10	P00742	1.162 (0.553 - 2.445)	0.692	0.730	1.196 (0.633 - 2.26)	0.581	0.278
CFD	P00746	1.207 (0.491 - 2.967)	0.682	0.845	0.735 (0.365 - 1.479)	0.389	0.020
PLG	P00747	0.805 (0.395 - 1.642)	0.551	0.751	1.027 (0.565 - 1.867)	0.928	0.705
F12	P00748	1.605 (0.646 - 3.985)	0.308	0.986	1.188 (0.662 - 2.132)	0.562	0.285

Gene name	UniProt ID	ME/CFS with sr-IBS			ME/CFS without sr-IBS		
		Linear		Quadratic effect	Linear		Quadratic effect
CFB	P00751-1	1.094 (0.552 - 2.172)	0.796	0.223	0.769 (0.377 - 1.568)	0.470	0.822
SERPINC1	P01008	1.236 (0.55 - 2.774)	0.608	0.727	1.04 (0.613 - 1.765)	0.883	0.054
SERPINA1	P01009-1	0.204 (0.041 - 1.011)	0.052	0.255	0.596 (0.224 - 1.588)	0.301	0.399
SERPINA3	P01011-1	0.801 (0.443 - 1.445)	0.461	0.005	1.359 (0.835 - 2.21)	0.216	0.302
AGT	P01019	1.953 (0.862 - 4.426)	0.109	0.070	1.21 (0.688 - 2.128)	0.507	0.253
A2M	P01023	0.965 (0.453 - 2.052)	0.925	0.207	0.817 (0.515 - 1.295)	0.391	0.280
C3	P01024	1.188 (0.641 - 2.201)	0.583	0.007	0.802 (0.521 - 1.233)	0.315	0.527
C5	P01031	0.832 (0.411 - 1.687)	0.611	0.482	1.015 (0.621 - 1.658)	0.952	0.051
CST3	P01034	1.119 (0.509 - 2.462)	0.780	0.458	1.162 (0.622 - 2.171)	0.636	0.385
KNG1	P01042	2.564 (0.829 - 7.93)	0.102	0.209	4.115 (1.14 - 14.857)	0.031	0.312
Isoform LMW KNG1	P01042-2	1.044 (0.509 - 2.14)	0.906	0.741	0.441 (0.205 - 0.945)	0.035	0.445
IGF2	P01344; P01344-2; P01344-3	0.647 (0.302 - 1.386)	0.263	0.310	1.012 (0.587 - 1.744)	0.965	0.155
JCHAIN	P01591	1.083 (0.441 - 2.66)	0.862	0.901	1.151 (0.647 - 2.046)	0.631	0.082
IGKV1-33	P01594	1.052 (0.444 - 2.492)	0.908	0.663	1.18 (0.573 - 2.426)	0.652	0.061
IGKV1-17	P01599	1.704 (0.775 - 3.746)	0.185	0.230	1.246 (0.748 - 2.073)	0.397	0.004
IGKV1D-16	P01601	1.49 (0.644 - 3.45)	0.351	0.345	1.34 (0.654 - 2.746)	0.423	0.455
IGKV1-5	P01602	0.802 (0.386 - 1.666)	0.555	0.671	1.348 (0.777 - 2.337)	0.287	0.415
IGKV2D-40	P01614	1.118 (0.504 - 2.478)	0.784	0.201	1.445 (0.66 - 3.161)	0.356	0.340
IGKV3-20	P01619	0.856 (0.372 - 1.97)	0.715	0.480	1.42 (0.762 - 2.645)	0.269	0.024
IGKV3-15	P01624	0.655 (0.278 - 1.546)	0.334	0.314	0.508 (0.271 - 0.95)	0.034	0.281
IGLV1-44	P01699	1.894 (0.859 - 4.179)	0.114	0.619	0.954 (0.454 - 2.004)	0.903	0.921
IGLV1-47	P01700	0.632 (0.318 - 1.256)	0.190	0.996	0.613 (0.35 - 1.073)	0.087	0.448
IGLV1-51	P01701	1.224 (0.524 - 2.86)	0.640	0.955	0.782 (0.398 - 1.539)	0.478	0.667
IGLV2-23	P01705	0.615 (0.267 - 1.419)	0.255	0.758	1.147 (0.576 - 2.283)	0.696	0.114
IGLV2-11	P01706	0.539 (0.22 - 1.321)	0.177	0.478	0.969 (0.506 - 1.853)	0.924	0.279
IGLV2-8	P01709	0.923 (0.396 - 2.156)	0.854	0.139	1.292 (0.631 - 2.648)	0.483	0.118
IGLV3-19	P01714	0.913 (0.421 - 1.982)	0.819	0.801	1.065 (0.522 - 2.172)	0.861	0.048
IGLV3-27	P01718	0.787 (0.386 - 1.605)	0.510	0.588	1.126 (0.63 - 2.015)	0.687	0.015
IGHV3-23; IGHV3-30	P01764; P01768	1.739 (0.712 - 4.25)	0.225	0.545	0.844 (0.55 - 1.296)	0.441	**0.000**
IGHV3-13	P01766	1.16 (0.536 - 2.511)	0.707	0.878	1.308 (0.634 - 2.699)	0.467	0.277
IGHV3-7	P01780	0.771 (0.392 - 1.518)	0.452	0.672	0.95 (0.569 - 1.584)	0.845	0.107
IGHV3-9	P01782	1.8 (0.761 - 4.258)	0.181	0.009	1.802 (0.879 - 3.695)	0.108	0.593
PIGR	P01833	2.952 (1.43 - 6.095)	0.003	0.744	2.121 (1.09 - 4.125)	0.027	0.371
IGKC	P01834	0.833 (0.352 - 1.974)	0.679	0.240	0.875 (0.534 - 1.431)	0.595	0.041
IGHG2	P01859	1.437 (0.633 - 3.262)	0.386	0.589	1.273 (0.628 - 2.58)	0.502	0.006
IGHG3	P01860	1.016 (0.45 - 2.294)	0.969	0.003	0.858 (0.414 - 1.778)	0.682	0.268
IGHG4	P01861	0.684 (0.279 - 1.679)	0.408	0.336	0.976 (0.566 - 1.684)	0.933	0.031
IGHM	P01871; P01871-2	0.989 (0.452 - 2.165)	0.978	0.852	1.177 (0.657 - 2.11)	0.582	0.552
IGHA1	P01876	1.119 (0.492 - 2.548)	0.788	0.828	0.867 (0.481 - 1.563)	0.637	0.225
IGHA2	P01877	2.852 (1.017 - 8.003)	0.046	0.314	1.23 (0.679 - 2.229)	0.493	0.291
KRT14; KRT16	P02533; P08779	N/A	N/A	N/A	1.099 (0.676 - 1.786)	0.701	0.444
APOA1	P02647	1.299 (0.625 - 2.698)	0.483	0.053	0.756 (0.471 - 1.213)	0.247	0.082
APOE	P02649	2.428 (0.983 - 5.993)	0.054	0.277	0.677 (0.358 - 1.279)	0.230	0.395
APOA2	P02652	1.115 (0.542 - 2.292)	0.768	0.160	0.766 (0.44 - 1.335)	0.348	0.224
APOC1	P02654	1.795 (0.689 - 4.678)	0.231	0.300	1.625 (0.809 - 3.261)	0.172	0.893
APOC2	P02655	0.853 (0.582 - 1.249)	0.413	0.040	1.006 (0.772 - 1.311)	0.962	0.007
APOC3	P02656	2.782 (1.061 - 7.293)	0.037	0.039	0.879 (0.461 - 1.676)	0.698	0.392
FGA	P02671-1	1.425 (0.521 - 3.893)	0.490	0.283	1.645 (0.689 - 3.926)	0.262	0.437
FGB	P02675	0.759 (0.332 - 1.731)	0.512	0.680	1.086 (0.625 - 1.886)	0.769	0.026
FGG	P02679; P02679-2	1.023 (0.531 - 1.973)	0.945	0.430	0.829 (0.572 - 1.2)	0.321	0.450

Gene name	UniProt ID	ME/CFS with sr-IBS			ME/CFS without sr-IBS		
		Linear		Quadratic effect	Linear		Quadratic effect
APCS	P02743	1.833 (0.603 - 5.569)	0.285	0.624	1.534 (0.806 - 2.921)	0.192	0.452
C1QA	P02745	0.812 (0.415 - 1.588)	0.542	0.680	0.739 (0.377 - 1.449)	0.380	0.027
C1QB	P02746	0.524 (0.243 - 1.13)	0.099	0.070	0.969 (0.495 - 1.896)	0.927	0.627
C1QC	P02747	0.568 (0.202 - 1.593)	0.282	0.092	0.977 (0.509 - 1.876)	0.946	0.673
C9	P02748	0.886 (0.422 - 1.86)	0.750	0.405	1.385 (0.642 - 2.986)	0.405	0.457
APOH	P02749	1.277 (0.632 - 2.582)	0.495	0.203	1.324 (0.671 - 2.612)	0.417	0.574
LRG1	P02750	0.524 (0.22 - 1.249)	0.145	0.968	0.705 (0.377 - 1.318)	0.274	0.383
FN1	P02751; P02751-11; P02751-14; P02751-15; P02751-3; P02751-7; P02751-8	1.084 (0.511 - 2.3)	0.834	0.879	0.96 (0.482 - 1.912)	0.909	0.842
RBP4	P02753	1.032 (0.505 - 2.109)	0.932	0.950	0.57 (0.3 - 1.084)	0.087	0.371
AMBP	P02760	1.03 (0.51 - 2.08)	0.935	0.722	0.83 (0.419 - 1.641)	0.593	0.138
ORM1	P02763	0.809 (0.351 - 1.864)	0.618	0.218	1.095 (0.472 - 2.538)	0.831	0.067
AHSG	P02765	1.168 (0.61 - 2.238)	0.640	0.489	1.172 (0.788 - 1.742)	0.433	0.035
TTR	P02766	0.461 (0.185 - 1.147)	0.096	0.868	0.782 (0.446 - 1.369)	0.390	0.035
ALB	P02768-1	0.712 (0.307 - 1.652)	0.429	0.097	0.89 (0.674 - 1.174)	0.412	0.072
GC	P02774; P02774-3	0.754 (0.377 - 1.507)	0.424	0.484	0.656 (0.382 - 1.128)	0.128	0.986
PPBP	P02775	0.871 (0.45 - 1.684)	0.681	0.001	1.15 (0.715 - 1.85)	0.562	0.084
PF4	P02776	1.003 (0.479 - 2.1)	0.994	0.023	1.01 (0.735 - 1.389)	0.947	0.019
TF	P02787	1.132 (0.593 - 2.16)	0.707	0.213	0.712 (0.399 - 1.27)	0.251	0.208
HPX	P02790	0.691 (0.29 - 1.648)	0.405	0.191	0.485 (0.23 - 1.023)	0.057	0.739
ANG	P03950	0.245 (0.071 - 0.85)	0.027	0.303	2.145 (1.043 - 4.411)	0.038	0.044
F11	P03951; P03951-2	1.206 (0.692 - 2.101)	0.508	0.009	1.335 (0.814 - 2.188)	0.251	0.154
KLKB1	P03952	0.993 (0.504 - 1.956)	0.984	0.217	1.405 (0.84 - 2.352)	0.195	0.139
C4BPA	P04003	0.895 (0.406 - 1.973)	0.783	0.463	0.854 (0.588 - 1.241)	0.409	0.244
VTN	P04004	1.597 (0.843 - 3.023)	0.151	0.961	0.885 (0.632 - 1.239)	0.479	0.144
PROC	P04070; P04070-2	0.694 (0.294 - 1.64)	0.405	0.655	1.194 (0.678 - 2.105)	0.538	0.152
ALDOA	P04075; P04075-2	1.2 (0.567 - 2.541)	0.633	0.673	0.803 (0.428 - 1.505)	0.494	0.748
APOB	P04114	1.55 (0.713 - 3.371)	0.269	0.103	2.23 (1.029 - 4.833)	0.042	0.326
LCAT	P04180	0.741 (0.385 - 1.426)	0.369	0.134	1.187 (0.625 - 2.252)	0.599	0.391
HRG	P04196	1.293 (0.656 - 2.548)	0.458	0.276	1.168 (0.62 - 2.202)	0.629	0.159
IGLV7-43	P04211	0.849 (0.438 - 1.646)	0.628	0.058	1.137 (0.588 - 2.198)	0.703	0.863
A1BG	P04217	0.893 (0.44 - 1.812)	0.753	0.181	0.689 (0.393 - 1.208)	0.194	0.609
KRT1	P04264	0.769 (0.355 - 1.666)	0.506	0.171	1.209 (0.629 - 2.324)	0.569	0.168
VWF	P04275	0.617 (0.234 - 1.628)	0.330	0.570	0.622 (0.298 - 1.299)	0.207	0.983
SHBG	P04278-1	0.838 (0.383 - 1.834)	0.659	0.557	0.626 (0.351 - 1.116)	0.113	0.305
IGF1	P05019; P05019-2; P05019-3; P05019-4	0.714 (0.388 - 1.315)	0.279	0.023	1.012 (0.627 - 1.633)	0.960	0.043
ALDOB	P05062	0.907 (0.41 - 2.008)	0.810	0.478	1.555 (0.744 - 3.252)	0.240	0.198
APOD	P05090	0.731 (0.346 - 1.543)	0.411	0.305	0.847 (0.442 - 1.621)	0.617	0.530
SERPINA5	P05154	0.528 (0.228 - 1.22)	0.135	0.334	1.085 (0.556 - 2.117)	0.809	0.597
SERPING1	P05155; P05155-2; P05155-3	0.771 (0.377 - 1.577)	0.477	0.155	0.825 (0.551 - 1.234)	0.350	0.453
CFI	P05156	1.448 (0.716 - 2.927)	0.303	0.570	0.705 (0.347 - 1.432)	0.335	0.241
F13B	P05160	0.853 (0.406 - 1.791)	0.674	0.819	0.681 (0.359 - 1.292)	0.240	0.891
CLEC3B	P05452	0.723 (0.343 - 1.523)	0.393	0.637	1.206 (0.611 - 2.383)	0.588	0.560
SERPINA7	P05543	0.606 (0.266 - 1.384)	0.235	0.260	1.181 (0.617 - 2.258)	0.615	0.489
SERPIND1	P05546	0.691 (0.381 - 1.255)	0.225	0.584	0.683 (0.343 - 1.356)	0.276	0.088
IGKV4-1	P06312	1.346 (0.593 - 3.053)	0.478	0.814	1.094 (0.63 - 1.902)	0.748	0.012
GSN	P06396	0.248 (0.086 - 0.718)	0.010	0.098	0.694 (0.307 - 1.567)	0.380	0.002
ATP5F1B	P06576	0.886 (0.393 - 1.996)	0.770	0.866	1.124 (0.597 - 2.113)	0.717	0.217
C2	P06681-1	1.198 (0.645 - 2.226)	0.567	0.467	1.093 (0.579 - 2.063)	0.784	0.605
APOA4	P06727	0.594 (0.282 - 1.251)	0.171	0.541	0.51 (0.271 - 0.96)	0.037	0.365

Gene name	UniProt ID	ME/CFS with sr-IBS			ME/CFS without sr-IBS		
		Linear		Quadratic effect	Linear		Quadratic effect
PROS1	P07225	0.626 (0.275 - 1.422)	0.263	0.022	1.402 (0.755 - 2.602)	0.284	0.850
C8A	P07357	0.895 (0.427 - 1.875)	0.768	0.871	1.213 (0.634 - 2.32)	0.558	0.381
C8B	P07358	0.92 (0.516 - 1.642)	0.778	0.023	0.85 (0.486 - 1.485)	0.569	0.056
C8G	P07360	1.157 (0.558 - 2.397)	0.695	0.655	1.421 (0.796 - 2.536)	0.234	0.660
PFN1	P07737	0.454 (0.208 - 0.992)	0.048	0.202	0.726 (0.419 - 1.258)	0.254	0.044
THBS1	P07996; P07996-2	0.857 (0.446 - 1.647)	0.644	0.009	0.628 (0.337 - 1.167)	0.142	0.044
SERPINA6	P08185	0.533 (0.217 - 1.311)	0.171	0.435	0.974 (0.453 - 2.093)	0.948	0.639
LPA	P08519	0.898 (0.367 - 2.198)	0.814	0.002	1.072 (0.491 - 2.34)	0.860	0.474
PLEK	P08567	0.799 (0.362 - 1.763)	0.579	0.720	0.793 (0.438 - 1.438)	0.447	0.193
CD14	P08571	0.592 (0.282 - 1.244)	0.166	0.968	0.824 (0.443 - 1.532)	0.541	0.569
CFH	P08603-1	1.198 (0.615 - 2.332)	0.595	0.212	0.79 (0.457 - 1.365)	0.399	0.177
FCGR3A	P08637	1.199 (0.565 - 2.546)	0.636	0.732	1.795 (0.771 - 4.182)	0.175	0.442
SERPINF2	P08697-1	0.882 (0.489 - 1.59)	0.676	0.055	1.678 (0.89 - 3.164)	0.110	0.194
C1S	P09871	1.196 (0.637 - 2.246)	0.577	0.017	0.933 (0.532 - 1.636)	0.811	0.057
C4A	P0C0L4-1	1.459 (0.718 - 2.962)	0.296	0.800	0.947 (0.652 - 1.375)	0.776	0.015
C4B	P0C0L5	1.747 (0.648 - 4.713)	0.270	0.382	1.37 (0.778 - 2.412)	0.275	0.549
SAA1	P0DJI8	1.892 (0.619 - 5.782)	0.263	0.437	0.721 (0.364 - 1.43)	0.350	0.670
IGHA2	P0DOX2	0.617 (0.288 - 1.318)	0.212	0.216	1.178 (0.595 - 2.332)	0.638	0.646
IGD	P0DOX3	1.222 (0.595 - 2.512)	0.585	0.418	1.217 (0.637 - 2.326)	0.551	0.003
IGG1	P0DOX5	0.361 (0.138 - 0.943)	0.037	0.887	0.701 (0.361 - 1.361)	0.294	0.110
IGM	P0DOX6	0.975 (0.439 - 2.166)	0.950	0.013	1.576 (0.786 - 3.159)	0.199	0.038
IGL	P0DOX7	1.139 (0.458 - 2.835)	0.779	0.651	1.199 (0.546 - 2.629)	0.650	0.095
IGK	P0DOX8	0.916 (0.37 - 2.268)	0.849	0.486	0.754 (0.451 - 1.26)	0.281	0.017
IGLC3	P0DOY3	1.019 (0.464 - 2.238)	0.963	0.731	1.079 (0.548 - 2.126)	0.824	0.834
C7	P10643	1.668 (0.611 - 4.557)	0.318	0.015	0.842 (0.474 - 1.495)	0.558	0.402
CLU	P10909-1; P10909-2; P10909-4; P10909-5	0.771 (0.427 - 1.393)	0.389	0.181	0.675 (0.379 - 1.202)	0.183	0.575
HSPA8; HSPA2	P11142-1; P11142-2; P54652	0.723 (0.312 - 1.676)	0.449	0.723	0.615 (0.324 - 1.167)	0.137	0.548
MBL2	P11226	1.152 (0.509 - 2.605)	0.734	0.467	0.929 (0.447 - 1.928)	0.844	0.684
CETP	P11597-1; P11597-2	0.719 (0.353 - 1.467)	0.365	0.063	0.381 (0.179 - 0.811)	0.012	0.458
F5	P12259	1.812 (0.833 - 3.944)	0.134	0.298	2.443 (1.07 - 5.573)	0.034	0.579
KRT10	P13645	0.989 (0.434 - 2.254)	0.978	0.764	0.746 (0.367 - 1.515)	0.418	0.273
C6	P13671	0.828 (0.408 - 1.683)	0.603	0.088	0.603 (0.33 - 1.103)	0.101	0.781
SELL	P14151; P14151-2	0.922 (0.469 - 1.812)	0.813	0.038	1.007 (0.525 - 1.931)	0.981	0.202
PKM	P14618	0.333 (0.097 - 1.145)	0.081	0.620	0.743 (0.375 - 1.472)	0.395	0.221
PVR	P15151-1; P15151-2; P15151-3; P15151-4	0.861 (0.433 - 1.713)	0.670	0.694	0.571 (0.304 - 1.072)	0.082	0.028
RAC2; RAC3; RAC1	P15153; P60763; P63000-1; P63000-2	0.544 (0.174 - 1.701)	0.296	0.454	1.241 (0.771 - 1.996)	0.373	0.764
CPN1	P15169	0.963 (0.436 - 2.127)	0.925	0.528	1.233 (0.59 - 2.577)	0.577	0.102
IGLL1	P15814	1.128 (0.51 - 2.495)	0.766	0.449	0.768 (0.396 - 1.489)	0.435	0.504
CD44	P16070; P16070-10; P16070-11; P16070-12; P16070-13; P16070-14; P16070-15; P16070-16; P16070-17; P16070-18; P16070-3; P16070-4; P16070-5; P16070-6; P16070-7; P16070-8; P16070-9	0.98 (0.452 - 2.125)	0.958	0.735	1.02 (0.508 - 2.05)	0.954	0.544
HSPA6; HSPA7	P17066; P48741	0.858 (0.34 - 2.163)	0.745	0.950	1.2 (0.551 - 2.612)	0.646	0.995
IGFBP3	P17936; P17936-2	0.814 (0.433 - 1.531)	0.523	0.019	0.601 (0.292 - 1.236)	0.167	0.155
LBP	P18428	1.667 (0.821 - 3.384)	0.158	0.486	1.841 (0.97 - 3.492)	0.062	0.277
ORM2	P19652	1.496 (0.572 - 3.916)	0.412	0.788	0.825 (0.374 - 1.815)	0.633	0.696
ITIH2	P19823	0.604 (0.288 - 1.268)	0.183	0.264	0.578 (0.296 - 1.127)	0.108	0.896
ITIH1	P19827-1	1.443 (0.583 - 3.569)	0.428	0.372	1.436 (0.757 - 2.726)	0.268	0.068
PZP	P20742	2.061 (0.818 - 5.195)	0.125	0.148	0.804 (0.497 - 1.302)	0.377	0.002

Gene name	UniProt ID	ME/CFS with sr-IBS			ME/CFS without sr-IBS		
		Linear		Quadratic effect	Linear		Quadratic effect
C4BPB	P20851; P20851-2	2.953 (1.011 - 8.623)	0.048	0.166	1.151 (0.558 - 2.373)	0.702	0.770
FLNA	P21333; P21333-2	1.045 (0.477 - 2.292)	0.912	0.252	1.161 (0.604 - 2.231)	0.653	0.593
GPX3	P22352	0.84 (0.392 - 1.801)	0.654	0.513	0.664 (0.342 - 1.289)	0.227	0.714
CPN2	P22792	1.174 (0.538 - 2.562)	0.687	0.299	1.052 (0.587 - 1.885)	0.864	0.754
PROZ	P22891-1; P22891-2	1.171 (0.589 - 2.33)	0.653	0.393	1.112 (0.591 - 2.094)	0.741	0.831
IGHV1-2	P23083	1.946 (0.856 - 4.428)	0.112	0.556	1.116 (0.519 - 2.399)	0.778	0.583
FBLN1	P23142	0.94 (0.466 - 1.899)	0.864	0.490	0.779 (0.399 - 1.52)	0.465	0.817
FBLN1	P23142-4	0.741 (0.363 - 1.513)	0.410	0.681	0.758 (0.412 - 1.395)	0.374	0.403
CFL1	P23528	0.607 (0.268 - 1.373)	0.230	0.161	0.914 (0.457 - 1.828)	0.801	0.095
IGFBP5	P24593	1.209 (0.584 - 2.504)	0.610	0.229	1.765 (0.904 - 3.447)	0.096	0.293
AZGP1	P25311	1.97 (0.754 - 5.149)	0.166	0.889	1.084 (0.516 - 2.277)	0.830	0.100
MST1	P26927	1.379 (0.782 - 2.433)	0.267	0.257	1.14 (0.689 - 1.887)	0.609	0.017
PON1	P27169	0.805 (0.403 - 1.609)	0.539	0.132	0.953 (0.637 - 1.426)	0.817	0.053
CFP	P27918	1.186 (0.639 - 2.201)	0.589	0.852	0.843 (0.497 - 1.429)	0.528	0.219
SERPINA4	P29622	1.268 (0.659 - 2.439)	0.477	0.868	0.943 (0.562 - 1.582)	0.825	0.036
PRDX6	P30041	1.102 (0.497 - 2.444)	0.810	0.012	1.949 (0.907 - 4.189)	0.087	0.106
PDIA3	P30101	0.901 (0.459 - 1.766)	0.761	0.305	2.111 (0.879 - 5.071)	0.094	0.741
KRT9	P35527	0.895 (0.407 - 1.968)	0.782	0.640	1.124 (0.57 - 2.218)	0.735	0.106
SAA4	P35542	1.656 (0.667 - 4.113)	0.277	0.872	0.697 (0.347 - 1.4)	0.311	0.548
IGFALS	P35858; P35858-2	0.563 (0.278 - 1.142)	0.111	0.031	0.498 (0.25 - 0.991)	0.047	0.141
KRT2	P35908	0.884 (0.395 - 1.982)	0.766	0.033	1.549 (0.749 - 3.202)	0.237	0.573
SERPINF1	P36955	0.606 (0.225 - 1.633)	0.322	0.399	0.758 (0.445 - 1.292)	0.310	0.131
CFHR2	P36980-1	1.278 (0.582 - 2.808)	0.541	0.017	0.69 (0.365 - 1.305)	0.255	0.453
PTGDS	P41222	1.04 (0.501 - 2.16)	0.916	0.508	1.164 (0.673 - 2.011)	0.586	0.666
BTD	P43251; P43251-2; P43251-3; P43251-4	0.878 (0.439 - 1.755)	0.712	0.011	0.771 (0.413 - 1.437)	0.414	0.126
AFM	P43652	1.015 (0.603 - 1.709)	0.954	0.011	0.808 (0.511 - 1.277)	0.363	0.026
MASP1	P48740-1	0.965 (0.488 - 1.907)	0.918	0.994	0.816 (0.429 - 1.55)	0.536	0.897
MASP1	P48740-2; P48740-4	0.814 (0.371 - 1.783)	0.606	0.816	1.13 (0.602 - 2.123)	0.702	0.349
SELENOP	P49908	1.227 (0.611 - 2.463)	0.566	0.816	0.839 (0.515 - 1.368)	0.483	0.009
CAMP	P49913	4.335 (1.396 - 13.465)	0.011	0.053	2.078 (0.983 - 4.395)	0.055	0.237
LUM	P51884	0.81 (0.37 - 1.772)	0.597	0.272	0.71 (0.403 - 1.249)	0.235	0.039
CRISP3	P54108-1; P54108-2; P54108-3	1.35 (0.624 - 2.92)	0.446	0.294	1.997 (0.962 - 4.146)	0.063	0.322
APOC4	P55056	2.158 (0.806 - 5.779)	0.126	0.048	0.584 (0.236 - 1.443)	0.244	0.838
PLTP	P55058	0.586 (0.255 - 1.349)	0.209	0.631	0.93 (0.489 - 1.771)	0.827	0.493
CDH13	P55290; P55290-4	1.098 (0.494 - 2.443)	0.819	0.218	1.254 (0.692 - 2.273)	0.455	0.248
DEFA1; DEFA1; DEFA1B	P59665; P59666	0.966 (0.441 - 2.116)	0.931	0.939	0.954 (0.493 - 1.844)	0.889	0.191
ACTB; ACTG1	P60709; P63261	0.78 (0.38 - 1.601)	0.499	0.028	0.677 (0.369 - 1.241)	0.208	0.893
RAP1A; RAP1B	P61224-1; P61224-2; P61224-3; P61224-4; P62834	0.509 (0.194 - 1.337)	0.171	0.109	0.859 (0.369 - 1.998)	0.724	0.706
B2M	P61769	1.277 (0.576 - 2.833)	0.547	0.269	0.815 (0.434 - 1.529)	0.524	0.079
PPIA	P62937	1.785 (0.76 - 4.192)	0.183	0.635	1.725 (0.787 - 3.776)	0.173	0.803
YWHAZ	P63104-1	0.541 (0.245 - 1.194)	0.128	0.394	1.626 (0.83 - 3.188)	0.156	0.252
TUBA1B; TUBA1A; TUBA1C	P68363; P68363-2; Q71U36; Q71U36-2; Q9BQE3	0.589 (0.234 - 1.481)	0.260	0.163	0.494 (0.206 - 1.183)	0.114	0.015
HBB	P68871	1.406 (0.546 - 3.622)	0.481	0.948	0.987 (0.563 - 1.732)	0.966	0.174
HBA1	P69905	1.51 (0.657 - 3.473)	0.332	0.826	1.861 (0.735 - 4.713)	0.190	0.096
GPLD1	P80108	1.991 (0.836 - 4.742)	0.120	0.340	1.1 (0.575 - 2.103)	0.773	0.164
IGLV3-21	P80748	0.617 (0.266 - 1.433)	0.261	0.584	0.779 (0.351 - 1.73)	0.541	0.484
CFHR3	Q02985-1; Q02985-2	0.212 (0.03 - 1.48)	0.118	0.874	0.944 (0.527 - 1.692)	0.848	0.426
CFHR1	Q03591	1.098 (0.495 - 2.436)	0.818	0.483	0.678 (0.351 - 1.308)	0.247	0.751
HGFAC	Q04756	0.703 (0.362 - 1.365)	0.298	0.398	0.736 (0.405 - 1.339)	0.316	0.985
ITIH3	Q06033-1; Q06033-2	0.816 (0.325 - 2.051)	0.666	0.162	1.486 (0.717 - 3.081)	0.286	0.681

Gene name	UniProt ID	ME/CFS with sr-IBS			ME/CFS without sr-IBS		
		Linear		Quadratic effect	Linear		Quadratic effect
PRDX1	Q06830	0.479 (0.186 - 1.236)	0.128	0.685	1.409 (0.695 - 2.853)	0.341	0.686
POLE	Q07864	1.226 (0.529 - 2.841)	0.635	0.120	1.039 (0.569 - 1.897)	0.901	0.478
LGALS3BP	Q08380	1.822 (0.913 - 3.636)	0.089	0.715	0.704 (0.397 - 1.25)	0.232	0.089
EFEMP1	Q12805; Q12805-2; Q12805-3; Q12805-4; Q12805-5	0.989 (0.437 - 2.241)	0.979	0.230	1.016 (0.516 - 1.999)	0.963	0.523
CTTN	Q14247-1; Q14247-2; Q14247-3	1.178 (0.427 - 3.245)	0.752	0.290	1.246 (0.79 - 1.965)	0.343	0.480
HABP2	Q14520-1; Q14520-2	1.529 (0.776 - 3.015)	0.220	0.132	1.186 (0.652 - 2.159)	0.575	0.134
ITIH4	Q14624-1	1.208 (0.709 - 2.057)	0.487	0.021	0.792 (0.508 - 1.237)	0.306	**0.001**
ITIH4	Q14624-2; Q14624-3	0.76 (0.383 - 1.509)	0.433	0.821	1.086 (0.508 - 2.324)	0.830	0.024
PCOLCE	Q15113	0.815 (0.363 - 1.831)	0.620	0.025	0.824 (0.42 - 1.616)	0.575	0.541
PON3	Q15166	1.234 (0.647 - 2.353)	0.523	0.144	2.234 (1.046 - 4.775)	0.038	0.100
TGFBI	Q15582	0.845 (0.439 - 1.625)	0.613	0.555	0.891 (0.548 - 1.45)	0.644	0.116
ECM1	Q16610; Q16610-4	0.75 (0.363 - 1.548)	0.436	0.971	0.826 (0.504 - 1.354)	0.449	0.068
HYI	Q5T013; Q5T013-2; Q5T013-3; Q5T013-4	0.927 (0.439 - 1.955)	0.841	0.455	0.868 (0.542 - 1.39)	0.556	0.088
PLXDC2	Q6UX71-1; Q6UX71-2	0.989 (0.489 - 2.002)	0.976	0.087	1.035 (0.553 - 1.937)	0.913	0.477
PI16	Q6UXB8-1; Q6UXB8-2	1.169 (0.529 - 2.584)	0.700	0.001	0.977 (0.529 - 1.804)	0.942	0.016
FERMT3	Q86UX7; Q86UX7-2	1.167 (0.549 - 2.482)	0.689	0.133	1.582 (0.756 - 3.311)	0.223	0.163
TMPRSS6	Q8IU80-1; Q8IU80-4; Q8IU80-5	1.756 (0.965 - 3.197)	0.065	0.038	1.416 (0.796 - 2.52)	0.236	0.138
PATJ	Q8NI35; Q8NI35-2; Q8NI35-3; Q8NI35-4; Q8NI35-5	0.544 (0.303 - 0.977)	0.042	0.036	0.778 (0.488 - 1.241)	0.293	0.200
SUN3	Q8TAQ9-1; Q8TAQ9-2; Q8TAQ9-3	1.018 (0.512 - 2.023)	0.960	0.210	0.592 (0.345 - 1.016)	0.058	0.241
CFHR4	Q92496; Q92496-2	1.217 (0.599 - 2.474)	0.587	0.049	1.263 (0.624 - 2.554)	0.515	0.543
PRG4	Q92954-1; Q92954-3; Q92954-6	1.926 (0.822 - 4.512)	0.131	0.914	0.707 (0.338 - 1.476)	0.356	0.312
CPB2	Q96IY4	0.988 (0.473 - 2.067)	0.975	0.178	1.255 (0.693 - 2.27)	0.453	0.001
CNDP1	Q96KN2	2.607 (1.135 - 5.992)	0.024	0.404	1.535 (0.82 - 2.873)	0.180	0.734
FCRL3	Q96P31-1; Q96P31-2; Q96P31-3; Q96P31-4; Q96P31-5; Q96P31-6; Q96P31-7	0.43 (0.14 - 1.318)	0.140	0.458	0.307 (0.106 - 0.889)	0.030	0.170
PGLYRP2	Q96PD5; Q96PD5-2	1.149 (0.583 - 2.267)	0.688	0.311	0.962 (0.643 - 1.439)	0.853	0.039
MENT	Q9BUN1	0.721 (0.324 - 1.605)	0.423	0.185	0.964 (0.527 - 1.762)	0.906	0.787
COLEC11	Q9BWP8; Q9BWP8-10; Q9BWP8-2; Q9BWP8-3; Q9BWP8-4; Q9BWP8-5; Q9BWP8-6; Q9BWP8-7; Q9BWP8-8; Q9BWP8-9	0.585 (0.245 - 1.397)	0.227	0.960	0.599 (0.279 - 1.286)	0.189	0.752
CFHR5	Q9BXR6	2.543 (0.952 - 6.79)	0.063	0.848	1.467 (0.7 - 3.074)	0.309	0.798
SH3BGRL3	Q9H299	0.842 (0.419 - 1.689)	0.627	0.152	0.846 (0.443 - 1.617)	0.615	0.393
CRTAC1	Q9NQ79; Q9NQ79-2; Q9NQ79-3	0.333 (0.134 - 0.831)	0.018	0.001	0.592 (0.311 - 1.128)	0.111	0.971
C1RL	Q9NZP8	1.568 (0.783 - 3.138)	0.204	0.428	0.761 (0.352 - 1.647)	0.489	0.708
FETUB	Q9UGM5-1	1.146 (0.558 - 2.355)	0.711	0.580	0.679 (0.341 - 1.353)	0.272	0.644
SERPINA10	Q9UK55	1.483 (0.708 - 3.108)	0.296	0.827	0.864 (0.474 - 1.574)	0.633	0.452
TLN1	Q9Y490	0.947 (0.459 - 1.953)	0.883	0.545	0.566 (0.327 - 0.98)	0.042	0.904
FARP1	Q9Y4F1; Q9Y4F1-2	N/A	N/A	N/A	0.965 (0.477 - 1.951)	0.922	0.436
PCDHGC5	Q9Y5F6-2	0.959 (0.453 - 2.026)	0.912	0.757	1.451 (0.713 - 2.951)	0.303	0.090
FCGBP	Q9Y6R7	1.224 (0.622 - 2.412)	0.558	0.599	1.147 (0.628 - 2.096)	0.654	0.066
