# Supplementary material for: Plasma proteomic profiling suggests an association between antigen driven clonal B cell expansion and ME/CFS
Source: PLoS One. 2020 Jul 21;15(7):e0236148. doi: 10.1371/journal.pone.0236148 (PMC7373296; doi:10.1371/journal.pone.0236148)
Supplement: S5 Table — ME/CFS: myalgic encephalomyelitis/chronic fatigue syndrome, sr-IBS: self-reported irritable bowel syndrome, AUC: area under the curve, CI: confidence interval. (PDF) [file pone.0236148.s007.pdf]

Supplementary Table 5. Assessment of predictive power of the classifiers Lasso/Logistic regression, Random Forests, and XGBoost for all ME/CFS patients, ME/CFS patients with sr-IBS, and ME/CFS patients without sr-IBS, when compared to the control group.

	Classifier	AUC	95% CI	
**All ME/CFS**	Lasso/Logistic Regression	0.774	0.654	0.861
	Random Forests	0.820	0.691	0.895
	XGBoost	0.838	0.731	0.908
**ME/CFS with sr-IBS**	Lasso/Logistic Regression	0.806	0.647	0.904
	Random Forests	0.804	0.561	0.927
	XGBoost	0.846	0.703	0.927
**ME/CFS without sr-IBS**	Lasso/Logistic Regression	0.754	0.600	0.862
	Random Forests	0.780	0.616	0.885
	XGBoost	0.774	0.637	0.869
